# ProtDCal: A program to compute *general-purpose*-numerical descriptors for sequences and 3D-structures of proteins

**DOI:** 10.1186/s12859-015-0586-0

**Published:** 2015-05-16

**Authors:** Yasser B Ruiz-Blanco, Waldo Paz, James Green, Yovani Marrero-Ponce

**Affiliations:** 1 Unit of Computer-Aided Molecular “Biosilico” Discovery and Bioinformatic Research (CAMD-BIR Unit), Facultad de Química y Farmacia, Universidad Central “Marta Abreu” de Las Villas, Road to Camajuani km 5 ½, Santa Clara, CP: 54830 Villa Clara Cuba; 2 Department of Systems and Computer Engineering, Carleton University, Ottawa, ON Canada; 3 Centre of Informatics Studies (CEI), Universidad Central “Marta Abreu” de Las Villas, Road to Camajuani km 5 ½, Santa Clara, CP:54830 Villa Clara Cuba; 4 Grupo de Investigación Microbiología y Ambiente (GIMA). Programa de Bacteriología, Facultad Ciencias de la Salud, Universidad de San Buenaventura, Calle Real de Ternera, Cartagena (Bolivar), Colombia

**Keywords:** ProtDCal, Protein feature generation, Protein descriptors, Data mining, Protein function modelling

## Abstract

**Background:**

The exponential growth of protein structural and sequence databases is enabling multifaceted approaches to understanding the long sought sequence-structure-function relationship. Advances in computation now make it possible to apply well-established data mining and pattern recognition techniques to these data to learn models that effectively relate structure and function. However, extracting meaningful numerical descriptors of protein sequence and structure is a key issue that requires an efficient and widely available solution.

**Results:**

We here introduce ProtDCal, a new computational software suite capable of generating tens of thousands of features considering both sequence-based and 3D-structural descriptors. We demonstrate, by means of principle component analysis and Shannon entropy tests, how ProtDCal’s sequence-based descriptors provide new and more relevant information not encoded by currently available servers for sequence-based protein feature generation. The wide diversity of the 3D-structure-based features generated by ProtDCal is shown to provide additional complementary information and effectively completes its general protein encoding capability. As demonstration of the utility of ProtDCal’s features, prediction models of N-linked glycosylation sites are trained and evaluated. Classification performance compares favourably with that of contemporary predictors of N-linked glycosylation sites, in spite of not using domain-specific features as input information.

**Conclusions:**

ProtDCal provides a friendly and cross-platform graphical user interface, developed in the Java programming language and is freely available at: http://bioinf.sce.carleton.ca/ProtDCal/. ProtDCal introduces local and group-based encoding which enhances the diversity of the information captured by the computed features. Furthermore, we have shown that adding structure-based descriptors contributes non-redundant additional information to the features-based characterization of polypeptide systems. This software is intended to provide a useful tool for *general-purpose* encoding of protein sequences and structures for applications is protein classification, similarity analyses and function prediction.

**Electronic supplementary material:**

The online version of this article (doi:10.1186/s12859-015-0586-0) contains supplementary material, which is available to authorized users.

## Background

The enormous growth of protein sequence databases has become a powerful driving force for data mining studies of protein function prediction or protein classification. Databases such as UniProt (http://www.uniprot.org/) [1] and GenBank (http://www.ncbi.nlm.nih.gov/genbank/) [2] count the number of available protein sequences in the tens of millions, providing a large reservoir of information for such studies. Furthermore, the Worldwide Protein Data Bank (www.wwpdb.org) [3] now holds nearly 100 000 3D structures, while many more can be inferred using homology modeling and *ab initio* prediction, even at genome-wide scale [4]. Pattern classification and data mining techniques require numerical feature data summarizing aspects of protein sequence and structure. Given appropriate feature selection methods, we expect to achieve greater predictive accuracy if the methods are provided with more and diverse input features. Such numerical features which describe aspects of molecular structure are widely known as *descriptors* in fields outside of proteomics. In the field of cheminformatics, millions of molecular descriptors (MDs) for small-to-mid sized compounds [5] are currently implemented in software packages such as: DRAGON, TOMOCOMD-CARDD, PADEL, CDK descriptor calculator, ADRIANA CODE, CODESSA-PRO and CERIUS [6-17]. Rather than developing different features for each application, these MDs instead provide a rich application-independent general numerical representation of the molecule, with each MD relating to a different aspect of the molecule. By applying appropriate feature selection, relevant subsets of the same overarching set of MDs may be extracted to develop analytical approaches to solve many diverse problems.

A number of groups have proposed developing such sets application-independent descriptors for the field of proteomics [18], however, we remain limited to the order of a few thousand descriptors to encode protein sequences [19,20] and even fewer for protein 3D-structures [21-24]. Currently PROFEAT [19,25] (http://bidd.cz3.nus.edu.sg/cgi-bin/prof/protein/profnew.cgi), PROTEIN RECON [26] (http://reccr.chem.rpi.edu/Software/Protein-Recon/Protein-Recon-index.html) and PseAAC (http://www.csbio.sjtu.edu.cn/bioinf/PseAA/) [27] are the most widely used publicly available servers for computing large numbers of sequence-based protein physicochemical features. However, these tools lack: *i*) large capacity for descriptor generation (as compared with programs for MD generation); *ii*) portability and cross-platform code (many are limited to a webserver interface), *iii*) generalization, in the sense of including not just their own descriptors (particularly PROTEIN RECON and PseAAC), and *iv*) the possibility to also generate descriptors relating to protein 3D structure, when such structure is known.

We have recently developed a model intended to describe protein folding stability and its contributing factors, i.e. configurational entropy, close packing interactions, and the hydrophobic effect [28]. Additionally, we have introduced a physics-based formalism to score protein structural models [29]. We here introduce a new feature generation program called ProtDCal (PROTein Descriptors CALculation program), which implements these new approaches together with several physicochemical properties of amino acids, and structural descriptors with proven capability to predict protein folding kinetic properties [21-24]. This program is freely available, supports multiple computing platforms, and provides a graphical user interface. ProtDCal is capable of generating tens of thousands of descriptors for a single protein structure (considering both, sequence-based and structure-based descriptors), thereby helping to close the gap between the diversity of descriptors available for the study of small molecules (cheminformatics) and proteins (proteomics).

In the present study, the resulting features are assessed in terms of relevancy and redundancy in three different studies: 1) variability analysis along the protein dataset (relevancy), based on Shannon’s entropy [30,31]; 2) linear-independence (redundancy) of the codified information by using Principal Component Analysis (PCA) [32] within ProtDCal descriptors, and 3) redundancy of descriptors among all three software packages. In order to carry out the analyses presented in this report, the default configurations of PROFEAT [19] and PROTEIN RECON were used as a source of the state-of-the-art in sequence-based features. PseAAC was not included in this comparative analysis because a representative module for computing pseudo amino acid composition features is already implemented within PROFEAT. Lastly, we demonstrate that ProtDCal is highly computationally efficient and is able to calculate thousands of features within 1 s for a typical protein sequence or structure.

In the following sections the term *index* refers to a property or value which has been calculated or measured for a single residue, while *feature* or *descriptor* refers to the final result of a procedure which generates a value associated with a specific group (subset) of amino acids using an optional aggregation function and weighting operator.

## Implementation

**Figure 1:**
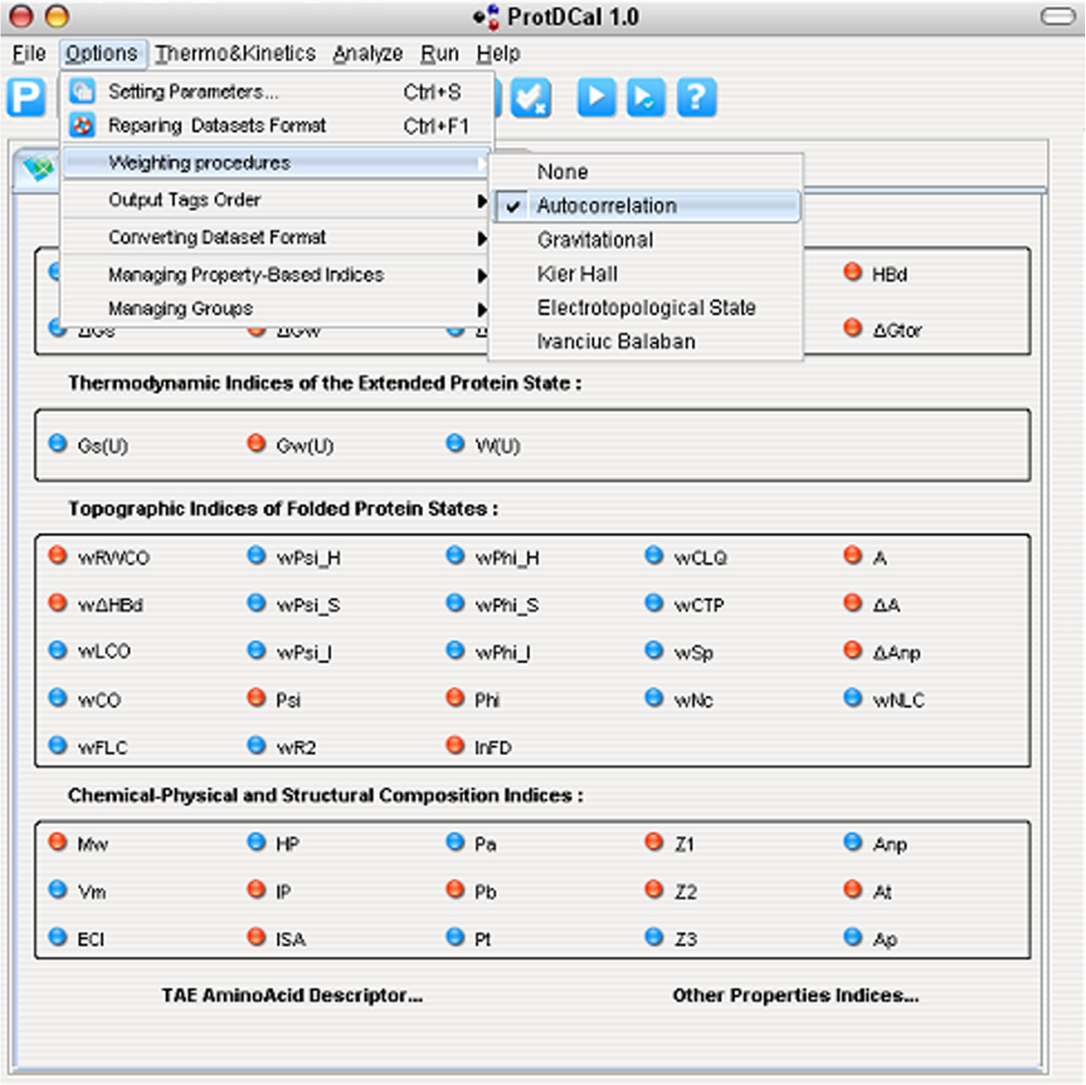
GUI layer corresponding to the configuration of indices and weighting operators.

ProtDCal provides a friendly graphical user interface (GUI), see Figure 1, which generates descriptors for groups of residues (including the whole protein as the largest possible group). The program accepts two input file formats: PDB and FASTA/multi-FASTA. In the former case, the full descriptor generation capability of the program is enabled, while inputting FASTA files will only enable the sequence-based subset of indices. The program calculates the requested features and creates two tab-delimited files (*_AA.txt and *_Prot.txt). These files contain the compendium of all the residue-level indices and the group-level descriptors, respectively, for each input protein.

The software is implemented in Java (JDK version 1.7) as it provides cross-platform support for any system where a Java Virtual Machine (JVM) is available. The Chemical Development Kit (CDK) library [33] (version 1.4.19) was employed within ProtDCal, mainly for the manipulation of protein input data.

Most of the 3D-structural descriptors published to date typically capture information relating to the entire protein structure. These features have been largely used to show their correlation with folding properties such as the folding rate constant [34-36]. However, restricting oneself to descriptors of the entire protein structure limits the possibility of generating meaningful numbers to encode different structural characteristics of a single protein. The use of groups (subsets – see below) of amino acids to generate protein descriptors permits a combinatorial strategy to achieve a wide spectrum of features for each input protein.

The strategy for calculating indices and descriptors is divided into four hierarchical levels i) choice of index, ii) choice of weighting procedure, iii) choice of residue group, and iv) choice of aggregation function:

### Choice of indices

This first user selection level provides to the user a set of criteria which generate indices for each residue of a protein. The formal definition of these indices is summarized in Additional file 1: Tables SM-1 to SM-4. The indices are organized into three main families:

i-1)Thermodynamics, which are novel physics-based indices designed to describe the main factors involved in protein folding stability [28,37]. These indices deal with residue-residue electronic interactions, Van der Waals interactions, dihedral torsion potential, backbone hydrogen bond formation, and hydrophobic effect. Both 3D-structure-based and sequence-based thermodynamic indices are provided by ProtDCal.

i-2)Topographic, which include versions of structural descriptors, most of them, originally, designed to describe protein folding rate, e.g. relative contact order (CO) [38], long range order (LRO) [22], total contact distance (TCD) [23], contact number (Nc) [34], cliquishness (CLQ) [35], among others. All these features were originally defined as sums/averages over all residues of a protein; here they were redefined to provide a value for each residue of a protein, such that if the contributions of each residue were summed up the formula would coincide with its original definition. Most of these type of features uses inter-residue contacts as the basis of their formalisms. Consequently, we included an adaptable weighting coefficient to differentiate inter-residue contacts to achieve increased diversity among the computed indices (see description of ω_*ij*_ in equations 4 & 5 below). To illustrate how these descriptors were transformed into per-residue indices, we next describe the transformation of the contact order (CO) originally defined by Plaxco *et al.* [39]. According the original definition:

1 $$ CO=\frac{1}{N{N}_c}{\displaystyle \sum_{c=1}^N\varDelta {N}_a} $$

where, *N* represents the length of the protein, *N*_*c*_ the number of contacts in a protein and ΔN_a_ the sequence separation between a pair of residues involved in a contact. The transformation to obtain our weighted-residue-level Contact Order (*wCO*_*i*_) is as follow:

2 $$ CO=\frac{1}{N{N}_c}{\displaystyle \sum_{a= 1}^{N_c}\varDelta {N}_a}=\frac{1}{N{N}_c}{\displaystyle \sum_{i= 1}^{N\mathit{\hbox{-}} 1}{\displaystyle \sum_{j>i}^N\Delta {N}_{ij}}{\delta}_{ij}} $$

where, δ_*ij*_ is a binary variable indicating if residue pair (*i*,*j)* satisfy the two contact conditions: i) the pair of residues have spatial distances less than *d,* and ii) the topological distance is greater than *t*. The default values are set to *d* = 8 and *t* = 4; users may change these thresholds via the PROTCAL interface. The transformation follows as:

3 $$ CO=\frac{1}{N{N}_c}{\displaystyle \sum_{i= 1}^{N\mathit{\hbox{-}} 1}{\displaystyle \sum_{j\mathit{\hbox{-}}i}^N\varDelta {N}_{ij}{\delta}_{ij}}={\displaystyle \sum_{i= 1}^N\frac{1}{2N{N}_c}}{\displaystyle \sum_{j= 1;j\mathit{\ne}i}^N\varDelta {N}_{ij}{\delta}_{ij}S}} $$

Substituting the sequence separation parameter, Δ*N*_*ij*_, by a general weighting parameter ω_*ij*_, we obtain:

4 $$ wCO={\displaystyle \sum_{i= 1}^N\frac{1}{2N{N}_c}}{\displaystyle \sum_{j= 1;j\mathit{\ne}i}^N{\omega}_{ij}{\delta}_{ij}} $$

Finally, the redefined residue-level weighted Contact Order index is given by:

5 $$ wC{O}_i=\frac{1}{2N{N}_c}{\displaystyle \sum_{j= 1;j\mathit{\ne}i}^N{\omega}_{ij}{\delta}_{ij}} $$

The parameter ω_*ij*_ represents a weighting coefficient for each pair of residues. This parameter is computed as the product, ω_*i*_ω_*j*_, of the property for each interacting residue, where any of 12 amino-acid properties covering structural, physical-chemical features may be selected (see *Property-based* indices for details). Additionally, the sequence separation parameter (Δ*N*_*ij*_), which is present in the original definition, and the absence of weighting (i.e. ω_*ij*_ = 1), are implemented as the possible criteria for weighting a contact.

i-3) Property-Based indices, containing a set of empirical indices with fixed values for each type of residue. These measures cover a wide range of amino-acid properties, such as the Kyte-Doolittle scale of hydrophobicity, which has been used to predict potentially exposed regions and transmembrane domains [40]; the so-called *principal properties* or *z-values* [41]: *z1* related to hydrophilicity, *z2* related to steric features, and *z3* dealing with polarity; Levitt’s probabilities of adopting an alpha helix, P_α_, a beta sheet, P_β_, or a turn conformation, P_τ_, [42]; as well as classic features such as the isoelectric point and the mass. Additional file 1: Table SM-4 summarizes the values of each of these properties for every residue type. The redundancy among some of these aminoacid properties have study with a benchmarking approach [43,44]. An additional set of 147 Transferable Atomic Equivalent (TAE) indices are provided, as originally proposed by Breneman *et al.*, the group responsible for the development of PROTEIN RECON server, and as implemented in the CDK library [12,33]. The TAE were computed based on the quantum theory of atoms in molecules, which has been a successful approach to study molecular properties related to electron density distribution. Additional file 2 provides the compendium of TAE indices values for each residue type.

### Weighting procedures

Once the indices are selected, five classic cheminformatics algorithms were implemented to allow the modification of the intrinsic index values of residues according their particular neighborhood: Autocorrelation, Kier-Hall, Electrotopological State, Ivanshiuc-Balaban [45], and Gravitational-like operators. Additional file 1: Table SM-10 summarizes these formalisms, while an in-depth description of each can found in the Handbook of Molecular Descriptors of Todeschini and Consonni [5]. In order to show how these weighting procedures are applied to the calculation of indices, we include a comprehensive example of the Autocorrelation weighting operation, which is defined in ProtDCal as:

6 $$ A{C}_i^k={\displaystyle \sum_{j\ge 1}^N{L}_i{L}_j\delta \left({d}_{ij}-k\right)} $$

where, *L*_*i*_ represents the value of a particular index for residue *i*, the parameter *k* is a topological distance cut-off, the topological distance d_ij_ = |j - i|, and δ is the Dirac delta function, and *N* is the total number of residues in the protein chain. According to this operator, the neighborhood of a residue *i* would be defined by the two residues *j* with a sequence separation of *k* residues with respect to residue *i*. The results of this procedure applied to the topographic index *logarithm of the Folding Degree (lnFD)* for an eight-residue fragment corresponding to residues 61 to 68 of a human prion protein (PDB ID 1oeh) [46] are illustrated in Table 1.

**Table 1:** **Illustration of the application of the Autocorrelation operator to the index** ***lnFD*** **using the parameter k = 2**

***Residues***	***Index value (lnFD***_***i***_ ***)***	***Label***	***Autocorrelation procedure (k =2)***	***Updated index value (lnFD_AC***_***i***_ ***)***
*1OEH_aa1_HIS*	−3.53E-02	L_1_	L_1_’ = L_1_L_3_ =	2.93E-04
*1OEH_aa2_GLY*	−1.54E-02	L_2_	L_2_’ = L_2_L_4_ =	1.39E-04
*1OEH_aa3_GLY*	−8.31E-03	L_3_	L_3_’ = L_3_L_1_ + L_3_L_5_ =	3.72E-04
*1OEH_aa4_GLY*	−9.01E-03	L_4_	L_4_’ = L_4_L_2_ + L_4_L_6_ =	2.05E-04
*1OEH_aa5_TRP*	−9.43E-03	L_5_	L_5_’ = L_5_L_3_ + L_5_L_7_ =	2.88E-04
*1OEH_aa6_GLY*	−7.36E-03	L_6_	L_6_’ = L_6_L_4_ + L_6_L_8_ =	3.09E-04
*1OEH_aa7_GLN*	−2.23E-02	L_7_	L_7_’ = L_7_L_5_ =	2.10E-04
*1OEH_aa8_PRO*	−3.30E-02	L_8_	L_8_’ = L_8_L_6_ =	2.43E-04

The structure of an octapeptide from a mammalian prion protein (PDB code: 1OEH) was employed for calculations of *lnFD* values.

As represented in Table 1, the computed index of each residue is consequently modified according a defined neighbourhood, which depends on the particular weighting procedure and its corresponding parameters (here, *t*). Figure 1 depicts the GUI’s layer corresponding with the indices implemented in ProtDCal as well as the weighting operators menu of the application.

### Groups

**Figure 2:**
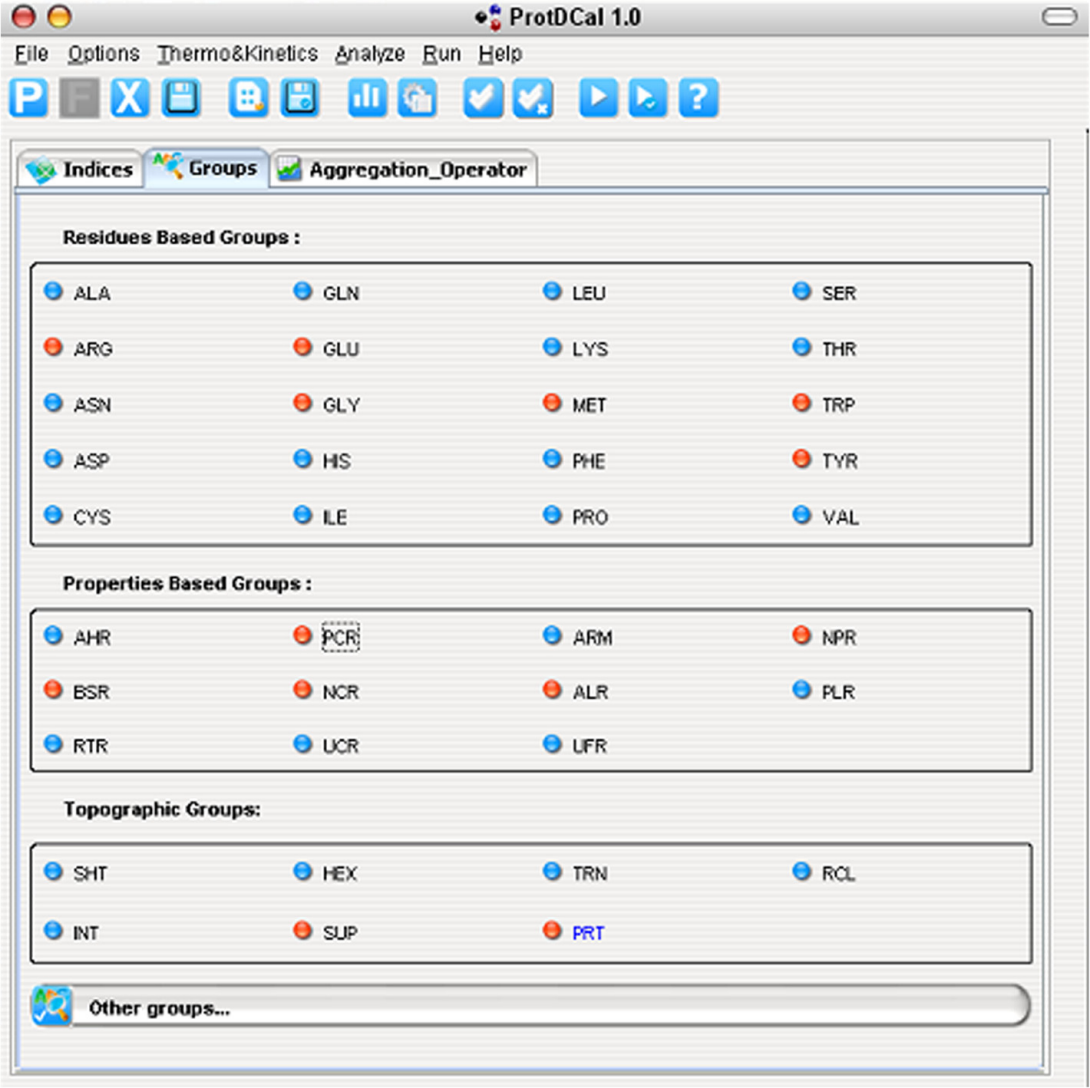
GUI layer corresponding to the configuration of groups of residues.

As mentioned above, the user is able to compute descriptors for various groups (subsets) of residues, according their type, properties, or structural arrangement. Each group is used to build an array of values for each selected index in the previous step. The three types of groups are:

iii-1) *Type-based groups*. These groups correspond to all residues of a single natural amino acid. Each of these groups will comprise all the residues, of the same type, within the protein.

iii-2) *Property-based groups*. These groups cover most standard amino acid classifications according their physicochemical properties, including: polar, basic, acidic, aromatic, etc. Additional file 1: Table SM-11 summarizes the definitions of these groups.

iii-3) *Structure-based groups*. These groups are based on 3D arrangements of residues in the protein including: the internal residues (INT), determined by an adaptable cutoff of the percentage of their surface area deemed to be solvent accessible; the superficial residues (SUP), determined by the same cutoff mentioned above; the residues in alpha helix (HEX), the list of the residues involved in helix motifs must be explicitly defined in the PDB file; the residues in beta sheet (SHT), the list must be explicitly defined in the PDB file, the coil regions (RCL) this group comprises all residues neither in helices nor beta-sheets fragments; and finally the residues in beta turn (TRN), they list must be explicitly defined in the PDB file. Selecting the entire protein (PRT) as a special group comprising all the residues in the protein is also possible. Figure 2 shows the GUI’s layer associated with the selection of different groups of residues.

### Aggregation operators

This final configuration level is intended to combine the index values for a group of residues into a unique value for every combination of index, group and aggregation operator. The use of these aggregation operators have been successfully applied, recently, by Marrero-Ponce *et al.* to generalize the local vertex invariants (LOVIs) vector to global (or fragment-based) features of organic molecules [47-49]. Here this strategy is also applied as a suitable way to enhance the protein features generation. This layer of the program is divided into four panels:

iv-1) *Distance measures* panel, which contains the first three Minkowsky norms comprising the Manhattan distance, Euclidean distance and third Minkowsky norm. Additional file 1: Table SM-6 summarizes the formulae and descriptions of these measures.

iv-2) *Measures of central tendency* panel, this panel includes aggregation functions such as arithmetic mean, geometric mean, harmonic mean, etc. which provide an average of the entries in the array of indices. Additional file 1: Table SM-7 summarizes these measures.

iv-3) *Measures of statistical dispersion* panel, this panel encloses statistics such as variance, coefficient of variation, skewness, etc. which encode different characteristics of the distribution of values in the index array. Additional file 1: Table SM-8 summarizes these measures.

iv-4)* Measures based on Information Theory*, this panel contains three classical procedures [5] derived from the information theory which describe the entropy of the distribution of an index values within a given group. These measures are: the Total Information Content, the Mean Information Content, and the Standardized Information Content. Additional file 1: Table SM-9 summarizes the formulae and descriptions of these measures.

**Figure 3:**
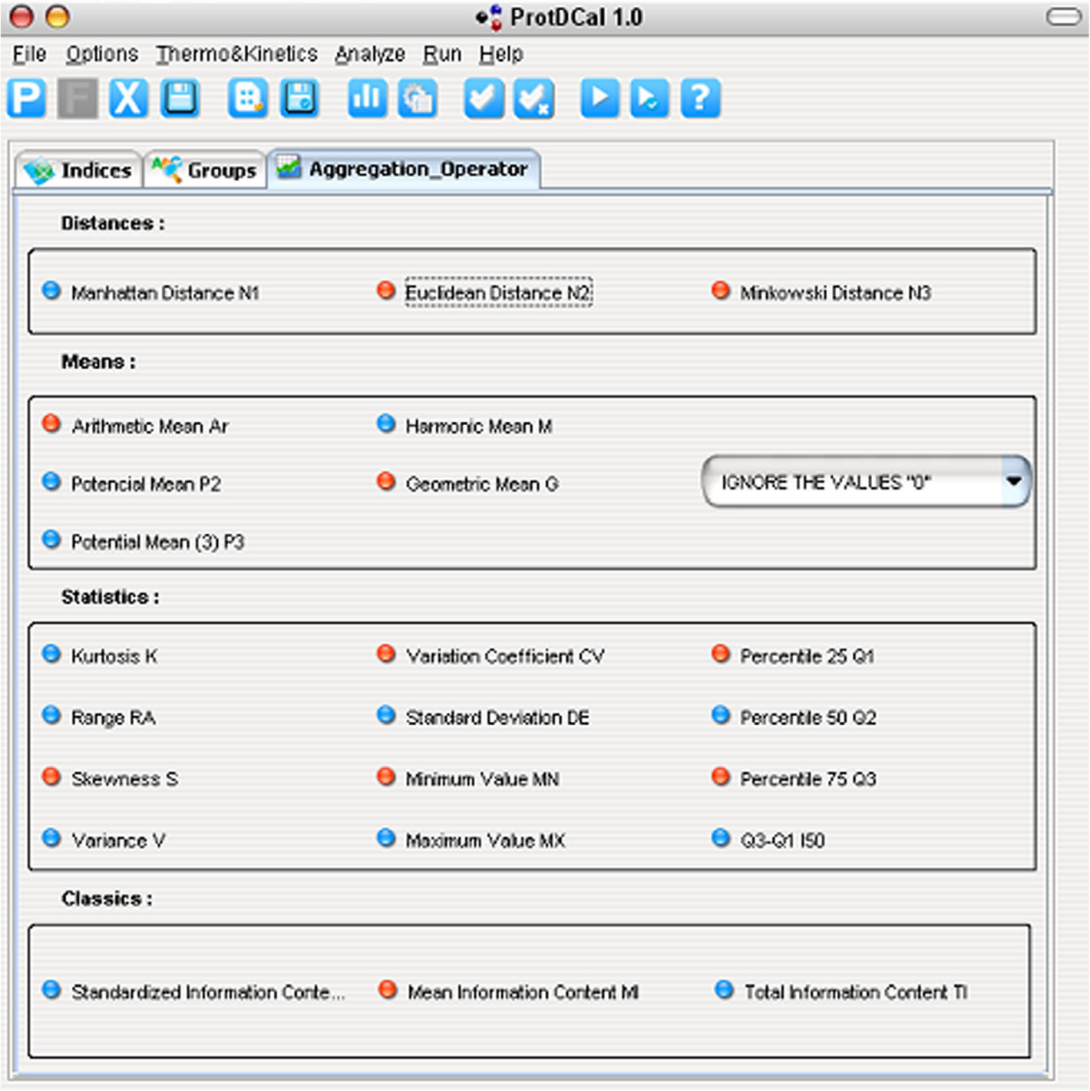
GUI layer corresponding to the configuration of aggregation operators.

The Figure 3 shows the GUI’s layer associated with the aggregation operators.

### Additional functionalities of ProtDCal

In addition to computing a wide diversity of sequence- and structure-based descriptors, ProtDCal has an special menu called *Thermo&Kinetics*, which permits the application of empirical models designed by some of the authors to predict protein folding free energy [28] and its different contributions: the loss of configurational free energy, the hydrophobic effect free energy and the close-packing interaction free energy; as well as a model for predicting folding rate constant [50], and a scoring potential intended to discriminate among near-native and non-native structural decoys [29]. All of these models require PDB files as inputs. Additional file 1: Table SM-5 provides a summary of the definition and description for all these models.

Lastly, a graphic plotting menu is provided, which allows the possibility of plotting index profiles along the protein sequence and index histograms within a protein. ProtDCal can also compute distance matrices for datasets of proteins structures or sequences based on the user-selected descriptors. This could provide valuable input for protein similarity rankings and classification models.

A comprehensive tutorial and discussion of the theoretical background of the various implemented formalisms are provided in the Help Menu of the ProtDCal application.

## Results and discussion

In order to evaluate the descriptors generated by our software, as well as to perform comparison tests with PROFEAT and PROTEIN RECON servers, a dataset of 874 proteins was obtained from the RCSB PDB [51] on February 24th, 2014 by searching for single-chain monomer proteins, without DNA, RNA or other non-protein chemical entities, with sequence length between [50,500] and resolution of at most 2.0 Å. Homologues were removed at 30% sequence identity, resulting in a clean and non-redundant set of protein structures covering a wide range of protein lengths and forming a representative sample of proteins known to date. These data provides a suitable scaffold to evaluate properties such as variability and redundancy of ProtDCal’s descriptors. A complete list of proteins can be found in Additional file 1: Table SM-12.

First, we validate the lack of redundancy between our sequence-based and structure-based features using principal component analysis (PCA) for factor extraction and the varimax normalized method to rotate the matrix of components, as implemented in the software package SPSS 21. Next, to measure the relevancy of our descriptors and those of other servers, a variability test was carried out using the information-theoretic approach proposed by Godden [52,53]. This test was used to measure the potential of descriptors to differentiate among proteins in the dataset described above. Lastly, we assess the diversity among our features and also with those generated by other programs. We here leverage the orthogonality of factors generated by PCA to assert that descriptors populating different factors (with absolute loading values greater than 0.70) are considered to contain significantly different information.

Three studies were carried out in order to assess the quality of descriptors generated by our application and to compare our features with existing available servers with similar purpose.

### Analysis of redundancy between 3D and sequence-based ProtDCal’s descriptors

One of the potentially valuable features introduced by ProtDCal is its capability to generate a vast variety of novel 3D-structural descriptors for proteins. In that sense, we started by comparing the intrinsic redundancy of the two largest families of indices in ProtDCal i.e., the structure-based and the sequence-based indices. To conduct this assay, we computed 45494 sequence-based features, leaving aside the twenty residue-type groups and all weighting operators. This dataset was filtered by means of the Shannon entropy variability test to eliminate those trivial attributes with zero or almost zero variance. Next, a subset of 999 descriptors was randomly selected to serve as a representative sample of the sequence-based family of ProtDCal indices. The same procedure was followed to select 999 structure-based features from a pool of all 25231 possible 3D indices (hydrophobicity was used as weighting coefficient of the inter-residue contact in topographic indices), and no weighting operations were used.

Finally, the sequence- and structure-based features were united into a single data set with a total of 1998 features. PCA was applied to evaluate the overlap in the information content between these two sets of indices (linear independence). A total of 159 principal components were extracted, accounting for 95% of total explained variance. Additional file 1: Table SM-13 summarizes the percentage of variance explained by each of the 159 extracted components. The filtered rotated component matrix for this analysis is provided in Additional file 3 (only loading coefficients with absolute values greater than 0.7 are shown). The results indicate that there exists some degree of overlapping information given that both types of indices significantly populate factors 1, 6, and 14 (see component matrix in Supplementary Material). Nonetheless, the structure-based descriptors have high loadings in 94 Factors while the sequence-based descriptors only populate 16 factors with high loadings. This implies that there is a large amount of information captured by the structure-based ProtDCal descriptors that is not present in sequence-based descriptors. Additionally, the present analysis built a total of 46 factors which don’t significantly correlate with any individual descriptor in the composed dataset. This means that the combination of 3D and sequence-based descriptors of ProtDCal is capable of capturing information that is not contained in any single descriptor, but is disaggregated among the entire data.

This experiment validates the enormous amount of new information available for data mining studies by using 3D descriptors introduced in ProtDCal, when compared to traditional sequence-based feature extraction servers. The following experiments compare the sequence-based features of ProtDCal with those features generated by the sequence-based servers PROFEAT and PROTEIN RECON.

### Comparison of variability among sequence-based descriptors of ProtDCal versus descriptors of PROFEAT and PROTEIN RECON

Variability or relevance is the first characteristic which should be validated in any new descriptor, i.e. valuable descriptors should vary among different sequences. To address this comparison, all the descriptors of the default configuration of PROFEAT (1130 descriptors) and PROTEIN RECON (141 descriptors) were used. Given that these servers generate only sequence-based features, we here restricted ourselves to ProtDCal descriptors of to this kind. The twenty residue-type groups implemented in ProtDCal were excluded in order to diminish the amount of data in the analysis. The size weighting operators were applied separately, leading to six sub-datasets of 45494 descriptors each.

**Figure 4:**
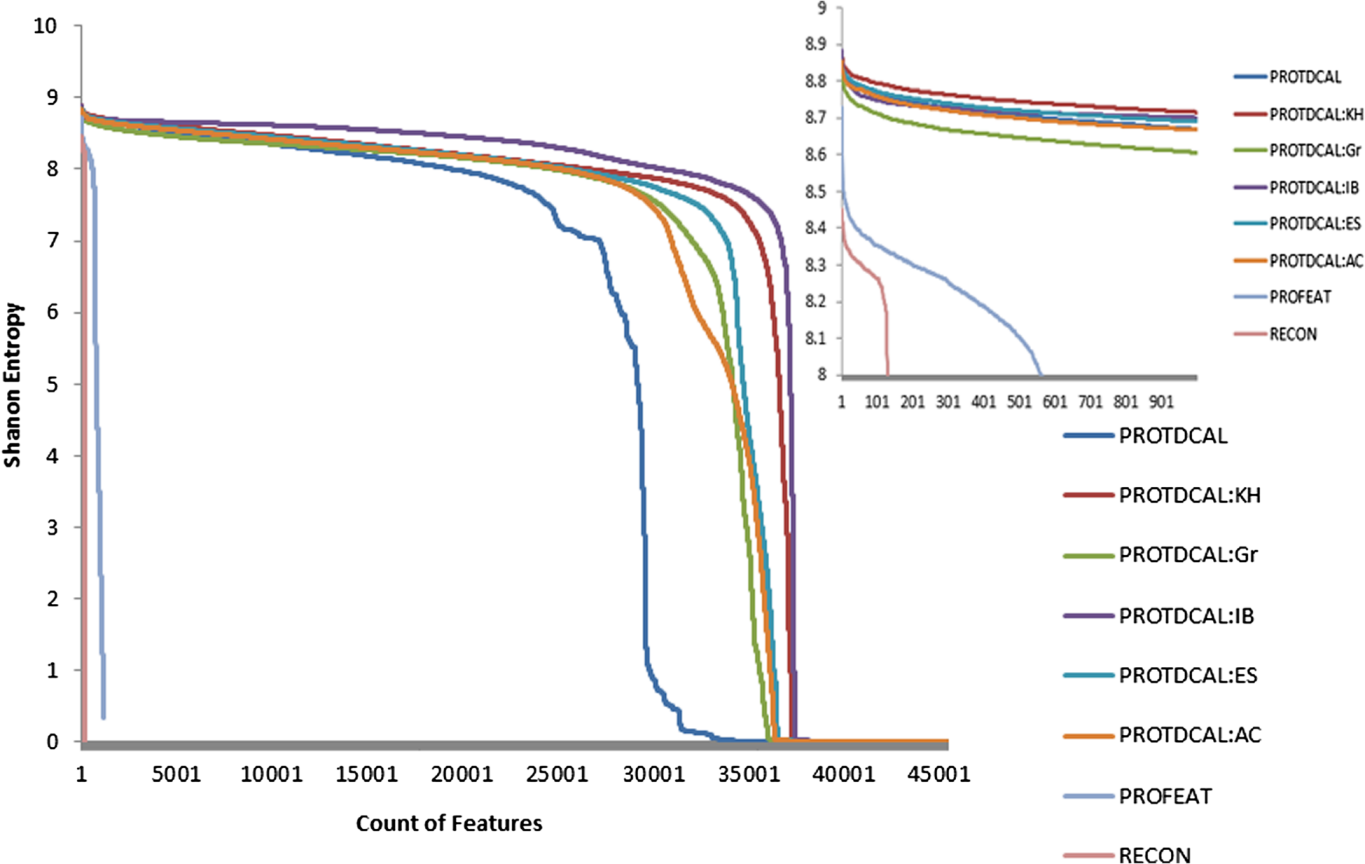
Comparison of Shannon entropy values of sequence-based features computed with PROFEAT, PROTEIN RECON, and the different weighting operators of ProtDCal: autocorrelation (AC), Kier-Hall (KH), electrotopological state (ES), Ivanshiuc-Balaban (IB) and gravitational-like (GR) operators.

Shannon entropy was used to assess the variability of the eight datasets of descriptors (six PROTCAL plus PROFEAT and PROTEIN RECON). Additional file 4 provides the Shannon entropy rankings for the eight datasets. Figure 4 plots the number of descriptors (x-axis), in each dataset, with entropy values larger than each value in the y-axis.

**Figure 5:**
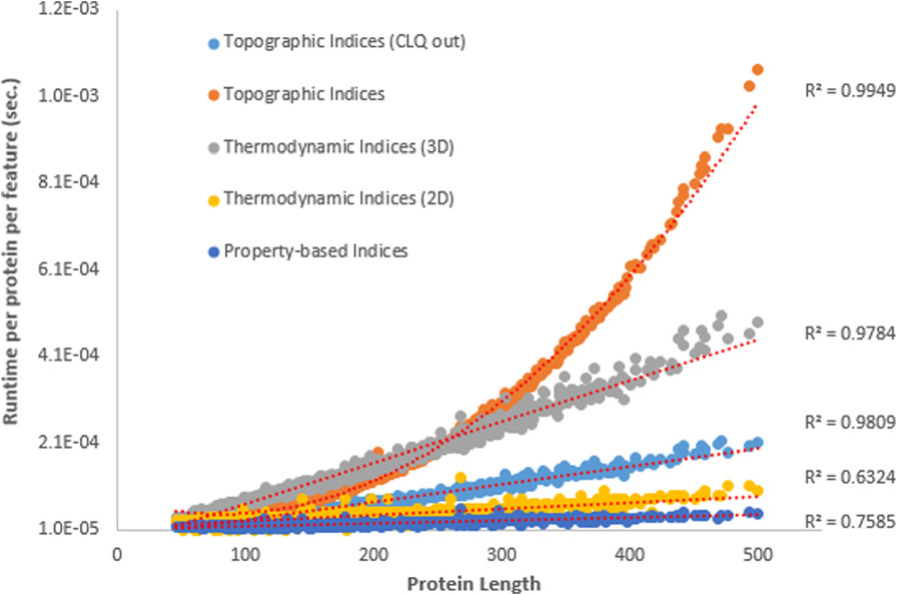
Comparison of runtime values per protein per feature versus protein lengths by using four families of protein features: structure-based thermodynamic indices, sequence-based thermodynamic indices, topographic indices (weighted by topological distance), and amino-acid-property-based indices (TAE-derived indices excluded).

This figure shows that all five weighting operators implemented within ProtDCal and the unweighted data have a significantly larger numbers of relevant descriptors than the other applications. Also, it is shown that, by using weighting operators, a larger number of relevant descriptors can be generated (see shift in weighted curves compared to unweighted curve in Figure 4). Closer inspection reveals that the Kier-Hall weighting operator produces the top relevant descriptors (see inset picture in Figure 5), but the Ivanciuc-Balaban operator provides the most stable rate of relevancy among all descriptors.

To ensure that the six weighting operators are not redundant with each other, and that they actually provide useful independent features, an experiment was conducted as follows. The feature (HP_PRT_Q2) resulting from the combination of the hydrophobicity scale of Kyte and Doolittle (HP), the whole protein sequence taken as a group (PRT), and the second quartile of the index’s distribution used as aggregation operator (Q2), was computed with the five weighting operators and also without weighting. Principle component analysis was applied to these six features to ensure that they were not redundant with each other. This analysis resulted in three significant components having eigenvalues greater than 1, which explained 83% of the total variance among the six features. To reach about 95% of variance, a fourth component is required. These results demonstrate that the six weighting operators provide a rich diversity of feature data.

### Comparison of information content among sequence-based descriptors of ProtDCal, PROTEIN RECON and PROFEAT

The second and highly significant subject, which should be validated in any novel features generator program, is the degree of redundancy among its descriptors. That is, in order to be truly useful resource, the descriptors must encode intrinsically different information. The general paradigm is that features should be simultaneously relevant (i.e. each feature helps differentiate among proteins) and non-redundant (i.e. do not simply duplicate information encoded by other features).

In that sense, PCA was conducted in order to demonstrate the relations in content of information between PROFEAT, PROTEIN RECON and ProtDCal. Note that in this test, the quality measure is that features from a particular software shows significant loading (absolute values > 0.7) in a given component where no other software has substantial loadings.

To ensure we only consider relevant features, we took the top 30% most relevant descriptors of PROFEAT, PROTEIN RECON, and PROTCAL (using the Kier-Hall weighting function) according Shannon entropy test, (see Additional file 4). PCA was first applied to each feature set in isolation in order to obtain condensed representations of the encoded information of each software. Parameters were adjusted to extract enough components to describe 95% of the variance within the data. A total of 1 principal component was extracted from PROTEIN RECON, 111 from PROFEAT, and 125 from ProtDCal, which indicates the enormous redundancy among PROTEIN RECON’s descriptors. Next, all initial components from each software were assembled together and a second PCA followed by Varimax Normalized rotation was carried out. A total of 191 composed components were extracted in order to explain 95% of the data variance. Since PCA provides a list of components sorted by decreasing ability to explain the variance in the data, the highest ranking components tend to be the most important. Therefore, we were able to evaluate the distribution of the software-specific initial components among the final composed components. Additional file 5 summarizes the filtered rotated composed component matrix with the list of software-specific initial components and their loadings to every composed principal component (only variable loadings with absolute values greater than 0.7 are shown).

Additional file 1: Table SM-14 shows the results of the explained variance results of this PCA. First, the analysis showed that the three software packages have high loadings in the first, and more significant, component. Additionally, PROFEAT and ProtDCal share high loadings in the factor 2. However, an interesting behaviour is observed from factor 3 onwards: no other component is loaded by initial factors arising from both programs at the same time, which means that the information stored in those components is mostly divided, part in ProtDCal and part in PROFEAT. This analysis indicates that ProtDCal provides more useful features in that, of the 20 top ranked composed components, 16 (i.e. 80%) have high loadings from strictly ProtDCal initial components, and all of composed components 1–13 have high loadings from ProtDCal initial components. Taken together, this demonstrates that the components of ProtDCal are more relevant than those arising from PROFEAT. In total, ProtDCal achieves high loadings in 103 composed components, comprising 51.81% of explained variance, whereas PROFEAT loads 90 composed components, for a 45.30% of explained variance.

These results prove that in addition to the enormous capacity of descriptors generation of ProtDCal, this program has a low rate of redundancy among its most relevant descriptors. Furthermore, we have demonstrated that ProtDCal descriptors provide information not represented within the descriptors of PROFEAT and PROTEIN RECON.

Here is worth highlighting that the first experiment showed that the structure-based descriptors implemented in ProtDCal have different information than sequence-based descriptors, thus, this further increases the capability of ProtDCal to generate informative descriptors.

### Illustrative example: using ProtDCal’s features in the prediction of N-glycosylation sites

Glycosylation is one of the most common protein post-translational modifications (PTM) occurring in diverse organisms [54]. As consequence of this modification, a glycan is linked to the polypeptide chain; particularly, N-linked glycosylation modifies an asparagine residue. This type of PTM is closely associated with a sequence motif (sequon) defined as: Asn-Xxx-Thr/Ser, where Xxx can be any residue but proline. However, the existence of this sequon is not sufficient to dictate the occurrence of the glycosylation [55]. N-linked glycosylation is known to influence protein folding [56], cell-cell interactions, and antigenicity [57,58]. Therefore, the development of computational methods for predicting N-glycosylation sites within a protein sequence would facilitate protein functional annotation.

As demonstration that ProtDCal is able to automatically extract meaningful and information-rich features from protein sequence, we have created and evaluated N-linked glycosylation prediction systems using ProtDCal-generated features. Here, we compare the performance of models trained with ProtDCal’s features, and four contemporary predictors of N-glycosylation sites: GPP [59], NetNglyc (http://www.cbs.dtu.dk/services/NetNGlyc/), EnsembleGly [60] and ScanSite [61]. The performance metrics of these four methods were taken from the report of Hamby and Hirst, 2008 [59], using a dataset of 241 proteins obtained from OGLYCBASE [62]. This dataset was also used to train and evaluate the ProtDCal-based predictors.

A total number of 3508 sequence-unique windows (length = 15 AA) were extracted from the initial dataset, see Additional file 7, where each window was centered on an asparagine that was either known to be glycosylated (positive) or not (negative, i.e. assumed to be non-glycocylated). ProtDCal sequence-based features were computed for each position of these segments. Feature selection was carried out twice using the Weka wrapper approach: once using a Random Forest (RF) classifier as the evaluator, and once using a Naive Bayes (NB) classifier. Both feature selection searches were carried out using a genetic algorithm search of 500 generations and 50 chromosomes in each population. For the RF classifier, Weka’s default parameters were used, and for NB a supervised discretization of attribute values was applied to convert numeric features to nominal ones. Class imbalance was handled by resampling a reduced subset of instances in each training fold, in order to obtain balanced training subsets for each fold of the cross-validation. These searches of the feature space resulted on two datasets: one for NB containing four features, and another for RF comprising six features, see Additional file 6.

Comparison studies were conducted in the following way: First, the results, in 10-fold cross-validations, of the ProtDCal models, trained with RF and NB, were compared with the results of the predictor GPP using the original performance metrics reported by the authors [59], see Table 2. This comparison shows a slightly lower performance of ProtDCal models when using RF. On the other hand, when using NB, ProtDCal achieves a significantly superior sensitivity than GPP, maintaining the specificity over 90%. The global accuracy of ProtDCal_NB is also slightly higher than GPP. Such results are significant considering that GPP features were hand-selected leveraging domain-specific knowledge, while ProtDCal features were automatically extracted from the input sequence data with no domain-specific knowledge.

**Table 2:** **Performance metrics for N-linked glycosylation prediction using GPP and ProtDCal features using Random Forest and Naïve Bayes classifiers**

	***Random Forest***			***Naïve Bayes***		
	**CCI (%)**	**Sensitivity (%)**	**Specificity (%)**	**CCI (%)**	**Sensitivity (%)**	**Specificity (%)**
*GPP*	92.8	96.6	91.8	90.3	83.8	94.6
*ProtDCal*	91.6	93.2	91.4	91.1	97.6	90.6

CCI = Correctly classified instances. GPP results from Hamby & Hirst [59].

Next, GPP and ProtDCal’s models were compared with three other contemporary predictors, see Table 3. The values summarized in this table show that ProtDCal and GPP have better performance than NetNglyc and ScanSite, however EnsembleGly provides slightly improved sensitivity. Nonetheless, a direct comparison with the reported metrics of EnsembleGly should be considered with caution, since they were obtained based on a *sequence*-based 5-fold cross-validation approach, while the other methods use *window*-based 10-fold cross-validation. Importantly, in the former approach, highly similar sequence windows may appear in both the training and testing data leading to a potentially optimistically-biased performance metric.

**Table 3:** **Performance metrics for N-linked glycosylation prediction from different contemporary predictors**

	***ProtDCal_RF***	***ProtDCal _NB***	***GPP***	***NetNglyc***	*EnsembleGly	***Scan Site***
*CCI (%)*	91.6	91.1	92.8	76.7	95.0	79.8
*Sensitivity (%)*	93.2	97.6	96.6	43.9	98.0	72.7
*Specificity (%)*	91.4	90.6	91.8	95.7	77.0**	81.9

Results reproduced from Hamby & Hirst [59]. CCI = Correctly classified instances. *Metrics of EnsembleGly are based on sequences-based 5-fold cross-validation. **This value refers to precision [= TP/(TP + FP)] and not to specificity [= TN/(TN + FP)] as it was originally reported [60].

Ultimately, a blind test is conducted to measure the actual prediction capability of ProtDCal models and GPP. This was conducted to ensure that the cross-validation performance is sustained on new independent test data, not used for feature selection. The test is carried out using an external dataset extracted from dbPTM [63] (http://dbptm.mbc.nctu.edu.tw), which is a database compiling experimentally verified post-translational modifications of proteins, including glycosylation. A subset comprising 216 positive and 1918 negative sequence-unique windows were extracted from dbPTM, see Additional file 8, to form the final external dataset such that no test data shared sequence identity with the cross-validation dataset used above for feature selection. The final class imbalance is approximately 10 negatives for each positive, which is consistent with the original dbPTM dataset.

Results of this blind test are summarized in Table 4. The obtained performance validates the greater prediction capability of ProtDCal models given significantly higher values of accuracy, specificity and precision.

**Table 4:** **Performance metrics for N-linked glycosylation prediction from using GPP ProtDCal’s models in a blind test**

	***CCI (%)***	***Sensitivity (%)***	***Specificity (%)***	***Precision (%)***
*ProtDCal_RF*	87.11	93.50	86.40	43.60
*ProtDCal_NB*	86.78	95.80	85.80	43.10
*GPP*	66.21	97.22	62.72	22.70

CCI = Correctly classified instances.

In general, these analyses validate the applicability of ProtDCal’s features in obtaining models with predictive capabilities similar or better that state of art predictors of sites of N-glycosylation. Considering that no domain-specific knowledge was used to extract these features, it is expected that ProtDCal will be equally applicable to other fields.

### Computational complexity of ProtDCal

Finally, we conducted a simple experiment to study the computational cost of ProtDCal calculations. This program is computationally efficient and intended to be run on common desktop or even laptop computers. In order to analyze the runtime values of the main families of features related to the proteins size, we designed the following experiment:

First, to assure not-biased file-reading times due to extra lines in the PDB files, the used dataset of 876 proteins was cleaned by removing 31 PDB files containing either explicit hydrogen atoms or incomplete sets of atoms in several residues. Furthermore, all ANISOU lines were removed as well as REMARK lines, as these are irrelevant to the extraction of descriptors.

Five datasets of features were calculated and the runtimes were saved for each protein, including time required for reading the input file, calculation, and writing output. The selected features were: first, a total of 17986 features composed from all topographic indices (weighted just with the topological distance), second, a total of 7245 features resulting from the selection of all the structure-based thermodynamic indices; third, 1555 descriptors derived from all the sequence-based thermodynamic indices, and a fourth set of 5040 descriptors obtained by choosing all the property-based indices (TAE indices not included). For all these features families no weighting operator was applied and all the aggregation operators were selected. For the third and fourth sets of indices, all groups of residues were examined except those associated with structure (e.g. INT, SUP), to restrict the corresponding calculations to purely sequence-based features. A special fifth selection of 17204 topographic features was carried out by leaving aside one particular index: the *cliquishness* (aka *clustering coefficient*) [35]. This index was introduced by Micheletti (2003) in order to study the native topology influence on protein folding rate and transition state placement. Rather than strictly dealing with pairwise inter-residue contacts, this metric considers triads of residues to define the contacts. Figure 5 shows how the runtime dependence on protein length changes from a fairly linear behaviour to a quadratic trend because of the calculation of this index. Calculations were run on a laptop computer with processor Intel Core i5-3210 M 2.5 GHz (6GB RAM total; 64 MB assigned to JVM).

The obtained runtimes for each protein were divided by the number of computed features in each dataset in order to estimate an average runtime per protein per feature. Results of these experiments are given in Figure 5.

Additional file 1: Table SM-15 summarizes the runtime of every protein used in the experiment. The obtained results demonstrate the fast execution time of ProtDCal, with observed runtimes on the order of 10^−4^ seconds per protein per feature, and showing linear variation within proteins lengths from 50 to 500 residues. Nonetheless, to facilitate batch-mode calculations, *project files* may be saved directly from the GUI. These files store information related to the path of the data directory, the selected indices, weighting operators, groups, aggregation operators, and parameters needed for the calculation. Several of these project files can be loaded, in batch mode, by using the *multi-projects* menu in the GUI.

## Conclusions

The summary of the analyses presented in this manuscript validates the capabilities of ProtDCal to generate valuable sequence- and structure-based protein descriptors. ProtDCal may provide to the protein data mining community a free, portable, and computationally efficient tool to generate a wide variety of meaningful descriptors for protein sequences and structures. We have demonstrated that ProtDCal sequence-based descriptors provide more relevant and low redundant information than what is currently available through sequence-based feature generation servers. In addition, we have shown that structure-based descriptors contribute significant additional information to that encoded by sequence-based ones. These latter descriptors are expected to enhance the quality of protein structure-function studies based on the ever-increasing availability of structural models from experimental and computational predictions [4]. The use of different metrics of distance, central tendency, and dispersion over groups of residues, constitute a modern and successful approach to encode relevant structural information as discussed by some of the authors previously. Ultimately, as a demonstration of the utility of ProtDCal feature data, N-glycosylation site prediction models were trained using these data. Classification performance of the obtained models, compare favourably with contemporary predictors, which leverage domain-specific knowledge. Considering its significant protein encoding capacity, ProtDCal enriches the feature-based representation of proteins, becoming a potentially valuable contribution the state of art of a wide range of applications in proteomics.

### Future outlook

We expect that ProtDCal will become an alignment-free protein-modelling platform to generate relevant features for protein sequences and/or structures. Future developments will allow ProtDCal to compute, select, and assess features within an integrated analysis pipeline, by combining the feature generation with attribute selection strategies as implemented in libraries of the Weka software package.

## Availability and requirements

**Project name:** ProtDCal, see Additional file 9 for a tutorial guide.

**Project home page:** http://bioinf.sce.carleton.ca/ProtDCal

**Operating system(s):** Platform independent

**Programming language:** Java

**Other requirements:** JDK-7 or higher

**License:** GNU GPL

## Additional files

Additional file 1: Table SM-1. Formulae and description of 3D-Thermodynamics Indices. **Table SM-2.** Formulae and description of Thermodynamics Indices for Protein Sequences. **Table SM-3.** Formulae and description of Topographic Indices. **Table SM-4.** Compendium of structural and chemical-physical aminoacid properties. **Table SM-5.** Models implemented in the *Thermo&kinetics* menu of ProtDCal. **Table SM-6.** Aggregation operators: Norms (Metrics) Invariants. **Table SM-7.** Aggregation operators: Mean (First Statistical Moment) Invariants. **Table SM-8.** Aggregation operators: Statistical (Highest Statistical Moments) Invariants. **Table SM-9.** Aggregation operators: Information-Theory-based Invariants. **Table SM-10.** Weighting operators (*Windex*) implemented in ProtDCal. **Table SM-11.** Summary of the definitions of property-based groups. **Table SM-12.** List of PDB codes and sequence length of the proteins used for features analyses. **Table SM-13.** Explained variance results for the first 159 components of the PCA carried out with 3D and sequence-based protein descriptors. **Table SM-14.** Explained variance of the PCA carried out with extracted components of PROFEAT, PROTEIN RECON and ProtDCal. **Table SM-15.** Runtime values per descriptor per protein for different families of features.
Additional file 2:
**Summary of TAE (Transferable Atom Equivalent) indices.**

Additional file 3:
**Rotated component matrix of the PCA carried out with sequence- and structure-based descriptors.** Only the entries whose absolute values are larger that 0.7 are shown.
Additional file 4:
**Summary of the Shannon entropy ranks for different families of sequence-based descriptors. **

Additional file 5:
**Rotated component matrix of the PCA carried out with the factors extracted from ProtDCal, PROFEAT and PROTEIN RECON.** Only the entries whose absolute values are larger that 0.7 are shown.
Additional file 6:
**Training and test sets used with the techniques: Naive Bayes and Random Forest, for the study of N-linked glycosylation sites.**

Additional file 7:
**List of sequence windows in the training and test sets used for the study of N-linked glycosylation.**

Additional file 8:
**Weka's model files built with Naive Bayes and Random Forest, using ProtDCal's descriptors, to predict N-linked glycosylation sites.**

Additional file 9:
**Tutorial guide of how to use ProtDCal program as well as a practical example of how to train a model using ProtDCal's features and Weka.**
