# Supplementary material for: ProtDCal: A program to compute general-purpose-numerical descriptors for sequences and 3D-structures of proteins
Source: BMC Bioinformatics. 2015 May 16;16:162. doi: 10.1186/s12859-015-0586-0 (PMC4432771; doi:10.1186/s12859-015-0586-0)
Supplement: Additional file 1: Table SM-1. — Formulae and description of 3D-Thermodynamics Indices. Table SM-2. Formulae and description of Thermodynamics Indices for Protein Sequences. Table SM-3. Formulae and description of Topographic Indices. Table SM-4. Compendium of structural and chemical-physical aminoacid properties. Table SM-5. Models implemented in the Thermo&kinetics menu of ProtDCal. Table SM-6. Aggregation operators: Norms (Metrics) Invariants. Table SM-7. Aggregation operators: Mean (First Statistical Moment) Invariants. Table SM-8. Aggregation operators: Statistical (Highest Statistical Moments) Invariants. Table SM-9. Aggregation operators: Information-Theory-based Invariants. Table SM-10. Weighting operators (Windex) implemented in ProtDCal. Table SM-11. Summary of the definitions of property-based groups. Table SM-12. List of PDB codes and sequence length of the proteins used for features analyses. Table SM-13. Explained variance results for the first 159 components of the PCA carried out with 3D and sequence-based protein descriptors. Table SM-14. Explained variance of the PCA carried out with extracted components of PROFEAT, PROTEIN RECON and ProtDCal. Table SM-15. Runtime values per descriptor per protein for different families of features. [file 12859_2015_586_MOESM1_ESM.pdf]

PROTDCAL: A Program to Compute General-Purpose Numerical Descriptors for Sequences and 3D-Structures of Proteins

Yasser B. Ruiz-Blanco,[1*] Waldo Paz,[1,2] James Green[4] and Yovani Marrero-Ponce[1,3]

Supplementary Information

Table of Content:

Table SM-1. Formulae and description of 3D-Thermodynamics Indices.
Table SM-2. Formulae and description of Thermodynamics Indices for Protein Sequences.
Table SM-3. Formulae and description of Topographic Indices.
Table SM-4. Compendium of structural and chemical-physical aminoacid properties.
Table SM-5. Models implemented in the *Thermo&kinetics* menu of PROTDCAL.
Table SM-6. Aggregation operators: Norms (Metrics) Invariants.
Table SM-7. Aggregation operators: Mean (First Statistical Moment) Invariants.
Table SM-8. Aggregation operators: Statistical (Highest Statistical Moments) Invariants.
Table SM-9. Aggregation operators: Information-Theory-based Invariants.
Table SM-10. Weighting operators (*Windex*) implemented in PROTDCAL.
Table SM-11. Summary of the definitions of property-based groups.
Table SM-12. List of PDB codes and sequence length of the proteins used for features analyses.
Table SM-13. Explained variance results for the first 159 components of the PCA carried out with 3D and sequence-based protein descriptors.
Table SM-14. Explained variance of the PCA carried out with extracted components of PROFEAT, PROTEIN RECON and PROTDCAL.
Table SM-15. Runtime values per descriptor per protein for different families of features.

Table SM-1. Formulae and description of 3D-Thermodynamics Indices.

Acronym	Formula	Description
$Gc_{(F)}$	$G_c(F)_i = RT(N-1)p_i \ln p_i$,<br>$p_i = \left(\frac{3}{2\pi(i-1)3.8^2}\right)^{3/2} e^{-\frac{3r_i^2}{2(i-1)3.8^2}}$	Configurational free energy of a folded state. Index based on a "random-flight" model of the protein chain. (1)<br>Where r_i represents the distance to the first residue in the chain.
$W_{(F)}$	$W_i^F = \sum_{j=1}^{N} \delta_{ij}^{ng} \delta_j^{s} N_j^{w}$	Number of water molecules close to a residue in a folded state.(2,3)<br>Where δ^{ng} takes value 1 if the pair of residues are neighbours, using a cutoff for the spatial distance (9.4 Å), or 0 otherwise. In the same way δ^{s} takes value 1 if the residue is superficial, using a cutoff for the solvent accessible surface area, or 0 otherwise. The parameters N^{w} represents the number of associated water molecules to the sidechain of a residue (4).
$Gw_{(F)}$	$G_w(F)_i = -TR\delta_{hyd} \ln \frac{W_i^F!}{(W_i^F - N_i^w)!}$	Free energy contribution of the entropy of the first shell of water molecules in a folded state (3). δ_{hyd} takes value 1 if the residue has non-zero N_i^w, or zero otherwise.
$Gs_{(F)}$	$G_s(F)_i = H_i A_i^F$	Interfacial free energy contribution of a folded state.<br>Where H_i is hydrophobicity in Kyte-Doolittle scale (5) and A^F is the solvent accessible surface area of a residue in a folded state.
ΔG_s	$\Delta G_{s\,i} = G_s(F)_i - G_s(U)_i$	Interfacial free energy variation.
HBd	$\Delta Hbd_i = 0.5\sum_{j=1}^{N}(\delta_{ij}^{N} + \delta_{ij}^{O})$<br>Geometric definition of a H-bond:<br>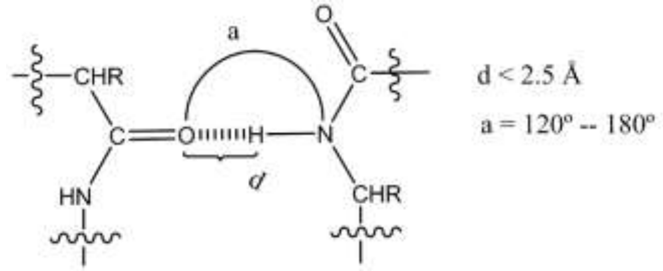 	Number of backbone's hydrogen bonds.<br>Where δ_{ij}^{N} takes value 1 if the Nitrogen atom of residue *i* is H-bonded with the Oxygen atom of residue *j* and 0 otherwise. In the same way δ_{ij}^{O} takes value one if the Oxygen atom of residue *i* is H-bonded with the Nitrogen atom of residue *j* and zero otherwise.
ΔG_{el}		Free energy contribution of the

	$$\Delta G_{el\,i} = -\frac{\mathrm{k_{el}}}{2r^2}\sum_{j=1}^{N}\frac{q_i q_j r_i r_j}{r_{ij}}$$	charge distribution within the protein. The parameters q are the Electronic Charge Indices of each residue (6). Parameter k_{el}= 7.608.
ΔG_w	$$\Delta G_{w\,i} = k_w(G_w(F)_i - G_w(U)_i)$$	Folding free energy contribution of the entropy of the first shell of water molecules.
ΔG_{LJ}	$$\Delta G_{LJ\,i} = \frac{\mathrm{k_{LJ}}}{2}\sum_{\substack{j=1;\\ \lvert j-i\rvert>1}}^{N}\left[\left(\frac{3.965}{r_{ij}}\right)^{12} - \left(\frac{3.965}{r_{ij}}\right)^{6}\right]$$	Residue-level Lennard-Jones interactions. Parameter k_{LJ} = 63.981.
ΔG_{tor}	$$\Delta G_{tor\,i} = k_{tor}[(\cos^2 2\phi_i - 1) + 0.256(\cos^2 2\psi_i - 1)]$$	Free energy contribution of backbone torsion angles. Parameter k_{tor}= 1.219.

Table SM-2. Formulae and description of Thermodynamics Indices for Protein Sequences.

Acronym	Formula	Description
W(U)	$W_i^U = \sum_{j=i-2}^{i+2} N_j^w$	Number of water molecules close to a residue in an unfolded state (3).
Gw(U)	$G_w(U)_i = -TR\delta_{hyd} \ln \frac{W_i^U!}{(W_i^U - N_i^w)!}$	Free energy contribution from the entropy of the first shell of water molecules in an unfolded state (3).
Gs(U)	$G_s(U)_i = H_i A_i^U$	Interfacial free energy contribution of an unfolded state

Table SM-3. Formulae and description of Topographic Indices.

**Acronym**	**Formula**	**Description**
A_F	-	Solvent accessible surface area
ΔA	$\Delta A = A_F - A_U$	Buried area. Where A_U is the fully exposed surface area of each residue and A_F is the area in the folded state.
ΔA^{np}	$\Delta A^{np} = A^{np}_F - A^{np}_U$	Buried non-polar area. Here nitrogen atoms and oxygen atoms are excluded.
wSp	$wSp_i = \omega_i * \delta_i^s$	Weighted index of the solvent accessibility. Where ω represents any weighting property and the delta takes value 1 or 0 if the residue is considered superficial or internal respectively.
lnFD	$\ln FD_i = -\frac{\sum_{j;\lvert j-i\rvert>1}^{N} \lvert j-i\rvert / d_{ij}^3}{N-x}$	Logarithm of the Folding Degree. Where *d* is the spatial distance, *N* the length of the protein and *x* a parameter which takes value 2 for terminal residues and 3 for the others.
wR^2	$wRG_i^2 = \frac{w_i * d_i^2}{\sum_{i=1}^{N} w_i}$	Weighted Squared Radius. Where ω represents any weighting property and *d* is the spatial distance.
wΔHBd	$\Delta Hbd_i = \omega_i * (\delta_N + \delta_O)$ Geometric definition of a H-bond: 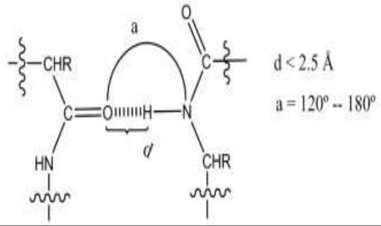	Weighted deficit or excess of the H-bond between the backbone atoms. Where δ_{ij}^N takes value 1 if the nitrogen atom of residue *i* is buried ($A_{(N)}$ < 0.01Å) and is not H-bonded with any oxygen atom or 0 otherwise. In the same way δ_{ij}^O takes value 1 if the oxygen atom of residue *i* is buried ($A_{(O)}$ < 0.01Å) and is not H-bonded with any nitrogen atom and 0 otherwise.
wNc	$wNc_i = 0.5\sum_{j\neq i}^{N} \omega_{ij}\delta_{ij}^c$	Weighted Number of Contact. Where δ_{ij}, takes value 1 when the contact conditions are fulfilled and 0 otherwise. A contacts is defined for pair of residues with spatial distances shorter than a cutoff *d* and topological distances longer than a cutoff *t*. The parameter ω_{ij} represents a weighting coefficient for each pair of residues. This parameter is computed as the product, $\omega_i\omega_j$, of the values, for each residue, of any property within a pool of 12 aminoacid properties covering structural, physical-chemical features.
wFLC	$wFLC_i = \frac{\sum_{\lvert j-i\rvert\le 4}^{N} \omega_{ij}\delta_{ij}^c}{\sum_{i=1}^{N}\sum_{j=1}^{N} \omega_{ij}\delta_{ij}^c}$	Weighted Fraction of Local Contancts. The parameters δ_{ij} and ω_{ij} means the same as previous but here the topological cutoff value is fixed in *t* = 1.
wNLC	$wNLC_i = 0.5 \sum_{\lvert j-i\rvert\le 4}^{N} \omega_{ij}\delta_{ij}^c$	Weighted Number of Local Contact The parameters δ_{ij} and ω_{ij} means the same as in *wNc* but here the topological cutoff value is fixed in *t* = 1.
wCO	$wCO_i = \frac{1}{2NN_c}\sum_{j\neq i}^{N} \omega_{ij}\delta_{ij}^c$	Weighted Relative Contact Order (7). Where Nc represents the number of contacts in the protein.

wLCO	$wLCO_i = \frac{\sum_{j\neq i}^{N} \omega_{ij}\delta_{ij}^{c}}{N\sum_{j\neq i}^{N} \delta_{ij}^{c}}$	Weighted Local Contact Order. As difference with previous, the weighted contacts are divided by the same un-weighted local contact instead of all the contact in the protein.
wRWCO	$wRWCO_i = \frac{\sum_{j\neq i}^{N} \omega_{ij}\delta_{ij}^{c}}{N}$	Weighted Residue-Wise Contact Order (8).
wCTP	$wCTP_i = \frac{1}{2NN_c}\sum_{j\neq i}^{N} \omega_{ij}^{2}\delta_{ij}^{c}$	Weighted Chain Topology Parameter (9).
wCLQ	$wCLQ_i = \frac{\sum_{j<l} \delta_{ij}\delta_{il}\delta_{lj}\omega_{ij}\omega_{il}\omega_{lj}}{\sum_{j<l} \delta_{ij}\delta_{il}\omega_{ij}\omega_{il}}$	Weighted Cliquishness or Clustering Coefficient (10).
wPsi_H	$Psi\_H_i = \delta_i^{\psi H} * \omega_i$	Weighted Helix-like Psi angle.<br>The delta takes value 1 if the angle is in the range [-77;-17] or 0 otherwise.
wPsi_S	$Psi\_S_i = \delta_i^{\psi S} * \omega_i$	Weighted Sheet-like Psi angle. The delta takes value 1 if the angle is in the range [94;154] or 0 otherwise.
wPsi_I	$Psi\_I_i = \delta_i^{\psi I} * \omega_i$	Weighted Irregular Psi angle. The delta takes value 1 if the angle is in one of the following ranges: [-180,-77), (-17;94), (154;180] or 0 otherwise.
wPhi_H	$Phi\_H_i = \delta_i^{\phi H} * \omega_i$	Weighted Helix like Phi angle. The delta takes value 1 if the angle is in the range [-87;-27] or 0 otherwise.
wPhi_S	$Phi\_S_i = \delta_i^{\phi S} * \omega_i$	Weighted Sheet like Phi angle. The delta takes value 1 if the angle is in the range [-159;-99] or 0 otherwise.
wPhi_I	$Phi\_I_i = \delta_i^{\phi I} * \omega_i$	Weighted Irregular Phi angle. The delta takes value 1 if the angle is in one of the following ranges: [-180,-159), (-99;-87), (-27;180] or 0 otherwise.
Phi	-	Phi diedral angle
Psi	-	Psi diedral angle
TCD	$wTCD_i = \frac{1}{2N^2}\sum_{j\neq i}^{N} \omega_{ij}\delta_{ij}^{c}$	Total Contact Distance (11).

Table SM-4. Compendium of structural and chemical-physical aminoacid properties.

	Mw	HP	IP	ECI	Vm	Z1	Z2	Z3	ISA	At	Pa	Pb	Pt
**ALA**	71	1.8	6.01	0.05	88.6	0.07	-1.73	0.09	62.9	281.103	1.29	0.9	0.78
**ARG**	156	-4.5	10.76	1.69	173.4	2.88	2.52	-3.44	52.98	642.81	0.96	0.99	0.88
**ASN**	114	-3.5	5.41	1.31	114.1	3.22	1.45	0.84	17.87	424.35	0.9	0.76	1.28
**ASP**	115	-3.5	2.77	1.25	111.1	3.64	1.13	2.36	18.46	414.11	1.04	0.72	1.41
**CYS**	103	2.5	5.07	0.15	108.5	0.71	-0.97	4.13	78.51	349.851	1.11	0.74	0.8
**GLN**	128	-3.5	3.22	1.31	143.8	3.08	0.39	-0.07	19.53	510.436	1.44	0.75	1
**GLU**	129	-3.5	5.65	1.36	138.4	2.18	0.53	-1.14	30.19	496.493	1.27	0.8	0.97
**GLY**	57	-0.4	5.97	0.02	60.1	2.23	-5.36	0.3	19.93	216.465	0.56	0.92	1.64
**HIS**	137	-3.2	7.59	0.56	153.2	2.41	1.74	1.11	87.38	531.47	1.22	1.08	0.69
**ILE**	113	4.5	6.02	0.09	166.7	-4.44	-1.68	-1.03	149.77	461.549	0.97	1.45	0.51
**LEU**	113	3.8	5.98	0.01	166.7	-4.19	-1.03	-0.98	154.35	474.874	1.3	1.02	0.59
**LYS**	128	-3.9	9.74	0.53	168.6	2.84	1.41	-3.14	102.78	537.414	1.23	0.77	0.96
**MET**	131	1.9	5.74	0.34	162.9	-2.49	-0.27	-0.41	132.22	513.692	1.47	0.97	0.39
**PHE**	147	2.8	5.48	0.14	189.9	-4.92	1.3	0.45	189.42	595.661	1.07	1.32	0.58
**PRO**	97	-1.6	6.48	0.16	112.7	-1.22	0.88	2.23	122.35	369.134	0.52	0.64	1.91
**SER**	87	-0.8	5.68	0.56	89	1.96	-1.63	0.57	19.75	322.375	0.82	0.95	1.33
**THR**	101	-0.7	5.87	0.65	116.1	0.92	-2.09	-1.4	59.44	376.294	0.82	1.21	1.03
**TRP**	186	-0.9	5.89	1.08	227.8	-4.75	3.65	0.85	179.16	718.156	0.99	1.14	0.75
**TYR**	163	-1.3	5.66	0.72	193.6	-1.39	2.32	0.01	132.16	630.633	0.72	1.25	1.05
**VAL**	99	4.2	5.97	0.07	140	-2.69	-2.53	-1.29	120.91	400.581	0.91	1.49	0.47

**Mw**	Molar Weight (12)
**HP**	Kyte-Doolitle's Hydrophobicity Scale (5)
**IP**	Isoelectric Point (12)
**ECI**	Electronic Charge Index (6)
**Vm**	Aminoacid volume (Å^3) (13)
**Z1**	Composed parameter related with hydrophilicity (14)
**Z2**	Composed parameter related with steric features (14)
**Z3**	Composed parameter related with electronic features (14)
**ISA**	Isotropic Surface Area (6)
**At**	Estimated solvent accessible surface area of residues in fully exposed states
**Pa**	Levitt's Probability of adopting alpha helix conformation (15)
**Pb**	Levitt's Probability of adopting beta sheet conformation (15)
**Pt**	Levitt's Probability of adopting beta turn conformation (15)

Table SM-5. Models implemented in the *Thermo&kinetics* menu of PROTDCAL.

Acronym	Formula	Description
ΔGconf	$\Delta G_{conf} \approx -RT\ln 2^{-3\Delta N} = 2.08RT\Delta N$<br>$\Delta N = \sum_{i=1}^{N-2}\left(\frac{N-i}{N-1} * \frac{\sum_{j=i+2}^{N}\delta_{ij}\mid j-i\mid}{\sum_{j=i+2}^{N}\mid j-i\mid}\right) + \sum_{i=3}^{N}\left(\frac{i-1}{N-1} * \frac{\sum_{j=1}^{i-2}\delta_{ij}\mid j-i\mid}{\sum_{j=1}^{j-2}\mid j-i\mid}\right)$<br>$\delta_{ij} = \begin{cases} 1 & if \quad \frac{\mid j-i\mid}{d_{ij}} \geq \frac{4}{6.4} \\ 0 & else \end{cases}$	Folding configurational free energy (3).
ΔGHBd	$\Delta G_{Hbd} = \Delta G_{Hbd}^{N} + \Delta G_{Hbd}^{O}$<br>$\Delta G_{Hbd}^{N} = \sum_{i=1}^{N} 2.929\delta_i^N$<br>$\Delta G_{Hbd}^{O} = \sum_{i=1}^{N} 11.715\delta_i^O$<br>Geometric definition of a H-bond:<br>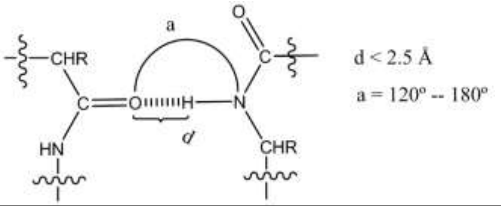 	Free energy penalty of unbalanced H-bond. Where δ_{ij}^{N} takes value 1 if an internal ($A_{(N)}$ < 0.01Å) Nitrogen atom is H-bonded with an Oxygen atom and 0 otherwise. In the same way δ_{ij}^{O} takes value one if an internal ($A_{(O)}$ < 0.01Å) Oxygen atom is H-bonded with a Nitrogen atom and 0 otherwise.
ΔGcpack	$\Delta G_{cpack} = \Delta G_{el} + \Delta G_{LJ} + \Delta G_{HBd} + \Delta G_{tor}$	Folding free energy of close-packing interactions. See thermodynamic indices for definitions of each term.
ΔGwat	$\Delta G_{wat} = k_{np}^{w}\Delta A_{np} - RTk_{p}^{w}\sum_{i=1}^{N_{hyd}}\delta_i^S\gamma_i$<br>Where<br>$\gamma_i = \ln\left[\frac{\left(\frac{g_i^F}{N_i^w}\right)}{\left(\frac{g_i^U}{N_i^w}\right)}\right]$ and<br>$g_i = N_i^W + \sum_{j=1}^{N}\delta_{ij}^{nb}\delta_j^S N_j^w$	Folding free energy associated with hydrophobic effect (3). The parameter N_i^w represents the number of water molecules associated to each residue (4). Parameters k_{np} = 0.054262 and k_p = 0.162562. The parameter N_{hyd} represents the number of residues with non-zero N_i^w
ΔGscr	$\Delta G_{scr} = \Delta G_{wat} + \omega(\Delta G_{el} + \Delta G_{LJ} + \Delta G_{tor} + \Delta G_{HBd})$	Scoring potential for the discrimination of folded

		structures. Parameter ω = 0.01024
ΔG_f	$\Delta G_f = \omega_1(\Delta G_{wat} + \Delta G_{conf}) + \omega_2(\Delta G_{el} + \Delta G_{LJ} + \Delta G_{tor} + \Delta G_{HBd})$	Folding free energy potential. Parameters ω_1 = -0.08016 and ω_2 = 0.01024.
$\ln k_f$	$\ln(k_f) = \varepsilon_1 \ln(\Delta G_{cpack} + \Delta G_{conf}) + \varepsilon_2 \ln L_0 - \varepsilon_3 \ln F\mathrm{D} + \varepsilon_4$	Logarithms of the folding rate constant. The parameters ε_1 = 6.76, ε_2 = -13.31, ε_3 = -416.92 and ε_4 = 29.67

Table SM-6. Aggregation operators: Norms (Metrics) Invariants.

**Acronym**	**Formula**	**Description**
N1	$N1=\sum_{i=1}^{N}\lvert L_i\rvert$	Minkowski's norms (p = 1) Manhattan norm. Where L_i represents each index of the group of indices and N the number of indices in the group.
N2	$N2=\sqrt{\sum_{i=1}^{N}\lvert L_i\rvert^2}$	Minkowski's norms (p = 2) Euclidean norm. Where L_i represents each index of the group of indices and N the number of indices in the group.
N3	$N3=\sqrt[3]{\sum_{i=1}^{N}\lvert L_i\rvert^3}$	Minkowski's norms (p = 3). Where L_i represents each index of the group of indices and N the number of indices in the group.

Table SM-7. Aggregation operators: Mean (First Statistical Moment) Invariants.

Acronym	Formula	Description
G	$G = \sqrt[N]{\prod_{i=1}^{N} L_i}$	Geometric Mean.<br>Where N is the number of indices in the group.
M	$m_\alpha = \left(\frac{L_1^\alpha + L_2^\alpha + \ldots + L_N^\alpha}{N} \right)^{\frac{1}{\alpha}}$	Arithmetic Mean<br>(potencial with α = 1)
P2		Quadratic Mean<br>(potencial with α = 2)
P3		Potential Mean<br>(potencial with α = 3)
A		Harmonic Mean<br>(potential with α = -1)

Table SM-8. Aggregation operators: Statistical (Highest Statistical Moments) Invariants.

Acronym	Formula	Description
V	$V = \frac{\sum_{i=1}^{N}\left(L_i - \overline{L}\right)^2}{N - 1}$	Variance.<br>Where N is the number of indices in the group.
S	$S = \frac{N(X_3)}{(N-1)(N-2)(DE)^3}$<br>$X_3 = \sum_{a=1}^{N}(L_a - \overline{L})^3$	Skewness.<br>Where N is the number of indices in the group and $(DE)^3$ is the standard deviation raised to the 3rd power
K	$k = \frac{N(N+1)X_4 - 3(X_2)(X_2)(N-1)}{(N-1)(N-2)(N-3)(DE)^4}$<br>$X_j = \sum_{a=1}^{N}(L_a - \overline{L})^j$	Kurtosis.<br>Where $(DE)^4$ is the standard deviation raised to the fourth power
DE	$DE = \sqrt{\frac{\left(\sum L_i - \overline{L}\right)^2}{N-1}}$	Standard Deviation
CV	$c_v = S/\overline{L}$	Variation Coefficient
RA	$RA = L_{\max} - L_{\min}$	Range
Q1	$P25 = \left[\frac{N}{4} + \frac{1}{2}\right]$	Percentile 25.<br>Where N is the number of indices in the group.
Q2	$P50 = \left[\frac{N}{2} + \frac{1}{2}\right]$	Percentile 50.<br>Where N is the number of indices in the group.
Q3	$P75 = \left[\frac{3N}{4} + \frac{1}{2}\right]$	Percentile 75.<br>Where N is the number of indices in the group.
I50	$I50 = P75 - P25$	Inter-quartileRange
MX	L_i maximum	Maximum value of the group of indices.
MN	L_j minimum	Minimum value of the group of indices.

Table SM-9. Aggregation operators: Information-Theory-based Invariants.

Acronym	Formula (Equation)	Description
MI	$MI = -\sum_{i=1}^{K} \frac{N_k}{N} \log_2 \frac{N_k}{N}$	Mean Information Content.<br>Where N_k is the number of indices in the same bin, K is the number of bins defined to compute the operator and N is the total number of indices in the group.
TI	$TI = N \log_2 N - \sum_{k=1}^{K} N_k \log_2 N_k$	Total Information Content.
SI	$SI = \frac{TI}{N \log_2 N}$	Standarized Infomation Content

Table SM-10. Weighting operators (*Windex*) implemented in PROTDCAL.

Acronym	Formula	Description
AC_i^k	$AC_i^k = \sum_{j\geq 1}^{N} L_i L_j \delta(d_{ij}, k)$ $Condition: (d_{ij} = k)\,?\,\delta = 1 : \delta = 0$	Autocorrelation.<br>Where, L_x are the index values of residues *i* and *j* and *k* is a topological distance cutoff and *N* is the total number of residues.
GV_i^k	$GV_i^k = \frac{1}{N} \sum_{j=1; j\neq i}^{N} \frac{L_i L_j \delta(d_{ij}, k)}{d_{ij}}$	Gravitational
KH_i^m	$KH_i^m = \sum_{\alpha=1}^{A} \sqrt{\prod_{j=1}^{n_\alpha} L_{j\alpha}}$	Kier-Hall's connectivity-based operator.<br>Where, *A* is the number of segments containing the residue *i*, with a maximum length of *m* residues, n_α is the number of residues in a sub-segment, $L_{j\alpha}$ is the index value of the residue *j* in the segment *α*.
ES_i	$ES_i = L_i + \Delta L_i = L_i + \sum_{j=1; j\neq i}^{N} \frac{L_i - L_j}{(d_{ij}+1)^2}$	Electrotopological state (E-state index).<br>Where, L_i is the intrinsic state (index) of the ith residue and ΔL_i is the field effect on the *ith* residue calculated as perturbation of the index value (L_i) of ith residue by all other residues in the protein, d_{ij} is the topological distance between the ith and the jth residue, and N is the total number of residues.
IB_i^2	$IB_i^2 = (N-1)\sum_{j\neq i}^{N} a_{ij} \left(S_i S_j\right)^{-1/2}$ $S_i = L_i + \sum_{j\neq i}^{N} a_{ij} L_j$	Ivanciuc-Balaban.<br>Where, a_{ij} represents th elements of the adjacency matrix, and *N* is the number of residues. The exponent 2 dues to the use of the exponent -1/2. Here the factor (N-1) represents the numbers of virtual bonds among residues.

Table SM-11. Summary of the definitions of property-based groups.

Acronym	Included Residues	Description
**AHR**	ALA, CYS, GLN, GLU, HIS, LEU, LYS, MET	Common residues in alpha helix motifs.
**BSR**	ILE, PHE, THR, TRP, TYR, VAL	Common residues in beta sheet motifs.
**RTR**	ASN, ASP, GLY, PRO, SER	Common residues in reverse turn motifs.
**PCR**	ARG, HIS, LYS	Positive-electric-charged residues.
**NCR**	ASP, GLU	Negative-electric-charged residues.
**UCR**	ASN, CYS, GLN, SER, THR, TYR	Uncharged residues.
**ARM**	HIS, PHE, TRP, TYR	Aromatic residues.
**ALR**	ALA, GLY, ILE, LEU, MET, PRO, VAL	Aliphatic residues.
**UFR**	GLY, PRO	Common residues promoting unfolding or distorted regions.
**NPR**	ALA, GLY, ILE, LEU, MET, PHE, PRO, TRP, VAL	Non-polar residues.
**PLR**	ARG, ASN, ASP, CYS, GLN, GLU, HIS, LYS, SER, THR, TYR	Polar residues.

Table SM-12. List of PDB codes and sequence length of the proteins used for features analyses.

Protein	Length		Protein	Length		Protein	Length		Protein	Length
**1yzm**	46		**2RH3**	121		**2X5Y**	171		**4MQM**	269
**3I8Z**	50		**3SUL**	121		**3RT2**	171		**2XJ4**	270
**3ca7**	50		**1l6p**	121		**1qzn**	171		**3M66**	270
**2gkt**	51		**2grc**	121		**1wba**	171		**1oi7**	270
**2ERW**	53		**2qvk**	121		**1koe**	172		**2h7o**	270
**4G3O**	53		**3csp**	121		**1lki**	172		**2EGU**	271
**2CMP**	56		**2GKG**	122		**3PM2**	173		**3PWZ**	271
**1YP5**	58		**3T3K**	122		**1h4a**	173		**2VFB**	272
**1zlm**	58		**1lwb**	122		**2fcb**	173		**3H04**	272
**3cqt**	58		**1nko**	122		**1NG2**	176		**2qol**	273
**4pti**	58		**1pzc**	122		**2ZTY**	176		**1A8Q**	274
**1mhn**	59		**1zlb**	122		**4ANN**	176		**3SIH**	274
**1orc**	59		**2fwg**	122		**1sqw**	176		**3W9U**	274
**2fjz**	59		**3P6J**	123		**3FN7**	177		**1gqz**	274
**1tud**	60		**3RNV**	123		**3KSN**	177		**2ci3**	274
**2IGD**	61		**4IPC**	123		**1hbq**	177		**2plc**	274
**2PST**	61		**1c44**	123		**1yw5**	177		**2UYO**	275
**1i2t**	61		**1huf**	123		**2f1s**	177		**3ZXY**	275
**1ku3**	61		**2ciu**	123		**1CDY**	178		**3BZG**	278
**1l2p**	61		**2FC3**	124		**2OIX**	178		**1r3f**	278
**2CJJ**	63		**4DFI**	124		**1mjs**	178		**3F7M**	279
**1f94**	63		**1kf5**	124		**2i5h**	178		**3TEF**	279
**1r69**	63		**2F6E**	125		**2nx2**	178		**3eg4**	279
**2p5k**	63		**2X35**	125		**2hp7**	179		**1jks**	280
**1AHO**	64		**4B9I**	125		**2iu1**	179		**4HJP**	281
**1ucs**	64		**1acf**	125		**3d2a**	179		**2boe**	281
**1uoy**	64		**1bfg**	126		**1KN3**	180		**1SR8**	282
**1ypc**	64		**1tp6**	126		**3ICH**	180		**3RGI**	282
**2HDZ**	66		**1f32**	127		**3UC9**	180		**1f00**	282
**2OCH**	66		**1jb3**	127		**3cou**	180		**2HE7**	283
**3IDW**	66		**3A7L**	128		**1YTQ**	181		**2RIK**	283
**1tg0**	66		**3O5E**	128		**3EIZ**	182		**1cnv**	283
**3RDY**	67		**3O5P**	128		**3VOR**	182		**2BV9**	284
**4HCS**	67		**2W0G**	129		**4LEU**	182		**2CWC**	284
**1yu5**	67		**3NE3**	129		**4N30**	182		**1ltu**	284
**2hlr**	67		**3O48**	129		**1m1h**	182		**2zco**	284
**1xak**	68		**1gp3**	129		**2FL7**	183		**2OPW**	286
**2fht**	68		**1j3a**	129		**3UV9**	183		**1u6d**	288
**1mjc**	69		**1lsy**	129		**2P8T**	184		**4M9P**	289

**1pgx**	70		**2D4P**	130		**3KG4**	184		**1nar**	289
**3VDJ**	71		**2nwd**	130		**2E8B**	185		**1mtz**	290
**3ADG**	72		**3HD4**	131		**2YWJ**	185		**1bn6**	291
**3UCI**	72		**3NPH**	131		**3FZE**	185		**1h6t**	291
**2vc8**	72		**1lit**	131		**4JG2**	185		**1ks9**	291
**3ZHI**	73		**1lmi**	131		**1gbs**	185		**1yrw**	291
**1uj8**	73		**1qgv**	131		**2p65**	185		**1U2K**	292
**2CGQ**	74		**1t3y**	131		**1P5F**	186		**1gcu**	292
**2EVB**	74		**2lis**	131		**3FRR**	186		**1lkf**	292
**3ZZP**	74		**1WVH**	132		**3ETP**	187		**1SUU**	293
**1hoe**	74		**3CO1**	132		**2D7J**	188		**3G9T**	294
**3NX6**	75		**3S60**	132		**3ERB**	188		**3SV0**	294
**1hyp**	75		**3TIP**	132		**1jhs**	188		**4D8L**	294
**3Q9P**	76		**1kt9**	132		**1ukf**	188		**4EVF**	294
**1h75**	76		**1nfn**	132		**1wj9**	188		**3c5v**	294
**1VCC**	77		**2EIF**	133		**2rci**	188		**1LCY**	296
**2b8i**	77		**2XGV**	133		**1rgp**	189		**3K8W**	296
**2e3i**	78		**4KEF**	133		**1tua**	189		**2ptd**	296
**2j8b**	78		**1klx**	133		**2R77**	190		**3JSN**	297
**3K3V**	79		**1ZHV**	134		**3A2Z**	190		**1rwr**	297
**3dkm**	79		**2EJX**	134		**3OSX**	190		**2X4L**	298
**1X3O**	80		**2FZP**	134		**1iap**	190		**2Z1E**	298
**2ZQE**	80		**1lu4**	134		**3tss**	190		**1fhu**	298
**3RFI**	80		**2yvq**	134		**3LTJ**	191		**2IM9**	299
**1nh9**	80		**2W0I**	135		**2AP3**	192		**4ETX**	300
**1zzk**	80		**3FLG**	135		**3HZ8**	192		**1a3h**	300
**3LLB**	81		**4LJ1**	135		**4JMI**	192		**2f68**	300
**4F25**	81		**1R62**	136		**2PTH**	193		**2ixm**	300
**1tsf**	81		**3VBC**	136		**3KB5**	193		**2qy9**	300
**2e3h**	81		**1ey4**	136		**1u53**	193		**4G3N**	302
**2o37**	81		**1xgw**	136		**2jay**	193		**1bqc**	302
**2pne**	81		**2f1y**	136		**1MF7**	194		**1k6a**	302
**1ZPW**	82		**2vo8**	136		**2VGA**	194		**2h14**	303
**1uln**	82		**1IFG**	137		**3BOR**	194		**1NTY**	305
**2ckx**	83		**3K8U**	137		**3O0P**	194		**2h2z**	306
**1zeq**	84		**3RDJ**	137		**3VZH**	194		**1arl**	307
**3KZD**	85		**1gak**	137		**4ME2**	194		**2X0C**	308
**4IL7**	85		**2end**	137		**1YZF**	195		**2XBG**	308
**2fq3**	85		**2CXC**	138		**3SH4**	195		**1ak1**	308
**2QVO**	87		**2JHY**	138		**4DDP**	195		**2iy9**	309
**1ptf**	87		**3ZSL**	138		**1nkr**	195		**2p4h**	310

**1ulr**	87		**4EVM**	138		**2VY6**	196		**2YLH**	311
**2i6v**	87		**4NI6**	138		**2osa**	196		**1pgs**	311
**1VJK**	88		**1i2h**	138		**2p52**	196		**3K6U**	312
**4HTI**	88		**1r9w**	138		**4DB6**	197		**3UAH**	312
**1dsl**	88		**2A4D**	139		**4IGI**	197		**1qwk**	312
**1tig**	88		**3O7K**	139		**1cex**	197		**2cyg**	312
**1x6j**	88		**3RZY**	139		**2pb7**	197		**4IQM**	313
**3FDR**	89		**1j74**	139		**1CHD**	198		**1TM2**	314
**2f15**	89		**2in0**	139		**3KT2**	198		**3BB7**	314
**2Q9V**	90		**2nrr**	139		**3P4L**	198		**3LPZ**	314
**2V75**	90		**2FK9**	140		**3W90**	198		**3QH4**	314
**3MX7**	90		**3OBS**	140		**1kzf**	198		**4E9L**	314
**4GS3**	90		**4E9E**	140		**3L9U**	199		**1fcq**	314
**4OIX**	90		**4G08**	140		**4M9K**	199		**1y9u**	314
**2cg7**	90		**1p4p**	140		**2FM9**	201		**3ETV**	315
**3ce7**	90		**1q2y**	140		**1ijb**	202		**2oy7**	315
**1R5Q**	91		**1rss**	140		**1v77**	202		**3EVN**	316
**2GZV**	91		**1OZ9**	141		**4INK**	203		**4J87**	316
**4LTT**	91		**1TZV**	141		**2vfy**	203		**3civ**	316
**1b9w**	91		**2D59**	141		**4B2F**	204		**1NIJ**	317
**2o71**	91		**4H9J**	141		**1dix**	204		**3CML**	317
**4GMQ**	92		**3DFG**	142		**2HLY**	205		**3PST**	317
**2ygs**	92		**1k6k**	142		**1xkr**	205		**4AFV**	317
**1MZL**	93		**1wka**	143		**1gsm**	206		**4K5Q**	317
**3ID4**	93		**1BZ4**	144		**1mix**	206		**1lzl**	317
**2rb8**	93		**1EYH**	144		**1uch**	206		**4LSW**	318
**2VH7**	94		**1KNG**	144		**2eng**	206		**1w7b**	319
**3ID1**	94		**3ILC**	144		**1otm**	207		**3PT5**	320
**3KP8**	94		**4GA2**	144		**3cjw**	207		**2rjd**	321
**3LMO**	94		**4GEI**	144		**3NE0**	208		**3psg**	321
**4O7Q**	94		**1gs9**	144		**3dlm**	208		**1WLY**	322
**1qzm**	94		**1l2h**	144		**3T0H**	209		**3R2G**	323
**3JU0**	95		**1o8x**	144		**2erf**	209		**4FH3**	323
**3JVE**	95		**2QEV**	145		**2QHT**	210		**1gxn**	323
**1lpl**	95		**3MM4**	145		**3TUA**	210		**3app**	323
**2qt4**	95		**1q5z**	145		**2z84**	210		**1wer**	324
**2WJ5**	96		**1srv**	145		**1DZF**	211		**1xfk**	324
**3RJP**	96		**2p5d**	145		**4IBN**	211		**3IM1**	325
**1mwp**	96		**1NIG**	146		**2ogq**	211		**3N11**	325
**1t2i**	96		**3FH2**	146		**1ojq**	212		**3N2T**	327
**1u9p**	96		**4J5Q**	146		**1zd8**	212		**4J0W**	327

**1z21**	96		**1jmw**	146		**1IO2**	213		**3GMS**	331
**2ptv**	96		**1O6D**	147		**4JNF**	213		**1fo9**	331
**2BK8**	97		**2QPW**	147		**1RZ2**	214		**2z0m**	331
**2CWR**	97		**4G78**	147		**3FTJ**	214		**1ceo**	332
**3ONJ**	97		**1khi**	147		**2d4x**	214		**1ri6**	333
**4EL6**	97		**3G9B**	148		**1P3C**	215		**2cy7**	333
**1lou**	97		**3JZZ**	148		**1V8E**	217		**3VJ8**	335
**1J27**	98		**1ng6**	148		**1gpp**	217		**1xix**	335
**1ln4**	98		**1rj1**	148		**3ESU**	218		**1i9y**	336
**1bm8**	99		**2ESK**	149		**3VNE**	218		**2bjq**	340
**1cqy**	99		**2Y9F**	149		**4FD6**	218		**2OKT**	342
**1opc**	99		**3A0X**	149		**4JZC**	218		**3HR8**	342
**2j9v**	99		**1x91**	149		**1tk1**	219		**1nj4**	343
**2pcy**	99		**2b1k**	149		**1mw7**	220		**1Z15**	344
**2pko**	99		**2OP6**	150		**1wnh**	220		**3I2N**	345
**2yxf**	99		**3N0K**	150		**1RC9**	221		**3UGU**	345
**2JLI**	100		**3PR9**	150		**2YMO**	221		**4AM1**	345
**1eoe**	100		**1amx**	150		**3PKV**	221		**3PTE**	347
**1jos**	100		**1bj7**	150		**1ois**	221		**4AD1**	349
**1w41**	100		**2jcp**	150		**2RFA**	222		**3Q1C**	351
**2PE8**	101		**2ywn**	150		**3KR9**	222		**1SNT**	352
**3BZT**	101		**1ZUH**	151		**3U0V**	222		**3AAP**	353
**1P1L**	102		**3SZ7**	151		**4G54**	222		**3IU0**	354
**3ADY**	102		**2q5x**	151		**1bol**	222		**4HDJ**	355
**3EAZ**	102		**2OEB**	152		**1oa4**	222		**2pge**	356
**3ISU**	102		**2VY8**	152		**2a6z**	222		**3PZ9**	357
**3KT9**	102		**3TOW**	152		**2ahn**	222		**3IVF**	358
**4DOT**	102		**4AGK**	152		**1UOH**	223		**1qcx**	359
**1fhg**	102		**1dvo**	152		**2WNK**	223		**1eur**	361
**3HAK**	103		**1jl1**	152		**1uai**	223		**1wos**	361
**2FD4**	104		**1hzt**	153		**3ckf**	223		**1GCE**	362
**2X5P**	104		**3eye**	153		**1tje**	224		**3GD0**	362
**3VFI**	104		**3PIW**	154		**2e3s**	224		**1cem**	363
**1WWC**	105		**2pwq**	154		**4EUG**	225		**1k30**	363
**3AG7**	105		**2CZT**	155		**2ZFY**	226		**1nc5**	363
**1aaj**	105		**3Q6B**	155		**4JMP**	226		**2ZQ5**	365
**1m5i**	105		**1icx**	155		**1g8a**	227		**3cbh**	365
**1tqg**	105		**1jyh**	155		**1k1b**	228		**2PEF**	366
**2VQ4**	106		**2rer**	155		**2ahe**	228		**3KCI**	366
**3A4C**	106		**3HNY**	156		**4ERN**	229		**3V55**	368
**4EO0**	106		**3ML3**	156		**1yhh**	229		**1fnf**	368

**4GGR**	106		**3KH7**	157		**3PG4**	230		**3PZF**	369
**1ew4**	106		**3MEW**	157		**3SK9**	230		**3KJT**	370
**2frg**	106		**3NR5**	157		**4B89**	230		**3NE4**	370
**2PPN**	107		**3V1Q**	157		**1p9q**	230		**2zhv**	370
**3DJ9**	107		**4J8Y**	157		**2PET**	231		**4IC4**	372
**1wpa**	107		**1tol**	157		**2eyi**	234		**1c3p**	372
**1xaw**	107		**1txj**	157		**2g5x**	234		**2FEZ**	373
**3cx2**	107		**4ESS**	158		**1WR2**	235		**2HY7**	373
**3HNX**	108		**1bgc**	158		**2X8X**	235		**1vf8**	373
**3VVV**	108		**1hka**	158		**1z3y**	236		**2Q43**	375
**3W56**	108		**1Q42**	159		**3crm**	236		**3ANJ**	376
**4I2T**	108		**3CSR**	159		**3DU1**	237		**3M7D**	376
**1bkr**	108		**3IXR**	159		**2bjv**	237		**4IZO**	376
**2i1u**	108		**1t5i**	159		**3PHS**	238		**1bhe**	376
**2FO3**	109		**3V46**	160		**1xdz**	238		**2b78**	376
**2R2Y**	109		**4DT4**	160		**2lao**	238		**1e4f**	378
**1TQ3**	110		**1jvw**	160		**3seb**	238		**1EDG**	380
**4HQA**	110		**1K95**	161		**3TCQ**	239		**2sil**	381
**2WWE**	111		**1Z7C**	161		**3FTD**	240		**2G5D**	382
**4MZ2**	111		**2FR2**	161		**1iz4**	241		**1wyc**	384
**1roa**	111		**3HA9**	161		**3KVD**	242		**1fc9**	386
**1qau**	112		**4CHE**	161		**1dua**	242		**3H2G**	387
**2og3**	112		**2DYI**	162		**2baa**	243		**3G6L**	388
**3FKC**	113		**1l3k**	163		**2ad1**	244		**2ie8**	390
**3ONH**	113		**1fl0**	164		**4DIU**	245		**2WN4**	391
**4B50**	113		**1p7s**	164		**4E40**	245		**2pbo**	394
**1noa**	113		**1rl6**	164		**1qts**	247		**1io1**	395
**2hc8**	113		**2ova**	164		**2NXC**	249		**3GRH**	397
**1SAU**	114		**2FPH**	165		**2OG4**	249		**2I49**	398
**3IU5**	114		**3EJG**	165		**1o0x**	249		**2GGO**	401
**2o0q**	114		**3G39**	165		**2ilr**	249		**3OGG**	404
**3EOD**	115		**1kxo**	165		**2fbo**	250		**1h13**	404
**3K1H**	115		**1s21**	165		**3HC7**	252		**1DFA**	405
**3LS0**	115		**2hwx**	165		**1vin**	252		**3GRE**	408
**2e7v**	115		**2obi**	165		**1xqo**	253		**3DMS**	413
**1NPU**	116		**3bci**	165		**2f1n**	253		**3bok**	416
**1bea**	116		**2X3M**	166		**3VN5**	255		**3ACP**	417
**1jpe**	116		**3EJF**	166		**1rl0**	255		**1gso**	419
**1xte**	116		**4A02**	166		**1fqn**	257		**3cj1**	419
**2z14**	116		**1qnt**	166		**3G40**	258		**1sqg**	424
**3L78**	117		**4ACJ**	167		**3I47**	259		**1yks**	431

**3ZK0**	117		**1ow1**	167		**1ES5**	260		**1l2l**	432
**4I6X**	117		**1pgv**	167		**3ZNY**	260		**1tuo**	437
**2FI9**	118		**3d79**	167		**1eg3**	260		**3OF7**	438
**3RVC**	118		**3H6Q**	168		**1es6**	260		**3CB6**	441
**1r9h**	118		**4E2U**	168		**1arb**	263		**3P1W**	442
**1UNP**	119		**4JQF**	168		**4M67**	264		**4IDH**	451
**3S0A**	119		**1nwa**	168		**1OUV**	265		**2ece**	455
**1wou**	119		**2rkq**	168		**3ILS**	265		**2r60**	456
**2pnd**	119		**2sga**	168		**3tgl**	265		**3csg**	458
**2WZ9**	120		**3BQE**	169		**2XMZ**	266		**1ZCJ**	459
**3LF9**	120		**3WI0**	169		**4FBR**	266		**1vjs**	469
**4GCO**	120		**1v7q**	169		**2of3**	266		**4J0U**	471
**1gyu**	120		**3eo5**	169		**1AKO**	268		**1U09**	476
**1gyv**	120		**4BXP**	170		**4IH1**	268		**3VSR**	493
**1x6z**	120		**1o9z**	170		**1uek**	268		**1cwy**	500
**2fj8**	120		**1oxj**	170						

Table SM-13. Explained variance results for the first 159 components of the PCA carried out with 3D and sequence-based protein descriptors.

Component	Initial Eigenvalues			Rotation Sums of Squared Loadings		
	Total	% of Variance	Cumulative %	Total	% of Variance	Cumulative %
1	687.632	34.416	34.416	682.004	34.134	34.134
2	138.883	6.951	41.367	102.544	5.132	39.267
3	103.920	5.201	46.568	63.246	3.165	42.432
4	97.241	4.867	51.435	62.705	3.138	45.571
5	71.954	3.601	55.037	62.338	3.120	48.691
6	65.273	3.267	58.303	60.386	3.022	51.713
7	54.942	2.750	61.053	57.847	2.895	54.608
8	41.458	2.075	63.128	53.682	2.687	57.295
9	35.962	1.800	64.928	42.571	2.131	59.426
10	33.363	1.670	66.598	40.161	2.010	61.436
11	32.463	1.625	68.223	37.083	1.856	63.292
12	27.282	1.365	69.588	37.012	1.852	65.144
13	25.803	1.291	70.880	35.393	1.771	66.916
14	23.303	1.166	72.046	33.545	1.679	68.594
15	21.838	1.093	73.139	31.673	1.585	70.180
16	20.106	1.006	74.145	22.307	1.116	71.296
17	17.851	.893	75.039	17.602	.881	72.177
18	15.186	.760	75.799	17.168	.859	73.036
19	14.615	.731	76.530	15.851	.793	73.830
20	13.256	.663	77.194	14.847	.743	74.573
21	12.368	.619	77.813	14.513	.726	75.299
22	11.264	.564	78.376	9.443	.473	75.772
23	9.901	.496	78.872	9.097	.455	76.227
24	9.177	.459	79.331	8.288	.415	76.642
25	8.439	.422	79.754	7.956	.398	77.040
26	8.145	.408	80.161	7.660	.383	77.424
27	7.880	.394	80.556	7.499	.375	77.799
28	7.445	.373	80.928	7.241	.362	78.161
29	6.996	.350	81.278	7.114	.356	78.517
30	6.737	.337	81.616	7.097	.355	78.873
31	6.197	.310	81.926	6.897	.345	79.218
32	5.592	.280	82.206	5.930	.297	79.515
33	5.156	.258	82.464	5.429	.272	79.786
34	5.020	.251	82.715	5.188	.260	80.046
35	4.903	.245	82.960	5.180	.259	80.305
36	4.881	.244	83.205	4.812	.241	80.546

37	4.748	.238	83.442	4.473	.224	80.770
38	4.509	.226	83.668	4.473	.224	80.994
39	4.443	.222	83.890	4.402	.220	81.214
40	4.322	.216	84.107	4.220	.211	81.425
41	4.140	.207	84.314	4.161	.208	81.634
42	4.077	.204	84.518	4.150	.208	81.841
43	3.839	.192	84.710	4.039	.202	82.043
44	3.825	.191	84.902	3.933	.197	82.240
45	3.694	.185	85.086	3.817	.191	82.431
46	3.653	.183	85.269	3.788	.190	82.621
47	3.584	.179	85.449	3.735	.187	82.808
48	3.510	.176	85.624	3.713	.186	82.994
49	3.391	.170	85.794	3.660	.183	83.177
50	3.346	.167	85.961	3.636	.182	83.359
51	3.278	.164	86.126	3.611	.181	83.540
52	3.230	.162	86.287	3.542	.177	83.717
53	3.202	.160	86.448	3.425	.171	83.888
54	3.094	.155	86.602	3.401	.170	84.058
55	3.062	.153	86.756	3.386	.169	84.228
56	3.027	.151	86.907	3.381	.169	84.397
57	3.013	.151	87.058	3.269	.164	84.561
58	2.950	.148	87.206	3.265	.163	84.724
59	2.844	.142	87.348	3.260	.163	84.887
60	2.829	.142	87.490	3.234	.162	85.049
61	2.721	.136	87.626	3.218	.161	85.210
62	2.700	.135	87.761	3.110	.156	85.366
63	2.620	.131	87.892	3.091	.155	85.521
64	2.573	.129	88.021	3.087	.155	85.675
65	2.510	.126	88.146	3.086	.154	85.830
66	2.466	.123	88.270	3.056	.153	85.983
67	2.416	.121	88.391	2.893	.145	86.127
68	2.402	.120	88.511	2.800	.140	86.268
69	2.316	.116	88.627	2.799	.140	86.408
70	2.304	.115	88.742	2.794	.140	86.547
71	2.295	.115	88.857	2.772	.139	86.686
72	2.273	.114	88.971	2.760	.138	86.824
73	2.209	.111	89.081	2.735	.137	86.961
74	2.189	.110	89.191	2.650	.133	87.094
75	2.142	.107	89.298	2.645	.132	87.226
76	2.130	.107	89.405	2.622	.131	87.357
77	2.094	.105	89.509	2.584	.129	87.487

78	2.043	.102	89.612	2.521	.126	87.613
79	2.025	.101	89.713	2.459	.123	87.736
80	2.014	.101	89.814	2.457	.123	87.859
81	1.961	.098	89.912	2.449	.123	87.982
82	1.933	.097	90.009	2.445	.122	88.104
83	1.910	.096	90.104	2.390	.120	88.224
84	1.871	.094	90.198	2.351	.118	88.341
85	1.865	.093	90.291	2.350	.118	88.459
86	1.847	.092	90.384	2.323	.116	88.575
87	1.840	.092	90.476	2.245	.112	88.687
88	1.808	.091	90.566	2.244	.112	88.800
89	1.797	.090	90.656	2.213	.111	88.911
90	1.758	.088	90.744	2.195	.110	89.020
91	1.744	.087	90.832	2.194	.110	89.130
92	1.724	.086	90.918	2.187	.109	89.240
93	1.690	.085	91.002	2.172	.109	89.348
94	1.668	.084	91.086	2.171	.109	89.457
95	1.664	.083	91.169	2.125	.106	89.563
96	1.651	.083	91.252	2.111	.106	89.669
97	1.621	.081	91.333	2.103	.105	89.774
98	1.612	.081	91.414	2.088	.105	89.879
99	1.579	.079	91.493	2.075	.104	89.983
100	1.573	.079	91.571	2.040	.102	90.085
101	1.544	.077	91.649	2.040	.102	90.187
102	1.531	.077	91.725	2.028	.102	90.288
103	1.508	.075	91.801	2.016	.101	90.389
104	1.486	.074	91.875	1.987	.099	90.489
105	1.464	.073	91.949	1.970	.099	90.587
106	1.458	.073	92.021	1.965	.098	90.686
107	1.452	.073	92.094	1.962	.098	90.784
108	1.436	.072	92.166	1.952	.098	90.882
109	1.421	.071	92.237	1.921	.096	90.978
110	1.402	.070	92.307	1.905	.095	91.073
111	1.391	.070	92.377	1.897	.095	91.168
112	1.369	.069	92.445	1.894	.095	91.263
113	1.341	.067	92.513	1.881	.094	91.357
114	1.338	.067	92.580	1.876	.094	91.451
115	1.319	.066	92.646	1.874	.094	91.545
116	1.309	.066	92.711	1.867	.093	91.638
117	1.295	.065	92.776	1.852	.093	91.731
118	1.276	.064	92.840	1.830	.092	91.822

119	1.267	.063	92.903	1.759	.088	91.910
120	1.263	.063	92.966	1.759	.088	91.998
121	1.247	.062	93.029	1.752	.088	92.086
122	1.242	.062	93.091	1.691	.085	92.171
123	1.233	.062	93.153	1.674	.084	92.255
124	1.205	.060	93.213	1.672	.084	92.338
125	1.202	.060	93.273	1.665	.083	92.422
126	1.194	.060	93.333	1.647	.082	92.504
127	1.183	.059	93.392	1.632	.082	92.586
128	1.158	.058	93.450	1.631	.082	92.667
129	1.152	.058	93.508	1.617	.081	92.748
130	1.138	.057	93.565	1.612	.081	92.829
131	1.132	.057	93.621	1.606	.080	92.909
132	1.119	.056	93.677	1.596	.080	92.989
133	1.116	.056	93.733	1.583	.079	93.068
134	1.108	.055	93.789	1.571	.079	93.147
135	1.105	.055	93.844	1.555	.078	93.225
136	1.103	.055	93.899	1.551	.078	93.303
137	1.093	.055	93.954	1.547	.077	93.380
138	1.080	.054	94.008	1.545	.077	93.457
139	1.063	.053	94.061	1.543	.077	93.535
140	1.061	.053	94.114	1.535	.077	93.611
141	1.054	.053	94.167	1.533	.077	93.688
142	1.041	.052	94.219	1.530	.077	93.765
143	1.018	.051	94.270	1.526	.076	93.841
144	1.008	.050	94.320	1.522	.076	93.917
145	1.002	.050	94.371	1.511	.076	93.993
146	.986	.049	94.420	1.510	.076	94.068
147	.978	.049	94.469	1.503	.075	94.144
148	.970	.049	94.517	1.496	.075	94.219
149	.962	.048	94.566	1.495	.075	94.293
150	.954	.048	94.613	1.491	.075	94.368
151	.948	.047	94.661	1.484	.074	94.442
152	.942	.047	94.708	1.481	.074	94.516
153	.932	.047	94.755	1.475	.074	94.590
154	.930	.047	94.801	1.473	.074	94.664
155	.916	.046	94.847	1.463	.073	94.737
156	.907	.045	94.892	1.451	.073	94.810
157	.900	.045	94.938	1.447	.072	94.882
158	.885	.044	94.982	1.442	.072	94.954
159	.880	.044	95.026	1.427	.071	95.026

Table SM-14. Explained variance of the PCA carried out with extracted components of PROFEAT, PROTEIN RECON and PROTDCAL.

Component	Initial Eigenvalues			Rotation Sums of Squared Loadings				
	Total	% of Variance	Cumulative %	Total	% of Variance	Cumulative %	PROTDCAL	PROFEAT
1	2.914	1.230	1.230	2.895	1.222	1.222	1.222	1.222
2	1.987	.838	2.068	1.643	.693	1.915	.693	.693
3	1.983	.837	2.905	1.639	.692	2.607	.692	
4	1.979	.835	3.740	1.613	.681	3.287	.681	
5	1.968	.830	4.570	1.610	.679	3.967	.679	
6	1.962	.828	5.398	1.572	.663	4.630	.663	
7	1.959	.827	6.225	1.530	.646	5.276	.646	
8	1.949	.823	7.047	1.440	.608	5.883	.608	
9	1.948	.822	7.869	1.427	.602	6.485	.602	
10	1.941	.819	8.688	1.403	.592	7.077	.592	
11	1.938	.818	9.505	1.352	.570	7.648	.570	
12	1.921	.811	10.316	1.336	.564	8.211	.564	
13	1.905	.804	11.120	1.323	.558	8.770	.558	
14	1.898	.801	11.921	1.319	.557	9.326		.557
15	1.888	.796	12.717	1.309	.552	9.879	.552	
16	1.875	.791	13.508	1.294	.546	10.424		.546
17	1.850	.780	14.289	1.291	.545	10.969	.545	
18	1.834	.774	15.063	1.288	.543	11.512	.543	
19	1.809	.763	15.826	1.284	.542	12.054	.542	
20	1.796	.758	16.584	1.284	.542	12.596	.542	
21	1.756	.741	17.325	1.281	.541	13.137		.541
22	1.735	.732	18.057	1.272	.537	13.673		.537
23	1.718	.725	18.782	1.269	.536	14.209	.536	
24	1.705	.719	19.501	1.267	.534	14.743		.534
25	1.696	.716	20.217	1.264	.533	15.276		.533
26	1.683	.710	20.927	1.262	.533	15.809		.533
27	1.658	.700	21.627	1.261	.532	16.341		.532
28	1.654	.698	22.325	1.260	.532	16.873	.532	
29	1.648	.695	23.020	1.249	.527	17.400		.527
30	1.640	.692	23.712	1.234	.521	17.920		.521
31	1.628	.687	24.399	1.227	.518	18.438		.518
32	1.616	.682	25.080	1.222	.516	18.954	.516	
33	1.606	.677	25.758	1.220	.515	19.469	.515	
34	1.601	.675	26.433	1.219	.514	19.983		.514
35	1.580	.667	27.100	1.219	.514	20.497		.514
36	1.578	.666	27.766	1.212	.511	21.008		.511

37	1.563	.659	28.425	1.211	.511	21.519		.511
38	1.561	.659	29.084	1.211	.511	22.030		.511
39	1.547	.653	29.737	1.208	.510	22.540		.510
40	1.541	.650	30.387	1.206	.509	23.049	.509	
41	1.531	.646	31.033	1.204	.508	23.557		.508
42	1.527	.644	31.677	1.203	.507	24.064		.507
43	1.520	.641	32.318	1.198	.505	24.570		.505
44	1.516	.639	32.958	1.198	.505	25.075	.505	
45	1.507	.636	33.593	1.197	.505	25.580		.505
46	1.494	.630	34.224	1.196	.505	26.085	.505	
47	1.492	.630	34.853	1.196	.505	26.589	.505	
48	1.481	.625	35.478	1.195	.504	27.093		.504
49	1.476	.623	36.101	1.194	.504	27.597	.504	
50	1.469	.620	36.721	1.194	.504	28.101		.504
51	1.457	.615	37.336	1.193	.504	28.605		.504
52	1.453	.613	37.949	1.190	.502	29.107	.502	
53	1.444	.609	38.558	1.190	.502	29.609		.502
54	1.430	.603	39.161	1.190	.502	30.111		.502
55	1.419	.599	39.760	1.189	.502	30.612		.502
56	1.415	.597	40.357	1.188	.501	31.114		.501
57	1.412	.596	40.953	1.185	.500	31.614		.500
58	1.408	.594	41.547	1.185	.500	32.114		.500
59	1.398	.590	42.137	1.184	.500	32.614	.500	
60	1.394	.588	42.726	1.184	.500	33.113	.500	
61	1.386	.585	43.310	1.182	.499	33.612		.499
62	1.378	.581	43.892	1.180	.498	34.110		.498
63	1.364	.575	44.467	1.179	.498	34.608		.498
64	1.359	.574	45.041	1.177	.497	35.104	.497	
65	1.356	.572	45.613	1.177	.497	35.601	.497	
66	1.348	.569	46.182	1.176	.496	36.098		.496
67	1.344	.567	46.749	1.172	.495	36.592		.495
68	1.338	.564	47.313	1.171	.494	37.086		.494
69	1.335	.563	47.876	1.171	.494	37.581		.494
70	1.324	.559	48.435	1.171	.494	38.074		.494
71	1.312	.553	48.988	1.169	.493	38.568		.493
72	1.307	.551	49.540	1.168	.493	39.061		.493
73	1.296	.547	50.086	1.166	.492	39.553		.492
74	1.293	.546	50.632	1.166	.492	40.045		.492
75	1.280	.540	51.172	1.166	.492	40.537		.492
76	1.276	.538	51.711	1.166	.492	41.028		.492
77	1.275	.538	52.249	1.165	.492	41.520		.492

78	1.267	.535	52.783	1.163	.491	42.011		.491
79	1.263	.533	53.316	1.162	.490	42.501		.490
80	1.257	.530	53.846	1.162	.490	42.992		.490
81	1.244	.525	54.371	1.161	.490	43.481		.490
82	1.238	.522	54.893	1.159	.489	43.970		.489
83	1.231	.519	55.413	1.158	.489	44.459	.489	
84	1.221	.515	55.928	1.157	.488	44.947		.488
85	1.220	.515	56.443	1.156	.488	45.435		.488
86	1.215	.513	56.955	1.155	.487	45.922	.487	
87	1.210	.510	57.466	1.153	.487	46.409	.487	
88	1.203	.508	57.974	1.153	.486	46.895		.486
89	1.199	.506	58.479	1.152	.486	47.381		.486
90	1.190	.502	58.981	1.152	.486	47.868		.486
91	1.186	.500	59.482	1.152	.486	48.353	.486	
92	1.178	.497	59.979	1.150	.485	48.839	.485	
93	1.166	.492	60.471	1.149	.485	49.324	.485	
94	1.162	.490	60.961	1.149	.485	49.809	.485	
95	1.157	.488	61.450	1.149	.485	50.293		.485
96	1.146	.483	61.933	1.149	.485	50.778		.485
97	1.141	.481	62.414	1.148	.484	51.262		.484
98	1.134	.478	62.893	1.148	.484	51.747		.484
99	1.124	.474	63.367	1.146	.484	52.230	.484	
100	1.117	.471	63.838	1.145	.483	52.714		.483
101	1.102	.465	64.303	1.144	.483	53.196		.483
102	1.100	.464	64.767	1.143	.482	53.679		.482
103	1.092	.461	65.228	1.143	.482	54.161	.482	
104	1.088	.459	65.687	1.142	.482	54.642	.482	
105	1.084	.457	66.145	1.142	.482	55.124		.482
106	1.080	.456	66.601	1.141	.482	55.606		.482
107	1.066	.450	67.051	1.141	.481	56.087	.481	
108	1.064	.449	67.499	1.139	.481	56.568		.481
109	1.054	.445	67.944	1.137	.480	57.048		.480
110	1.042	.440	68.384	1.137	.480	57.527	.480	
111	1.029	.434	68.818	1.137	.480	58.007	.480	
112	1.002	.423	69.241	1.137	.480	58.487		.480
113	1.002	.423	69.664	1.137	.480	58.966	.480	
114	1.002	.423	70.086	1.137	.480	59.446	.480	
115	1.001	.423	70.509	1.133	.478	59.924	.478	
116	1.001	.422	70.931	1.133	.478	60.402	.478	
117	1.001	.422	71.354	1.131	.477	60.879		.477
118	1.001	.422	71.776	1.131	.477	61.356	.477	

119	1.001	.422	72.199	1.129	.476	61.833	.476	
120	1.001	.422	72.621	1.128	.476	62.308		.476
121	1.001	.422	73.043	1.127	.476	62.784	.476	
122	1.001	.422	73.465	1.126	.475	63.259		.475
123	1.001	.422	73.888	1.125	.475	63.734	.475	
124	1.000	.422	74.310	1.125	.475	64.208	.475	
125	.974	.411	74.720	1.124	.474	64.683	.474	
126	.971	.410	75.130	1.123	.474	65.157	.474	
127	.958	.404	75.535	1.123	.474	65.631	.474	
128	.943	.398	75.933	1.122	.473	66.104	.473	
129	.935	.395	76.327	1.120	.473	66.577		.473
130	.933	.394	76.721	1.117	.471	67.048		.471
131	.921	.389	77.110	1.116	.471	67.519	.471	
132	.916	.387	77.496	1.116	.471	67.990		.471
133	.911	.385	77.881	1.116	.471	68.461		.471
134	.910	.384	78.265	1.115	.470	68.931	.470	
135	.902	.381	78.646	1.115	.470	69.401		.470
136	.899	.379	79.025	1.113	.469	69.871	.469	
137	.883	.372	79.397	1.112	.469	70.340		.469
138	.871	.368	79.765	1.110	.468	70.808		.468
139	.868	.366	80.131	1.110	.468	71.277	.468	
140	.860	.363	80.494	1.110	.468	71.745		.468
141	.855	.361	80.855	1.109	.468	72.213	.468	
142	.844	.356	81.211	1.109	.468	72.681	.468	
143	.837	.353	81.564	1.108	.468	73.148	.468	
144	.833	.351	81.915	1.107	.467	73.616	.467	
145	.822	.347	82.262	1.107	.467	74.083	.467	
146	.814	.343	82.606	1.106	.467	74.549		.467
147	.810	.342	82.948	1.104	.466	75.015	.466	
148	.804	.339	83.287	1.104	.466	75.481	.466	
149	.798	.337	83.623	1.104	.466	75.947	.466	
150	.788	.333	83.956	1.104	.466	76.413		.466
151	.786	.332	84.288	1.103	.466	76.878	.466	
152	.782	.330	84.618	1.102	.465	77.343	.465	
153	.780	.329	84.947	1.102	.465	77.808		.465
154	.769	.324	85.271	1.102	.465	78.273	.465	
155	.760	.321	85.592	1.102	.465	78.738		.465
156	.758	.320	85.912	1.101	.465	79.203	.465	
157	.744	.314	86.226	1.099	.464	79.666	.464	
158	.738	.311	86.537	1.097	.463	80.129	.463	
159	.735	.310	86.847	1.097	.463	80.592	.463	

160	.727	.307	87.154	1.097	.463	81.055	.463	
161	.726	.306	87.460	1.094	.462	81.516	.462	
162	.722	.305	87.765	1.094	.462	81.978	.462	
163	.708	.299	88.063	1.094	.461	82.439	.461	
164	.701	.296	88.359	1.092	.461	82.900	.461	
165	.693	.293	88.652	1.092	.461	83.361		.461
166	.688	.290	88.942	1.092	.461	83.822	.461	
167	.677	.286	89.228	1.091	.460	84.282	.460	
168	.667	.281	89.509	1.091	.460	84.742	.460	
169	.663	.280	89.789	1.090	.460	85.202		.460
170	.656	.277	90.065	1.088	.459	85.661	.459	
171	.653	.276	90.341	1.088	.459	86.121	.459	
172	.645	.272	90.613	1.088	.459	86.580		.459
173	.636	.268	90.882	1.087	.459	87.038	.459	
174	.632	.267	91.148	1.086	.458	87.497		.458
175	.619	.261	91.410	1.086	.458	87.955	.458	
176	.610	.257	91.667	1.086	.458	88.413	.458	
177	.608	.257	91.924	1.085	.458	88.871	.458	
178	.604	.255	92.178	1.084	.457	89.328		.457
179	.594	.251	92.429	1.078	.455	89.783		.455
180	.591	.249	92.678	1.078	.455	90.238	.455	
181	.583	.246	92.924	1.077	.455	90.692	.455	
182	.581	.245	93.169	1.077	.455	91.147	.455	
183	.567	.239	93.409	1.075	.453	91.600	.453	
184	.555	.234	93.643	1.073	.453	92.053	.453	
185	.548	.231	93.874	1.072	.452	92.505	.452	
186	.545	.230	94.104	1.072	.452	92.958	.452	
187	.530	.224	94.328	1.068	.451	93.408	.451	
188	.526	.222	94.550	1.064	.449	93.858	.449	
189	.520	.219	94.769	1.063	.449	94.306	.449	
190	.508	.214	94.983	1.061	.448	94.754	.448	
191	.505	.213	95.196	1.049	.442	95.196	.442	
192	.494	.209	95.405				**(Sum) 51.813**	**(Sum) 45.298**
193	.486	.205	95.610					
194	.482	.203	95.813					
195	.475	.200	96.014					
196	.469	.198	96.211					
197	.461	.194	96.406					
198	.454	.192	96.597					
199	.438	.185	96.782					
200	.433	.183	96.965					

201	.422	.178	97.143					
202	.417	.176	97.319					
203	.398	.168	97.487					
204	.395	.167	97.654					
205	.387	.163	97.817					
206	.371	.157	97.974					
207	.363	.153	98.127					
208	.351	.148	98.275					
209	.346	.146	98.421					
210	.336	.142	98.563					
211	.319	.135	98.698					
212	.308	.130	98.827					
213	.296	.125	98.952					
214	.282	.119	99.072					
215	.267	.112	99.184					
216	.242	.102	99.286					
217	.207	.087	99.373					
218	.193	.081	99.454					
219	.172	.072	99.527					
220	.152	.064	99.591					
221	.127	.054	99.645					
222	.110	.046	99.691					
223	.104	.044	99.735					
224	.093	.039	99.774					
225	.078	.033	99.807					
226	.070	.030	99.837					
227	.065	.027	99.864					
228	.056	.024	99.888					
229	.052	.022	99.910					
230	.045	.019	99.929					
231	.041	.017	99.946					
232	.036	.015	99.961					
233	.035	.015	99.976					
234	.022	.009	99.985					
235	.020	.008	99.993					
236	.015	.006	100.000					
237	.001	.000	100.000					
Extraction Method: Principal Component Analysis.								

Table SM-15. Runtime values per descriptor per protein for different families of features.

		Structure-based (time: sec)			Sequence-based (time: sec)	
PDB ID	Length	Thermodynamic (3D)	Topographic	Topographic (CLQ out)	Thermodynamic (2D)	Property-based
pdb1yzm	46	3.92E-05	2.27E-05	2.00E-05	3.02E-05	1.55E-05
3I8Z	50	3.98E-05	2.63E-05	2.08E-05	3.02E-05	1.55E-05
pdb3ca7	50	3.89E-05	2.55E-05	2.08E-05	3.02E-05	1.55E-05
pdb2gkt	51	3.88E-05	2.36E-05	2.00E-05	1.03E-05	9.33E-06
2ERW	53	3.67E-05	2.72E-05	2.00E-05	3.02E-05	1.57E-05
4G3O	53	3.89E-05	2.45E-05	2.08E-05	9.65E-06	1.55E-05
2CMP	56	4.10E-05	3.00E-05	2.17E-05	3.02E-05	1.55E-05
1YP5	58	4.32E-05	2.63E-05	2.17E-05	2.96E-05	1.87E-05
pdb1zlm	58	4.53E-05	2.91E-05	2.35E-05	3.02E-05	1.55E-05
pdb3cqt	58	4.54E-05	3.00E-05	2.43E-05	3.02E-05	1.55E-05
pdb4pti	58	4.32E-05	2.99E-05	2.26E-05	3.02E-05	1.55E-05
pdb1mhn	59	4.32E-05	2.73E-05	2.26E-05	3.02E-05	1.55E-05
pdb1orc	59	4.96E-05	3.00E-05	2.44E-05	3.02E-05	1.87E-05
pdb2fjz	59	4.32E-05	2.82E-05	2.35E-05	3.02E-05	1.87E-05
pdb1tud	60	4.53E-05	2.82E-05	2.26E-05	3.02E-05	2.18E-05
2IGD	61	4.53E-05	2.63E-05	2.17E-05	3.02E-05	1.55E-05
2PST	61	5.40E-05	3.73E-05	2.69E-05	3.02E-05	1.55E-05
pdb1i2t	61	4.10E-05	2.63E-05	2.26E-05	1.03E-05	1.55E-05
pdb1ku3	61	4.96E-05	2.91E-05	2.69E-05	3.02E-05	1.55E-05
pdb1l2p	61	4.10E-05	4.45E-05	2.08E-05	3.02E-05	1.57E-05
2CJJ	63	5.18E-05	2.91E-05	2.52E-05	1.03E-05	1.87E-05
pdb1r69	63	4.96E-05	2.81E-05	2.44E-05	1.03E-05	1.55E-05
pdb1uoy	64	4.53E-05	2.82E-05	2.26E-05	3.99E-05	1.57E-05
pdb1ypc	64	4.73E-05	3.09E-05	2.35E-05	3.02E-05	1.57E-05
2HDZ	66	5.18E-05	3.36E-05	2.61E-05	3.02E-05	1.87E-05
2OCH	66	4.96E-05	2.91E-05	2.69E-05	3.02E-05	1.55E-05
3IDW	66	5.82E-05	3.54E-05	2.78E-05	3.02E-05	1.87E-05
pdb1tg0	66	4.75E-05	3.00E-05	2.44E-05	9.65E-06	1.23E-05
3RDY	67	4.97E-05	3.36E-05	2.43E-05	3.02E-05	1.87E-05
4HCS	67	5.18E-05	3.27E-05	2.61E-05	2.96E-05	1.55E-05
pdb1yu5	67	4.75E-05	2.82E-05	2.61E-05	3.02E-05	1.87E-05
pdb2hlr	67	5.18E-05	3.09E-05	2.61E-05	9.65E-06	1.55E-05
pdb1xak	68	5.18E-05	3.09E-05	2.61E-05	3.02E-05	1.25E-05
pdb2fht	68	4.96E-05	3.45E-05	2.61E-05	3.02E-05	1.55E-05
pdb1mjc	69	4.54E-05	3.18E-05	2.43E-05	3.02E-05	1.55E-05
pdb1pgx	70	5.38E-05	3.63E-05	2.61E-05	2.06E-05	1.85E-05
3VDJ	71	5.18E-05	3.38E-05	2.60E-05	3.02E-05	1.55E-05

3ADG	72	5.40E-05	4.91E-05	2.61E-05	4.05E-05	1.55E-05
3UCI	72	4.75E-05	3.27E-05	2.43E-05	3.02E-05	1.55E-05
pdb2vc8	72	5.18E-05	3.36E-05	2.70E-05	3.02E-05	1.87E-05
pdb1uj8	73	6.03E-05	4.00E-05	2.95E-05	3.02E-05	1.87E-05
2CGQ	74	5.60E-05	3.45E-05	2.78E-05	3.02E-05	1.87E-05
2EVB	74	4.75E-05	3.09E-05	2.43E-05	3.02E-05	1.57E-05
3ZZP	74	5.40E-05	3.27E-05	2.69E-05	4.05E-05	1.55E-05
pdb1hoe	74	5.40E-05	3.27E-05	2.61E-05	3.02E-05	1.57E-05
3NX6	75	5.40E-05	3.27E-05	2.75E-05	2.96E-05	1.87E-05
pdb1hyp	75	5.38E-05	3.27E-05	2.69E-05	2.06E-05	1.87E-05
3Q9P	76	5.40E-05	3.54E-05	2.69E-05	3.02E-05	1.87E-05
pdb1h75	76	5.60E-05	3.36E-05	2.69E-05	1.03E-05	1.55E-05
1VCC	77	6.47E-05	3.90E-05	3.21E-05	3.02E-05	9.33E-06
pdb2b8i	77	5.60E-05	3.54E-05	2.78E-05	3.99E-05	1.55E-05
pdb2e3i	78	5.60E-05	3.63E-05	2.69E-05	3.02E-05	1.87E-05
pdb2j8b	78	6.25E-05	3.72E-05	3.12E-05	3.02E-05	1.55E-05
3K3V	79	5.60E-05	3.48E-05	2.78E-05	3.02E-05	1.55E-05
pdb3dkm	79	6.25E-05	3.63E-05	3.21E-05	3.02E-05	1.87E-05
1X3O	80	5.60E-05	3.45E-05	2.86E-05	3.02E-05	1.87E-05
2ZQE	80	6.03E-05	3.73E-05	2.87E-05	3.02E-05	1.85E-05
3RFI	80	5.18E-05	3.45E-05	2.69E-05	3.02E-05	1.85E-05
pdb1nh9	80	5.60E-05	3.54E-05	2.96E-05	1.03E-05	1.55E-05
pdb1zzk	80	5.60E-05	3.27E-05	2.78E-05	3.02E-05	2.16E-05
3LLB	81	6.25E-05	3.82E-05	3.04E-05	3.02E-05	1.87E-05
4F25	81	6.05E-05	3.63E-05	3.04E-05	3.02E-05	1.87E-05
pdb1tsf	81	6.90E-05	4.18E-05	3.30E-05	3.02E-05	1.87E-05
pdb2e3h	81	6.27E-05	3.91E-05	3.04E-05	3.02E-05	1.55E-05
pdb2o37	81	6.25E-05	3.90E-05	3.04E-05	2.96E-05	1.55E-05
pdb2pne	81	4.31E-05	3.00E-05	2.26E-05	3.02E-05	1.55E-05
1ZPW	82	5.95E-05	3.72E-05	3.04E-05	4.05E-05	1.87E-05
pdb1uln	82	6.05E-05	3.72E-05	2.95E-05	2.96E-05	1.87E-05
pdb2ckx	83	6.47E-05	3.73E-05	3.04E-05	3.99E-05	1.87E-05
pdb1zeq	84	5.60E-05	3.74E-05	2.86E-05	3.99E-05	1.87E-05
3KZD	85	6.03E-05	3.63E-05	2.87E-05	3.02E-05	1.87E-05
4IL7	85	6.47E-05	4.28E-05	3.21E-05	3.02E-05	1.55E-05
pdb2fq3	85	6.90E-05	4.09E-05	3.30E-05	2.06E-05	1.23E-05
2QVO	87	6.68E-05	4.27E-05	3.56E-05	3.02E-05	1.23E-05
pdb1ptf	87	6.68E-05	4.00E-05	3.13E-05	1.03E-05	1.85E-05
pdb1ulr	87	6.03E-05	4.18E-05	3.04E-05	3.02E-05	1.87E-05
pdb2i6v	87	6.05E-05	3.72E-05	3.21E-05	2.96E-05	1.87E-05
1VJK	88	7.34E-05	4.27E-05	3.30E-05	3.99E-05	1.87E-05

4HTI	88	6.25E-05	3.81E-05	3.13E-05	3.02E-05	1.87E-05
pdb1dsl	88	6.69E-05	3.99E-05	3.21E-05	3.02E-05	1.55E-05
pdb1tig	88	7.33E-05	4.36E-05	3.47E-05	3.02E-05	1.85E-05
pdb1x6j	88	6.68E-05	4.09E-05	3.21E-05	4.05E-05	1.55E-05
3FDR	89	7.33E-05	4.17E-05	3.74E-05	1.99E-05	1.87E-05
pdb2f15	89	6.46E-05	4.09E-05	3.22E-05	3.02E-05	1.87E-05
2Q9V	90	6.25E-05	4.09E-05	3.12E-05	3.02E-05	1.23E-05
2V75	90	6.69E-05	4.18E-05	3.30E-05	3.99E-05	1.87E-05
3MX7	90	6.90E-05	3.99E-05	3.30E-05	3.99E-05	1.87E-05
4GS3	90	6.47E-05	3.99E-05	3.04E-05	3.02E-05	1.87E-05
4OIX	90	6.47E-05	3.91E-05	3.21E-05	3.02E-05	1.55E-05
pdb2cg7	90	6.47E-05	3.82E-05	3.12E-05	2.96E-05	1.87E-05
pdb3ce7	90	7.11E-05	4.18E-05	3.39E-05	1.99E-05	2.18E-05
1R5Q	91	6.90E-05	4.09E-05	3.30E-05	3.02E-05	1.85E-05
2GZV	91	6.05E-05	4.00E-05	2.95E-05	3.99E-05	1.87E-05
4LTT	91	7.33E-05	4.36E-05	3.47E-05	3.02E-05	1.87E-05
pdb1b9w	91	6.69E-05	5.90E-05	3.30E-05	2.96E-05	1.25E-05
pdb2o71	91	7.33E-05	4.63E-05	3.56E-05	4.05E-05	1.87E-05
4GMQ	92	6.68E-05	4.55E-05	3.22E-05	3.02E-05	1.23E-05
pdb2ygs	92	6.69E-05	4.09E-05	3.30E-05	3.02E-05	1.87E-05
1MZL	93	5.82E-05	3.91E-05	2.87E-05	3.02E-05	1.87E-05
3ID4	93	6.05E-05	3.96E-05	3.21E-05	3.02E-05	1.55E-05
pdb2rb8	93	6.90E-05	4.63E-05	3.30E-05	4.05E-05	1.87E-05
2VH7	94	6.90E-05	4.27E-05	3.39E-05	3.99E-05	1.55E-05
3ID1	94	7.12E-05	4.36E-05	3.47E-05	3.02E-05	1.87E-05
3KP8	94	6.74E-05	4.18E-05	3.30E-05	1.03E-05	1.85E-05
3LMO	94	6.68E-05	4.45E-05	3.21E-05	3.02E-05	1.87E-05
4O7Q	94	6.90E-05	4.46E-05	3.30E-05	3.99E-05	1.87E-05
pdb1qzm	94	6.69E-05	4.54E-05	3.30E-05	3.02E-05	1.85E-05
3JU0	95	7.33E-05	4.54E-05	3.74E-05	2.06E-05	1.87E-05
3JVE	95	7.33E-05	4.27E-05	3.47E-05	2.06E-05	1.25E-05
pdb1lpl	95	6.46E-05	3.99E-05	3.21E-05	3.99E-05	1.87E-05
pdb2qt4	95	6.25E-05	4.27E-05	3.12E-05	3.02E-05	1.87E-05
2WJ5	96	6.68E-05	4.18E-05	3.21E-05	9.65E-06	1.87E-05
3RJP	96	6.90E-05	4.17E-05	3.30E-05	3.99E-05	1.87E-05
pdb1mwp	96	6.68E-05	4.72E-05	3.30E-05	3.02E-05	1.25E-05
pdb1t2i	96	6.68E-05	4.27E-05	3.30E-05	3.02E-05	1.87E-05
pdb1u9p	96	7.55E-05	4.73E-05	3.47E-05	3.02E-05	2.16E-05
pdb1z21	96	7.55E-05	4.55E-05	3.56E-05	2.96E-05	1.87E-05
pdb2ptv	96	7.54E-05	4.54E-05	3.65E-05	3.02E-05	1.85E-05
2BK8	97	6.68E-05	4.99E-05	3.30E-05	9.65E-06	1.87E-05

2CWR	97	6.69E-05	4.45E-05	3.30E-05	3.02E-05	1.87E-05
3ONJ	97	6.90E-05	4.45E-05	3.30E-05	3.99E-05	1.87E-05
pdb1lou	97	7.55E-05	4.45E-05	3.56E-05	2.96E-05	1.87E-05
1J27	98	7.55E-05	4.54E-05	3.56E-05	6.05E-05	1.87E-05
pdb1ln4	98	7.12E-05	4.45E-05	3.39E-05	3.02E-05	2.16E-05
pdb1bm8	99	8.63E-05	4.99E-05	4.09E-05	3.02E-05	1.87E-05
pdb1cqy	99	8.63E-05	4.54E-05	3.39E-05	4.05E-05	1.23E-05
pdb1opc	99	7.77E-05	4.54E-05	3.56E-05	4.05E-05	1.87E-05
pdb2j9v	99	7.12E-05	4.73E-05	3.47E-05	1.99E-05	1.87E-05
pdb2pcy	99	6.69E-05	4.17E-05	3.30E-05	3.02E-05	1.85E-05
pdb2pko	99	7.12E-05	4.18E-05	3.39E-05	3.99E-05	1.85E-05
pdb2yxf	99	7.77E-05	4.91E-05	3.65E-05	9.65E-06	9.33E-06
pdb1eoe	100	7.54E-05	4.72E-05	3.56E-05	3.02E-05	1.57E-05
pdb1jos	100	7.12E-05	4.47E-05	3.39E-05	3.02E-05	1.87E-05
pdb1w41	100	7.33E-05	4.73E-05	3.47E-05	3.02E-05	1.85E-05
2PE8	101	6.92E-05	4.91E-05	3.47E-05	2.96E-05	1.85E-05
3BZT	101	7.77E-05	4.36E-05	3.39E-05	3.02E-05	1.87E-05
1P1L	102	7.11E-05	4.54E-05	3.56E-05	3.99E-05	2.16E-05
3ADY	102	7.55E-05	4.63E-05	3.65E-05	3.99E-05	1.87E-05
3EAZ	102	8.20E-05	4.90E-05	3.74E-05	3.02E-05	1.87E-05
3ISU	102	7.98E-05	6.72E-05	3.82E-05	4.05E-05	1.87E-05
3KT9	102	7.76E-05	4.73E-05	3.73E-05	3.02E-05	1.87E-05
4DOT	102	7.99E-05	5.00E-05	3.83E-05	4.05E-05	1.87E-05
pdb1fhg	102	8.20E-05	4.99E-05	3.82E-05	4.05E-05	1.87E-05
3HAK	103	7.55E-05	4.63E-05	3.65E-05	3.02E-05	1.87E-05
2FD4	104	7.81E-05	4.81E-05	3.66E-05	3.99E-05	1.87E-05
2X5P	104	8.18E-05	6.63E-05	3.83E-05	4.05E-05	1.85E-05
3VFI	104	8.63E-05	5.18E-05	4.09E-05	2.06E-05	2.48E-05
1WWC	105	7.54E-05	4.73E-05	3.65E-05	3.99E-05	1.85E-05
3AG7	105	7.77E-05	5.01E-05	3.74E-05	1.99E-05	1.23E-05
pdb1aaj	105	7.38E-05	4.81E-05	3.65E-05	3.34E-05	2.48E-05
pdb1m5i	105	7.99E-05	4.70E-05	3.74E-05	3.02E-05	1.87E-05
2VQ4	106	8.41E-05	5.18E-05	3.91E-05	3.02E-05	1.87E-05
3A4C	106	7.33E-05	4.99E-05	3.56E-05	3.02E-05	2.16E-05
4EO0	106	7.77E-05	4.63E-05	3.65E-05	4.05E-05	1.87E-05
4GGR	106	7.55E-05	4.99E-05	3.56E-05	4.05E-05	1.87E-05
pdb1ew4	106	7.77E-05	4.73E-05	3.74E-05	3.02E-05	1.25E-05
pdb2frg	106	7.12E-05	4.63E-05	3.47E-05	3.02E-05	2.18E-05
2PPN	107	7.33E-05	4.81E-05	3.65E-05	3.02E-05	1.87E-05
3DJ9	107	7.55E-05	4.99E-05	3.56E-05	4.05E-05	1.25E-05
pdb1wpa	107	7.55E-05	4.81E-05	3.65E-05	1.03E-05	1.87E-05

pdb1xaw	107	7.76E-05	4.99E-05	3.65E-05	3.02E-05	1.87E-05
pdb3cx2	107	7.98E-05	5.18E-05	3.82E-05	3.99E-05	2.48E-05
3HNX	108	8.41E-05	5.09E-05	4.00E-05	3.02E-05	1.85E-05
3VVV	108	8.85E-05	6.99E-05	4.05E-05	1.99E-05	1.87E-05
3W56	108	8.41E-05	5.18E-05	3.91E-05	3.99E-05	1.23E-05
pdb1bkr	108	8.63E-05	5.17E-05	4.06E-05	3.02E-05	1.85E-05
pdb2i1u	108	7.55E-05	5.18E-05	3.74E-05	3.02E-05	1.87E-05
2FO3	109	8.63E-05	5.36E-05	4.09E-05	9.65E-06	1.87E-05
2R2Y	109	8.42E-05	5.55E-05	4.08E-05	1.99E-05	2.80E-05
1TQ3	110	8.20E-05	5.18E-05	3.78E-05	4.05E-05	1.87E-05
4HQA	110	8.41E-05	7.17E-05	4.00E-05	3.02E-05	1.87E-05
2WWE	111	8.20E-05	5.00E-05	3.74E-05	3.99E-05	1.87E-05
4MZ2	111	8.41E-05	5.36E-05	4.09E-05	4.05E-05	1.85E-05
pdb1roa	111	7.98E-05	5.01E-05	3.82E-05	3.99E-05	2.48E-05
pdb1qau	112	7.33E-05	4.72E-05	3.56E-05	3.02E-05	1.23E-05
pdb2og3	112	9.28E-05	7.27E-05	4.26E-05	3.02E-05	2.16E-05
3FKC	113	7.76E-05	5.27E-05	3.74E-05	4.05E-05	1.87E-05
3ONH	113	9.28E-05	5.55E-05	4.26E-05	4.05E-05	1.87E-05
4B50	113	8.20E-05	5.17E-05	3.91E-05	4.05E-05	1.55E-05
pdb1noa	113	7.12E-05	4.73E-05	3.47E-05	3.99E-05	1.25E-05
pdb2hc8	113	7.55E-05	6.90E-05	3.65E-05	1.99E-05	1.87E-05
1SAU	114	8.63E-05	5.55E-05	4.08E-05	3.99E-05	1.87E-05
3IU5	114	9.05E-05	5.81E-05	4.17E-05	3.99E-05	2.16E-05
pdb2o0q	114	7.98E-05	5.27E-05	3.82E-05	3.02E-05	2.80E-05
3EOD	115	8.42E-05	5.27E-05	4.08E-05	2.96E-05	1.87E-05
3K1H	115	9.05E-05	5.54E-05	4.26E-05	2.96E-05	3.71E-05
3LS0	115	8.63E-05	5.27E-05	4.08E-05	3.99E-05	2.48E-05
pdb2e7v	115	8.42E-05	5.36E-05	4.00E-05	3.99E-05	1.87E-05
1NPU	116	1.08E-04	7.17E-05	5.47E-05	5.08E-05	3.10E-05
pdb1bea	116	8.18E-05	5.27E-05	3.91E-05	4.05E-05	1.87E-05
pdb1jpe	116	9.05E-05	5.45E-05	4.17E-05	3.99E-05	1.87E-05
pdb1xte	116	9.10E-05	5.63E-05	4.26E-05	3.02E-05	1.87E-05
pdb2z14	116	8.83E-05	7.17E-05	4.26E-05	4.05E-05	2.18E-05
3L78	117	9.05E-05	5.63E-05	4.26E-05	4.05E-05	1.85E-05
3ZK0	117	8.63E-05	5.81E-05	4.08E-05	3.99E-05	2.16E-05
4I6X	117	8.85E-05	5.81E-05	4.25E-05	3.99E-05	1.87E-05
2FI9	118	8.41E-05	8.27E-05	3.91E-05	3.02E-05	1.87E-05
3RVC	118	8.63E-05	5.63E-05	4.08E-05	4.05E-05	1.55E-05
pdb1r9h	118	8.85E-05	5.63E-05	4.18E-05	4.05E-05	1.55E-05
1UNP	119	8.85E-05	7.45E-05	4.17E-05	1.03E-05	1.25E-05
3S0A	119	8.41E-05	5.36E-05	4.00E-05	1.99E-05	1.87E-05

pdb1wou	119	8.85E-05	5.73E-05	4.17E-05	3.99E-05	1.87E-05
pdb2pnd	119	8.63E-05	5.81E-05	4.17E-05	2.96E-05	2.16E-05
2WZ9	120	9.07E-05	6.09E-05	4.26E-05	3.99E-05	1.85E-05
3LF9	120	9.05E-05	5.81E-05	4.26E-05	4.05E-05	1.25E-05
4GCO	120	8.85E-05	5.63E-05	4.17E-05	3.99E-05	1.87E-05
pdb1gyu	120	8.41E-05	5.54E-05	4.00E-05	4.05E-05	1.25E-05
pdb1gyv	120	8.41E-05	5.45E-05	4.09E-05	3.99E-05	1.25E-05
pdb2fj8	120	8.18E-05	5.81E-05	4.08E-05	4.05E-05	1.87E-05
2RH3	121	8.41E-05	5.63E-05	4.00E-05	2.96E-05	2.18E-05
3SUL	121	8.41E-05	5.63E-05	4.08E-05	9.65E-06	1.85E-05
pdb1l6p	121	9.92E-05	6.27E-05	4.61E-05	3.99E-05	1.87E-05
pdb2grc	121	9.70E-05	6.27E-05	4.43E-05	3.02E-05	2.18E-05
pdb2qvk	121	9.28E-05	5.91E-05	4.43E-05	3.02E-05	1.87E-05
pdb3csp	121	9.28E-05	9.17E-05	4.34E-05	3.99E-05	1.57E-05
2GKG	122	9.50E-05	7.81E-05	4.43E-05	3.99E-05	1.87E-05
3T3K	122	9.28E-05	5.81E-05	4.34E-05	3.02E-05	2.16E-05
pdb1nko	122	9.70E-05	9.63E-05	4.51E-05	3.99E-05	1.87E-05
pdb1pzc	122	8.83E-05	5.91E-05	4.17E-05	3.99E-05	2.18E-05
pdb1zlb	122	8.85E-05	5.82E-05	4.17E-05	3.02E-05	1.87E-05
pdb2fwg	122	9.05E-05	5.81E-05	4.26E-05	2.96E-05	2.16E-05
3P6J	123	9.50E-05	6.36E-05	4.60E-05	2.06E-05	1.85E-05
3RNV	123	8.83E-05	5.72E-05	4.17E-05	3.02E-05	1.87E-05
pdb1c44	123	8.85E-05	7.54E-05	4.27E-05	2.96E-05	1.87E-05
pdb1huf	123	9.05E-05	6.09E-05	4.26E-05	4.05E-05	1.87E-05
pdb2ciu	123	8.85E-05	5.90E-05	4.17E-05	4.05E-05	2.16E-05
2FC3	124	9.05E-05	6.18E-05	4.25E-05	3.99E-05	2.16E-05
4DFI	124	8.63E-05	6.09E-05	4.08E-05	3.99E-05	2.18E-05
pdb1kf5	124	8.63E-05	5.63E-05	4.08E-05	2.96E-05	2.16E-05
2F6E	125	9.72E-05	6.27E-05	4.51E-05	3.02E-05	1.55E-05
2X35	125	9.28E-05	6.09E-05	4.45E-05	3.99E-05	1.25E-05
4B9I	125	8.85E-05	5.83E-05	4.25E-05	3.99E-05	1.87E-05
pdb1acf	125	8.63E-05	5.63E-05	4.09E-05	3.67E-05	2.48E-05
pdb1bfg	126	1.04E-04	6.54E-05	4.78E-05	3.99E-05	2.16E-05
pdb1tp6	126	8.83E-05	5.99E-05	4.17E-05	3.02E-05	1.87E-05
pdb1f32	127	9.28E-05	6.09E-05	4.36E-05	1.99E-05	2.18E-05
pdb1jb3	127	9.28E-05	5.99E-05	4.43E-05	1.99E-05	1.87E-05
3A7L	128	8.85E-05	5.91E-05	4.17E-05	9.65E-06	1.87E-05
3O5E	128	8.85E-05	6.10E-05	4.17E-05	3.02E-05	2.16E-05
3O5P	128	8.85E-05	5.81E-05	4.17E-05	4.05E-05	2.16E-05
2W0G	129	1.04E-04	6.54E-05	4.88E-05	3.02E-05	1.55E-05
3NE3	129	9.05E-05	5.91E-05	4.34E-05	1.99E-05	1.87E-05

3O48	129	9.28E-05	6.18E-05	4.43E-05	3.99E-05	1.25E-05
pdb1gp3	129	9.28E-05	8.17E-05	4.43E-05	3.02E-05	1.87E-05
pdb1j3a	129	9.50E-05	6.45E-05	4.34E-05	3.99E-05	2.16E-05
pdb1lsy	129	9.28E-05	6.17E-05	4.43E-05	4.05E-05	1.85E-05
2D4P	130	9.52E-05	5.99E-05	4.34E-05	3.02E-05	2.48E-05
3HD4	131	1.04E-04	8.36E-05	4.86E-05	1.03E-05	1.23E-05
3NPH	131	1.10E-04	6.81E-05	5.13E-05	4.05E-05	2.16E-05
pdb1lit	131	9.70E-05	6.35E-05	4.86E-05	3.02E-05	1.23E-05
pdb1lmi	131	8.63E-05	5.99E-05	4.17E-05	3.02E-05	2.16E-05
pdb1qgv	131	1.06E-04	8.36E-05	4.86E-05	4.05E-05	2.16E-05
pdb1t3y	131	1.06E-04	6.99E-05	4.95E-05	3.99E-05	2.16E-05
pdb2lis	131	1.01E-04	6.63E-05	4.78E-05	3.02E-05	1.55E-05
1WVH	132	9.28E-05	6.36E-05	4.43E-05	4.05E-05	1.25E-05
3CO1	132	1.06E-04	6.81E-05	4.86E-05	3.99E-05	1.39E-05
3S60	132	8.83E-05	6.09E-05	4.26E-05	3.99E-05	1.25E-05
3TIP	132	9.05E-05	5.99E-05	4.26E-05	4.05E-05	2.18E-05
pdb1kt9	132	1.08E-04	7.08E-05	4.95E-05	3.99E-05	1.55E-05
pdb1nfn	132	1.04E-04	8.36E-05	4.69E-05	4.05E-05	2.16E-05
2EIF	133	1.08E-04	6.91E-05	5.04E-05	3.99E-05	3.71E-05
2XGV	133	1.06E-04	6.72E-05	4.95E-05	4.05E-05	1.85E-05
pdb1klx	133	1.04E-04	6.90E-05	4.69E-05	3.99E-05	2.16E-05
1ZHV	134	8.85E-05	8.27E-05	4.34E-05	3.99E-05	1.87E-05
2EJX	134	9.92E-05	6.45E-05	4.60E-05	9.65E-06	1.87E-05
2FZP	134	1.01E-04	6.63E-05	4.70E-05	4.05E-05	2.18E-05
pdb1lu4	134	1.14E-04	7.27E-05	5.30E-05	4.05E-05	1.85E-05
pdb2yvq	134	9.92E-05	6.72E-05	4.69E-05	4.05E-05	2.16E-05
2W0I	135	9.92E-05	6.45E-05	4.60E-05	3.99E-05	1.85E-05
3FLG	135	1.08E-04	6.99E-05	5.04E-05	4.05E-05	1.87E-05
4LJ1	135	9.05E-05	6.45E-05	4.43E-05	3.02E-05	2.18E-05
1R62	136	9.92E-05	6.54E-05	4.61E-05	1.99E-05	2.18E-05
3VBC	136	1.04E-04	6.91E-05	4.86E-05	3.99E-05	1.55E-05
pdb1ey4	136	1.04E-04	6.90E-05	4.69E-05	3.99E-05	2.18E-05
pdb1xgw	136	1.12E-04	7.09E-05	5.21E-05	9.65E-06	1.55E-05
pdb2f1y	136	1.10E-04	6.90E-05	5.05E-05	3.02E-05	2.16E-05
pdb2vo8	136	9.70E-05	6.73E-05	4.60E-05	3.02E-05	2.16E-05
1IFG	137	9.72E-05	6.45E-05	5.05E-05	4.05E-05	2.48E-05
3K8U	137	1.06E-04	6.90E-05	5.04E-05	1.99E-05	2.16E-05
3RDJ	137	1.01E-04	6.72E-05	4.78E-05	4.05E-05	2.16E-05
pdb1gak	137	1.12E-04	7.36E-05	5.21E-05	4.05E-05	1.85E-05
pdb2end	137	1.04E-04	6.63E-05	4.69E-05	4.05E-05	2.16E-05
2CXC	138	9.48E-05	6.90E-05	4.52E-05	4.05E-05	2.18E-05

2JHY	138	1.06E-04	7.27E-05	4.78E-05	4.05E-05	1.87E-05
3ZSL	138	1.01E-04	7.09E-05	4.71E-05	4.05E-05	1.87E-05
4EVM	138	1.01E-04	6.90E-05	4.78E-05	3.99E-05	1.87E-05
4NI6	138	1.04E-04	7.17E-05	5.22E-05	4.05E-05	1.87E-05
pdb1i2h	138	1.06E-04	7.00E-05	4.95E-05	1.99E-05	2.16E-05
pdb1r9w	138	9.92E-05	6.91E-05	4.60E-05	3.99E-05	2.16E-05
2A4D	139	1.10E-04	7.17E-05	5.04E-05	3.02E-05	2.16E-05
3O7K	139	9.70E-05	6.54E-05	4.52E-05	3.99E-05	1.55E-05
3RZY	139	9.32E-05	6.17E-05	4.43E-05	3.02E-05	1.87E-05
pdb1j74	139	9.91E-05	6.91E-05	4.69E-05	3.02E-05	2.16E-05
pdb2in0	139	1.01E-04	6.81E-05	4.69E-05	1.99E-05	2.16E-05
pdb2nrr	139	1.12E-04	7.72E-05	5.21E-05	3.99E-05	2.16E-05
2FK9	140	1.01E-04	6.54E-05	4.69E-05	3.99E-05	1.25E-05
3OBS	140	1.06E-04	7.09E-05	4.95E-05	3.99E-05	2.16E-05
4E9E	140	1.16E-04	7.36E-05	5.22E-05	4.05E-05	1.87E-05
4G08	140	1.12E-04	9.08E-05	5.22E-05	3.99E-05	2.16E-05
pdb1p4p	140	9.48E-05	6.72E-05	4.43E-05	4.05E-05	2.16E-05
pdb1q2y	140	9.92E-05	6.72E-05	4.69E-05	2.06E-05	2.16E-05
pdb1rss	140	9.70E-05	6.72E-05	4.61E-05	4.05E-05	1.85E-05
1OZ9	141	1.10E-04	7.17E-05	5.13E-05	1.16E-05	2.16E-05
1TZV	141	1.14E-04	7.72E-05	5.13E-05	3.99E-05	1.85E-05
2D59	141	1.06E-04	7.09E-05	4.86E-05	4.05E-05	2.16E-05
4H9J	141	1.04E-04	7.45E-05	4.86E-05	3.99E-05	1.85E-05
3DFG	142	1.01E-04	7.09E-05	4.78E-05	3.99E-05	1.87E-05
pdb1k6k	142	1.19E-04	7.81E-05	5.47E-05	3.99E-05	2.48E-05
pdb1wka	143	1.10E-04	7.72E-05	5.14E-05	4.05E-05	2.18E-05
1BZ4	144	1.14E-04	7.45E-05	5.82E-05	8.04E-05	3.73E-05
1EYH	144	1.08E-04	7.27E-05	4.95E-05	5.02E-05	2.18E-05
1KNG	144	1.16E-04	7.91E-05	5.47E-05	3.99E-05	3.10E-05
3ILC	144	1.06E-04	7.35E-05	4.86E-05	4.05E-05	2.16E-05
4GEI	144	1.01E-04	6.99E-05	4.69E-05	3.02E-05	1.87E-05
pdb1gs9	144	1.08E-04	8.99E-05	4.86E-05	3.99E-05	2.16E-05
pdb1l2h	144	1.08E-04	7.81E-05	5.04E-05	5.02E-05	2.18E-05
pdb1o8x	144	1.14E-04	7.72E-05	5.21E-05	4.05E-05	2.18E-05
2QEV	145	1.08E-04	7.81E-05	5.13E-05	4.05E-05	1.87E-05
3MM4	145	1.12E-04	7.45E-05	5.12E-05	2.06E-05	2.18E-05
pdb1q5z	145	1.10E-04	7.91E-05	5.13E-05	4.05E-05	2.18E-05
pdb1srv	145	1.04E-04	7.27E-05	4.86E-05	2.06E-05	2.16E-05
pdb2p5d	145	1.10E-04	7.36E-05	5.13E-05	3.02E-05	2.18E-05
1NIG	146	1.23E-04	8.35E-05	5.56E-05	4.05E-05	2.16E-05
3FH2	146	1.06E-04	7.36E-05	5.04E-05	4.05E-05	2.16E-05

pdb1jmw	146	1.01E-04	7.08E-05	4.78E-05	4.05E-05	2.16E-05
1O6D	147	1.16E-04	8.17E-05	5.39E-05	4.05E-05	2.16E-05
2QPW	147	1.04E-04	7.27E-05	5.74E-05	4.05E-05	2.16E-05
4G78	147	1.08E-04	7.45E-05	5.04E-05	4.05E-05	2.16E-05
pdb1khi	147	1.06E-04	8.17E-05	4.95E-05	3.99E-05	2.16E-05
3G9B	148	1.14E-04	7.81E-05	5.30E-05	3.99E-05	2.16E-05
3JZZ	148	9.92E-05	7.09E-05	4.61E-05	3.99E-05	2.16E-05
pdb1ng6	148	1.01E-04	7.63E-05	4.86E-05	3.02E-05	2.16E-05
pdb1rj1	148	1.10E-04	7.81E-05	5.13E-05	3.99E-05	2.18E-05
2ESK	149	1.08E-04	7.45E-05	5.13E-05	4.05E-05	2.16E-05
2Y9F	149	1.16E-04	7.91E-05	5.39E-05	3.99E-05	2.16E-05
3A0X	149	1.14E-04	7.81E-05	5.39E-05	2.06E-05	1.87E-05
pdb1x91	149	1.08E-04	7.63E-05	5.05E-05	3.99E-05	1.25E-05
pdb2b1k	149	1.16E-04	7.81E-05	5.39E-05	4.05E-05	1.55E-05
2OP6	150	1.06E-04	7.45E-05	4.86E-05	3.02E-05	2.18E-05
3N0K	150	1.08E-04	7.63E-05	5.12E-05	3.99E-05	1.55E-05
3PR9	150	1.08E-04	7.92E-05	5.04E-05	4.05E-05	2.18E-05
pdb1amx	150	1.23E-04	1.18E-04	5.82E-05	1.99E-05	2.16E-05
pdb1bj7	150	1.10E-04	7.81E-05	5.13E-05	4.05E-05	2.16E-05
pdb2jcp	150	1.19E-04	7.99E-05	5.39E-05	4.05E-05	2.18E-05
pdb2ywn	150	1.21E-04	7.36E-05	5.13E-05	1.99E-05	2.16E-05
1ZUH	151	1.14E-04	7.90E-05	5.30E-05	3.99E-05	2.18E-05
3SZ7	151	1.10E-04	9.63E-05	5.22E-05	3.99E-05	2.16E-05
pdb2q5x	151	1.08E-04	7.54E-05	5.04E-05	6.05E-05	2.16E-05
2OEB	152	1.21E-04	8.09E-05	5.47E-05	4.05E-05	1.55E-05
2VY8	152	1.06E-04	7.63E-05	4.95E-05	4.05E-05	2.18E-05
4AGK	152	1.06E-04	7.54E-05	4.97E-05	4.05E-05	1.57E-05
pdb1dvo	152	1.04E-04	7.63E-05	4.86E-05	1.99E-05	2.16E-05
pdb1jl1	152	1.19E-04	8.63E-05	5.48E-05	2.06E-05	1.23E-05
pdb1hzt	153	1.19E-04	8.36E-05	5.47E-05	3.99E-05	2.16E-05
pdb3eye	153	1.16E-04	8.09E-05	5.39E-05	3.99E-05	2.16E-05
3PIW	154	1.19E-04	8.09E-05	5.39E-05	4.05E-05	2.18E-05
pdb2pwq	154	1.12E-04	7.81E-05	5.22E-05	3.99E-05	2.16E-05
2CZT	155	1.12E-04	7.91E-05	5.21E-05	4.05E-05	2.16E-05
3Q6B	155	1.12E-04	8.36E-05	5.30E-05	3.99E-05	2.18E-05
pdb1icx	155	1.08E-04	7.99E-05	5.04E-05	4.05E-05	2.16E-05
pdb1jyh	155	1.23E-04	8.72E-05	5.64E-05	3.99E-05	2.48E-05
pdb2rer	155	1.21E-04	8.27E-05	5.73E-05	4.05E-05	2.48E-05
3HNY	156	1.23E-04	8.54E-05	5.73E-05	1.99E-05	2.16E-05
3ML3	156	1.27E-04	8.63E-05	5.90E-05	3.99E-05	2.16E-05
3KH7	157	1.21E-04	8.81E-05	5.65E-05	1.99E-05	2.18E-05

3MEW	157	1.16E-04	8.54E-05	5.39E-05	3.99E-05	2.16E-05
3NR5	157	1.14E-04	8.18E-05	5.39E-05	3.99E-05	2.16E-05
3V1Q	157	1.21E-04	8.63E-05	5.56E-05	3.99E-05	1.55E-05
4J8Y	157	1.10E-04	9.81E-05	5.13E-05	4.05E-05	2.16E-05
pdb1tol	157	1.19E-04	8.63E-05	5.56E-05	4.05E-05	2.18E-05
pdb1txj	157	1.16E-04	1.05E-04	5.40E-05	4.05E-05	2.16E-05
4ESS	158	1.14E-04	8.72E-05	5.30E-05	4.05E-05	2.16E-05
pdb1bgc	158	1.36E-04	9.35E-05	6.17E-05	3.99E-05	2.16E-05
pdb1hka	158	1.14E-04	8.72E-05	5.30E-05	4.05E-05	2.48E-05
1Q42	159	1.21E-04	8.54E-05	5.56E-05	3.99E-05	2.18E-05
3CSR	159	1.23E-04	8.72E-05	5.65E-05	3.99E-05	2.16E-05
3IXR	159	1.25E-04	1.05E-04	5.99E-05	3.99E-05	1.55E-05
pdb1t5i	159	1.19E-04	8.63E-05	5.48E-05	3.99E-05	2.18E-05
4DT4	160	1.06E-04	7.72E-05	4.69E-05	4.05E-05	2.16E-05
pdb1jvw	160	1.34E-04	8.81E-05	5.73E-05	3.99E-05	1.55E-05
1K95	161	1.21E-04	8.63E-05	5.48E-05	5.02E-05	2.16E-05
1Z7C	161	1.32E-04	9.27E-05	6.00E-05	4.05E-05	2.18E-05
2FR2	161	1.27E-04	8.90E-05	5.82E-05	4.05E-05	2.16E-05
3HA9	161	1.32E-04	9.09E-05	5.99E-05	1.99E-05	1.55E-05
4CHE	161	1.32E-04	8.99E-05	6.10E-05	4.05E-05	1.55E-05
2DYI	162	1.12E-04	8.46E-05	5.21E-05	4.05E-05	2.16E-05
pdb1l3k	163	1.29E-04	9.45E-05	5.90E-05	3.99E-05	1.55E-05
pdb1fl0	164	1.27E-04	9.90E-05	5.82E-05	4.05E-05	2.18E-05
pdb1p7s	164	1.19E-04	8.81E-05	5.48E-05	4.95E-05	2.16E-05
pdb1rl6	164	1.21E-04	8.63E-05	5.56E-05	3.99E-05	1.55E-05
pdb2ova	164	1.25E-04	8.99E-05	5.82E-05	3.99E-05	2.48E-05
2FPH	165	1.27E-04	9.45E-05	5.91E-05	4.05E-05	2.16E-05
3EJG	165	1.23E-04	9.09E-05	5.73E-05	3.99E-05	2.16E-05
3G39	165	1.25E-04	9.45E-05	5.82E-05	3.99E-05	2.16E-05
pdb1kxo	165	1.29E-04	9.18E-05	5.92E-05	3.99E-05	2.16E-05
pdb1s21	165	1.16E-04	1.20E-04	5.40E-05	3.99E-05	2.48E-05
pdb2hwx	165	1.29E-04	9.17E-05	5.91E-05	5.02E-05	2.48E-05
pdb2obi	165	1.23E-04	8.99E-05	5.99E-05	3.99E-05	2.16E-05
pdb3bci	165	1.34E-04	9.27E-05	6.17E-05	3.99E-05	2.16E-05
2X3M	166	1.36E-04	9.63E-05	6.17E-05	3.99E-05	1.55E-05
3EJF	166	1.27E-04	9.36E-05	5.99E-05	5.02E-05	1.57E-05
4A02	166	1.25E-04	9.45E-05	5.82E-05	1.99E-05	2.16E-05
pdb1qnt	166	1.16E-04	8.72E-05	5.47E-05	4.05E-05	2.16E-05
4ACJ	167	1.40E-04	9.90E-05	6.60E-05	4.05E-05	2.16E-05
pdb1ow1	167	1.16E-04	8.90E-05	5.39E-05	1.99E-05	2.48E-05
pdb1pgv	167	1.34E-04	9.53E-05	6.17E-05	4.05E-05	2.18E-05

pdb3d79	167	1.34E-04	9.27E-05	6.17E-05	3.99E-05	2.18E-05
3H6Q	168	1.23E-04	1.24E-04	5.74E-05	4.05E-05	2.18E-05
4E2U	168	1.23E-04	8.96E-05	5.65E-05	4.05E-05	2.18E-05
4JQF	168	1.27E-04	9.27E-05	5.91E-05	3.99E-05	2.16E-05
pdb1nwa	168	1.36E-04	9.54E-05	6.08E-05	3.99E-05	2.16E-05
pdb2rkq	168	1.23E-04	9.54E-05	5.73E-05	5.02E-05	1.55E-05
pdb2sga	168	1.21E-04	9.09E-05	5.64E-05	3.99E-05	2.16E-05
3BQE	169	1.36E-04	9.63E-05	6.08E-05	4.05E-05	2.48E-05
3WI0	169	1.32E-04	9.90E-05	6.17E-05	4.05E-05	2.18E-05
pdb1v7q	169	1.32E-04	1.01E-04	6.08E-05	2.06E-05	2.48E-05
pdb3eo5	169	1.25E-04	1.18E-04	5.82E-05	3.99E-05	1.87E-05
4BXP	170	1.16E-04	9.17E-05	5.39E-05	4.05E-05	2.18E-05
pdb1o9z	170	1.23E-04	9.63E-05	5.65E-05	3.99E-05	1.55E-05
pdb1oxj	170	1.32E-04	9.72E-05	6.17E-05	5.02E-05	1.57E-05
2X5Y	171	1.32E-04	1.01E-04	6.08E-05	3.99E-05	2.18E-05
3RT2	171	1.29E-04	9.64E-05	5.99E-05	4.05E-05	2.18E-05
pdb1qzn	171	1.19E-04	9.17E-05	5.47E-05	5.02E-05	2.16E-05
pdb1wba	171	1.36E-04	1.01E-04	6.25E-05	5.02E-05	2.18E-05
pdb1koe	172	1.27E-04	9.72E-05	5.90E-05	5.02E-05	1.57E-05
pdb1lki	172	1.21E-04	9.46E-05	5.65E-05	3.99E-05	2.18E-05
3PM2	173	1.19E-04	9.45E-05	5.56E-05	4.05E-05	2.48E-05
pdb1h4a	173	1.38E-04	1.03E-04	6.25E-05	3.99E-05	2.16E-05
pdb2fcb	173	1.25E-04	9.45E-05	5.73E-05	4.05E-05	2.48E-05
1NG2	176	1.32E-04	1.04E-04	5.91E-05	6.05E-05	2.16E-05
2ZTY	176	1.42E-04	1.03E-04	6.43E-05	3.99E-05	2.48E-05
4ANN	176	1.38E-04	1.05E-04	6.86E-05	4.05E-05	2.18E-05
pdb1sqw	176	1.36E-04	1.00E-04	6.17E-05	1.99E-05	2.48E-05
3FN7	177	1.32E-04	9.90E-05	6.00E-05	4.05E-05	2.18E-05
3KSN	177	1.29E-04	1.04E-04	5.99E-05	3.99E-05	2.48E-05
pdb1hbq	177	1.40E-04	1.05E-04	6.35E-05	3.99E-05	2.18E-05
pdb1yw5	177	1.27E-04	9.90E-05	5.91E-05	5.02E-05	1.55E-05
pdb2f1s	177	1.40E-04	1.04E-04	6.52E-05	4.05E-05	2.48E-05
1CDY	178	1.43E-04	1.11E-04	6.69E-05	8.04E-05	4.03E-05
2OIX	178	1.38E-04	1.04E-04	6.34E-05	4.05E-05	2.48E-05
pdb1mjs	178	1.34E-04	1.06E-04	6.34E-05	5.02E-05	2.48E-05
pdb2i5h	178	1.58E-04	1.12E-04	7.21E-05	3.99E-05	2.18E-05
pdb2nx2	178	1.42E-04	1.04E-04	6.43E-05	3.02E-05	2.48E-05
pdb2hp7	179	1.36E-04	1.03E-04	6.25E-05	4.05E-05	2.48E-05
pdb2iu1	179	1.49E-04	1.08E-04	6.78E-05	5.02E-05	2.78E-05
pdb3d2a	179	1.29E-04	1.04E-04	6.17E-05	1.03E-05	1.87E-05
1KN3	180	1.42E-04	1.12E-04	6.52E-05	2.06E-05	2.18E-05

3ICH	180	1.36E-04	1.04E-04	6.34E-05	5.02E-05	2.16E-05
3UC9	180	1.45E-04	1.25E-04	6.52E-05	4.05E-05	1.85E-05
pdb3cou	180	1.36E-04	1.03E-04	6.25E-05	1.99E-05	1.57E-05
1YTQ	181	1.40E-04	1.05E-04	6.43E-05	4.05E-05	2.16E-05
3EIZ	182	1.25E-04	9.63E-05	5.73E-05	4.05E-05	2.16E-05
4LEU	182	1.47E-04	1.10E-04	6.78E-05	4.05E-05	2.16E-05
4N30	182	1.38E-04	1.07E-04	6.25E-05	3.99E-05	2.16E-05
pdb1m1h	182	1.38E-04	1.07E-04	6.34E-05	3.99E-05	2.48E-05
2FL7	183	1.49E-04	1.11E-04	6.78E-05	5.02E-05	2.16E-05
3UV9	183	1.36E-04	1.17E-04	6.34E-05	3.99E-05	2.48E-05
2P8T	184	1.45E-04	1.15E-04	6.60E-05	3.99E-05	1.55E-05
3KG4	184	1.57E-04	1.17E-04	7.04E-05	3.99E-05	2.48E-05
2E8B	185	1.57E-04	1.17E-04	7.12E-05	4.05E-05	2.18E-05
2YWJ	185	1.47E-04	1.10E-04	6.69E-05	3.99E-05	2.18E-05
3FZE	185	1.70E-04	1.22E-04	7.64E-05	3.99E-05	2.48E-05
4JG2	185	1.32E-04	1.03E-04	6.08E-05	4.05E-05	2.18E-05
pdb1gbs	185	1.38E-04	1.11E-04	6.25E-05	2.06E-05	2.48E-05
pdb2p65	185	1.32E-04	1.05E-04	6.08E-05	1.99E-05	2.48E-05
1P5F	186	1.38E-04	1.12E-04	6.34E-05	3.99E-05	2.48E-05
3FRR	186	1.45E-04	1.10E-04	6.52E-05	4.05E-05	2.48E-05
3ETP	187	1.45E-04	1.10E-04	6.52E-05	4.05E-05	2.48E-05
2D7J	188	1.62E-04	1.21E-04	7.29E-05	4.05E-05	2.48E-05
3ERB	188	1.40E-04	1.13E-04	6.52E-05	3.99E-05	2.18E-05
pdb1jhs	188	1.36E-04	1.10E-04	6.25E-05	3.99E-05	2.48E-05
pdb1ukf	188	1.55E-04	1.21E-04	7.14E-05	3.99E-05	2.48E-05
pdb1wj9	188	1.36E-04	1.13E-04	6.34E-05	5.02E-05	2.16E-05
pdb2rci	188	1.57E-04	1.18E-04	7.12E-05	5.02E-05	2.18E-05
pdb1rgp	189	1.53E-04	1.19E-04	7.13E-05	3.99E-05	2.48E-05
pdb1tua	189	1.41E-04	1.12E-04	6.43E-05	4.05E-05	2.46E-05
2R77	190	1.47E-04	1.23E-04	6.61E-05	4.05E-05	2.78E-05
3A2Z	190	1.40E-04	1.14E-04	6.52E-05	1.99E-05	2.48E-05
3OSX	190	1.36E-04	1.14E-04	6.25E-05	1.99E-05	2.48E-05
pdb1iap	190	1.53E-04	1.18E-04	6.95E-05	3.99E-05	2.18E-05
pdb3tss	190	1.45E-04	1.16E-04	6.60E-05	3.99E-05	2.48E-05
3LTJ	191	1.49E-04	1.17E-04	6.78E-05	6.05E-05	1.57E-05
2AP3	192	1.49E-04	1.16E-04	6.69E-05	5.02E-05	2.48E-05
3HZ8	192	1.64E-04	1.24E-04	7.38E-05	1.99E-05	2.48E-05
4JMI	192	1.60E-04	1.22E-04	7.21E-05	4.05E-05	2.48E-05
2PTH	193	1.40E-04	1.24E-04	6.60E-05	4.05E-05	2.16E-05
3KB5	193	1.42E-04	1.17E-04	6.60E-05	5.02E-05	2.16E-05
pdb1u53	193	1.40E-04	1.15E-04	6.43E-05	4.05E-05	2.18E-05

pdb2jay	193	1.49E-04	1.18E-04	6.69E-05	4.05E-05	2.48E-05
1MF7	194	1.53E-04	1.26E-04	6.95E-05	4.05E-05	2.80E-05
2VGA	194	1.55E-04	1.21E-04	7.04E-05	5.02E-05	2.48E-05
3BOR	194	1.53E-04	1.21E-04	7.04E-05	5.02E-05	1.87E-05
3O0P	194	1.60E-04	1.24E-04	7.21E-05	3.99E-05	2.78E-05
3VZH	194	1.53E-04	1.22E-04	6.95E-05	5.08E-05	2.18E-05
4ME2	194	1.45E-04	1.17E-04	6.69E-05	4.05E-05	2.48E-05
1YZF	195	1.57E-04	1.24E-04	7.21E-05	3.99E-05	2.48E-05
3SH4	195	1.51E-04	1.23E-04	6.86E-05	5.02E-05	1.55E-05
pdb1nkr	195	1.40E-04	1.18E-04	6.43E-05	3.99E-05	2.78E-05
2VY6	196	1.60E-04	1.25E-04	7.29E-05	3.99E-05	2.16E-05
pdb2osa	196	1.62E-04	1.25E-04	7.30E-05	3.02E-05	1.55E-05
pdb2p52	196	1.45E-04	1.21E-04	6.78E-05	3.99E-05	2.16E-05
4DB6	197	1.55E-04	1.27E-04	7.12E-05	5.02E-05	2.48E-05
pdb2pb7	197	1.81E-04	1.34E-04	8.17E-05	3.99E-05	2.48E-05
1CHD	198	1.45E-04	1.20E-04	8.34E-05	8.04E-05	2.16E-05
3KT2	198	1.40E-04	1.20E-04	6.52E-05	4.05E-05	2.52E-05
3P4L	198	1.60E-04	1.28E-04	7.12E-05	5.02E-05	2.48E-05
3W90	198	1.49E-04	1.24E-04	6.86E-05	5.02E-05	2.48E-05
pdb1kzf	198	1.42E-04	1.22E-04	6.69E-05	5.02E-05	2.16E-05
3L9U	199	1.55E-04	1.25E-04	7.13E-05	5.02E-05	2.48E-05
4M9K	199	1.60E-04	1.27E-04	7.21E-05	5.02E-05	2.48E-05
2FM9	201	1.42E-04	1.27E-04	6.52E-05	3.99E-05	2.48E-05
pdb1ijb	202	1.55E-04	1.31E-04	7.12E-05	4.05E-05	2.16E-05
pdb1v77	202	1.70E-04	1.35E-04	7.64E-05	5.02E-05	2.18E-05
4INK	203	1.53E-04	1.34E-04	6.95E-05	4.05E-05	2.48E-05
pdb2vfy	203	1.66E-04	1.35E-04	7.56E-05	5.02E-05	2.48E-05
4B2F	204	1.75E-04	1.38E-04	7.91E-05	5.08E-05	2.48E-05
pdb1dix	204	1.53E-04	1.87E-04	7.04E-05	4.05E-05	2.48E-05
2HLY	205	1.49E-04	1.28E-04	6.88E-05	4.05E-05	2.48E-05
pdb1xkr	205	1.51E-04	1.29E-04	6.95E-05	3.99E-05	2.48E-05
pdb1gsm	206	1.51E-04	1.34E-04	6.95E-05	5.02E-05	1.55E-05
pdb1mix	206	1.55E-04	1.35E-04	6.95E-05	5.02E-05	2.48E-05
pdb1uch	206	1.62E-04	1.34E-04	7.21E-05	3.02E-05	1.85E-05
pdb2eng	206	1.49E-04	1.28E-04	6.86E-05	5.02E-05	2.48E-05
pdb1otm	207	1.68E-04	1.35E-04	7.38E-05	4.05E-05	3.10E-05
pdb3cjw	207	1.68E-04	1.38E-04	7.74E-05	5.02E-05	2.48E-05
3NE0	208	1.51E-04	1.32E-04	6.95E-05	3.99E-05	2.48E-05
pdb3dlm	208	1.71E-04	1.41E-04	7.73E-05	5.02E-05	2.48E-05
pdb2erf	209	1.68E-04	1.38E-04	7.56E-05	5.08E-05	2.70E-05
2QHT	210	1.70E-04	1.43E-04	7.73E-05	5.02E-05	2.48E-05

3TUA	210	1.57E-04	1.35E-04	7.21E-05	3.99E-05	2.48E-05
pdb2z84	210	1.66E-04	1.39E-04	7.66E-05	5.08E-05	1.87E-05
1DZF	211	1.75E-04	1.48E-04	7.99E-05	8.04E-05	2.48E-05
4IBN	211	1.55E-04	1.39E-04	7.21E-05	3.99E-05	2.48E-05
pdb2ogq	211	1.68E-04	1.42E-04	7.56E-05	5.02E-05	2.48E-05
pdb1ojq	212	1.60E-04	1.40E-04	7.21E-05	3.02E-05	2.16E-05
pdb1zd8	212	1.77E-04	1.45E-04	7.99E-05	5.02E-05	2.48E-05
1IO2	213	1.60E-04	1.38E-04	7.99E-05	5.02E-05	2.48E-05
4JNF	213	1.73E-04	1.44E-04	7.83E-05	3.02E-05	2.80E-05
1RZ2	214	1.73E-04	1.47E-04	7.66E-05	3.99E-05	2.48E-05
3FTJ	214	1.64E-04	1.42E-04	7.38E-05	5.08E-05	2.48E-05
pdb2d4x	214	1.64E-04	1.39E-04	7.38E-05	5.02E-05	2.48E-05
1P3C	215	1.64E-04	1.46E-04	7.47E-05	5.02E-05	2.48E-05
1V8E	217	1.60E-04	1.44E-04	7.29E-05	3.99E-05	2.48E-05
pdb1gpp	217	1.57E-04	1.44E-04	7.21E-05	5.02E-05	2.48E-05
3ESU	218	1.75E-04	1.51E-04	7.91E-05	3.99E-05	2.48E-05
3VNE	218	1.60E-04	1.46E-04	7.47E-05	5.02E-05	2.48E-05
4FD6	218	1.75E-04	1.54E-04	7.82E-05	5.02E-05	2.48E-05
4JZC	218	1.73E-04	1.53E-04	7.82E-05	4.05E-05	2.48E-05
pdb1tk1	219	1.94E-04	1.61E-04	8.78E-05	5.02E-05	2.48E-05
pdb1mw7	220	1.62E-04	1.45E-04	7.38E-05	1.99E-05	2.78E-05
pdb1wnh	220	1.79E-04	1.53E-04	8.08E-05	5.02E-05	2.48E-05
1RC9	221	1.75E-04	1.53E-04	8.17E-05	5.02E-05	2.48E-05
2YMO	221	1.73E-04	1.54E-04	7.91E-05	5.08E-05	1.85E-05
3PKV	221	1.86E-04	1.59E-04	8.43E-05	4.05E-05	2.48E-05
2RFA	222	1.77E-04	1.57E-04	8.17E-05	3.99E-05	2.48E-05
3KR9	222	1.70E-04	1.56E-04	7.73E-05	3.02E-05	1.87E-05
3U0V	222	1.81E-04	1.57E-04	8.16E-05	5.02E-05	2.48E-05
4G54	222	1.83E-04	1.62E-04	8.25E-05	3.02E-05	2.48E-05
pdb1bol	222	1.62E-04	1.51E-04	7.47E-05	5.02E-05	2.48E-05
pdb1oa4	222	1.79E-04	1.61E-04	8.17E-05	1.99E-05	3.10E-05
pdb2a6z	222	1.70E-04	1.53E-04	7.73E-05	3.02E-05	2.48E-05
pdb2ahn	222	1.60E-04	1.48E-04	7.38E-05	4.05E-05	2.48E-05
1UOH	223	1.66E-04	1.52E-04	7.38E-05	4.05E-05	2.48E-05
2WNK	223	1.70E-04	1.56E-04	7.73E-05	5.02E-05	2.80E-05
pdb1uai	223	1.70E-04	1.54E-04	7.73E-05	5.02E-05	2.48E-05
pdb3ckf	223	1.55E-04	1.45E-04	7.03E-05	5.02E-05	2.48E-05
pdb1tje	224	1.79E-04	1.56E-04	7.73E-05	4.05E-05	2.80E-05
pdb2e3s	224	1.79E-04	1.56E-04	8.08E-05	4.05E-05	2.80E-05
4EUG	225	1.73E-04	1.59E-04	7.91E-05	5.02E-05	2.48E-05
2ZFY	226	1.84E-04	1.60E-04	8.00E-05	3.02E-05	2.48E-05

4JMP	226	1.83E-04	1.65E-04	8.34E-05	1.99E-05	2.48E-05
pdb1g8a	227	1.75E-04	1.63E-04	7.91E-05	5.02E-05	2.80E-05
pdb1k1b	228	1.77E-04	1.63E-04	7.99E-05	5.02E-05	2.48E-05
pdb2ahe	228	1.81E-04	1.63E-04	8.16E-05	3.02E-05	2.48E-05
4ERN	229	2.01E-04	1.72E-04	8.95E-05	5.02E-05	2.48E-05
pdb1yhh	229	1.86E-04	1.67E-04	8.51E-05	5.02E-05	2.48E-05
3PG4	230	1.68E-04	1.61E-04	7.83E-05	1.99E-05	2.18E-05
4B89	230	1.98E-04	1.73E-04	8.77E-05	5.08E-05	2.80E-05
pdb1p9q	230	1.68E-04	1.62E-04	7.82E-05	5.02E-05	2.78E-05
2PET	231	1.70E-04	1.68E-04	7.82E-05	1.99E-05	2.78E-05
pdb2eyi	234	1.77E-04	1.67E-04	8.08E-05	6.05E-05	1.87E-05
pdb2g5x	234	1.79E-04	1.69E-04	8.16E-05	5.02E-05	2.78E-05
1WR2	235	1.88E-04	1.75E-04	8.42E-05	5.98E-05	1.85E-05
2X8X	235	1.94E-04	1.77E-04	8.60E-05	5.02E-05	2.18E-05
pdb1z3y	236	1.92E-04	1.73E-04	8.60E-05	5.02E-05	2.78E-05
pdb3crm	236	1.77E-04	1.70E-04	8.17E-05	5.02E-05	2.48E-05
3DU1	237	1.92E-04	1.77E-04	8.60E-05	5.02E-05	2.48E-05
pdb2bjv	237	1.96E-04	1.78E-04	8.95E-05	5.02E-05	2.78E-05
3PHS	238	1.73E-04	1.70E-04	7.90E-05	5.02E-05	2.78E-05
pdb1xdz	238	2.03E-04	1.83E-04	9.47E-05	5.02E-05	2.48E-05
pdb2lao	238	1.81E-04	1.74E-04	8.17E-05	5.02E-05	2.48E-05
pdb3seb	238	1.94E-04	1.85E-04	8.95E-05	5.02E-05	2.16E-05
3TCQ	239	1.90E-04	1.79E-04	8.69E-05	3.02E-05	2.80E-05
3FTD	240	1.92E-04	1.81E-04	8.68E-05	5.02E-05	1.87E-05
pdb1iz4	241	1.88E-04	1.83E-04	8.51E-05	5.02E-05	2.80E-05
3KVD	242	2.01E-04	1.87E-04	9.03E-05	3.02E-05	2.48E-05
pdb1dua	242	1.88E-04	1.84E-04	8.51E-05	5.02E-05	2.48E-05
pdb2baa	243	1.83E-04	1.80E-04	8.43E-05	5.02E-05	2.80E-05
pdb2ad1	244	2.09E-04	1.91E-04	9.39E-05	5.02E-05	2.80E-05
4DIU	245	2.11E-04	1.97E-04	9.30E-05	5.02E-05	2.80E-05
4E40	245	1.88E-04	1.85E-04	8.43E-05	5.08E-05	2.48E-05
pdb1qts	247	1.90E-04	1.90E-04	8.60E-05	5.02E-05	2.78E-05
2NXC	249	1.86E-04	1.89E-04	8.43E-05	5.02E-05	2.80E-05
2OG4	249	2.01E-04	2.01E-04	9.03E-05	6.05E-05	2.78E-05
pdb1o0x	249	2.01E-04	2.00E-04	9.03E-05	5.02E-05	2.80E-05
pdb2ilr	249	1.96E-04	1.91E-04	8.77E-05	5.02E-05	2.78E-05
pdb2fbo	250	2.09E-04	2.00E-04	9.38E-05	5.02E-05	2.80E-05
3HC7	252	2.35E-04	2.15E-04	1.06E-04	2.06E-05	2.78E-05
pdb1vin	252	2.09E-04	2.02E-04	9.30E-05	6.05E-05	2.80E-05
pdb1xqo	253	2.09E-04	2.06E-04	9.46E-05	2.96E-05	2.16E-05
pdb2f1n	253	1.94E-04	1.96E-04	8.86E-05	5.02E-05	2.18E-05

3VN5	255	2.11E-04	2.08E-04	9.47E-05	5.02E-05	2.80E-05
pdb1rl0	255	1.94E-04	2.03E-04	8.77E-05	5.02E-05	2.80E-05
pdb1fqn	257	2.16E-04	2.13E-04	9.73E-05	5.02E-05	2.80E-05
3G40	258	2.14E-04	2.15E-04	9.56E-05	5.98E-05	2.80E-05
3I47	259	2.03E-04	2.13E-04	9.14E-05	5.02E-05	2.80E-05
1ES5	260	2.01E-04	2.16E-04	9.12E-05	8.04E-05	2.48E-05
3ZNY	260	2.16E-04	2.18E-04	9.73E-05	6.05E-05	2.80E-05
pdb1eg3	260	2.05E-04	2.10E-04	9.21E-05	5.08E-05	2.80E-05
pdb1es6	260	2.09E-04	2.13E-04	9.47E-05	5.02E-05	2.78E-05
pdb1arb	263	2.09E-04	2.21E-04	9.40E-05	5.02E-05	2.80E-05
4M67	264	2.33E-04	2.33E-04	1.04E-04	5.02E-05	2.80E-05
1OUV	265	2.14E-04	2.24E-04	9.30E-05	5.08E-05	2.16E-05
3ILS	265	2.01E-04	2.19E-04	9.39E-05	5.02E-05	2.80E-05
pdb3tgl	265	2.14E-04	2.32E-04	9.73E-05	3.02E-05	2.80E-05
2XMZ	266	2.24E-04	2.28E-04	1.01E-04	3.02E-05	4.03E-05
4FBR	266	1.98E-04	2.17E-04	9.03E-05	5.02E-05	2.80E-05
pdb2of3	266	2.18E-04	2.22E-04	9.73E-05	3.02E-05	2.78E-05
1AKO	268	2.72E-04	2.51E-04	1.17E-04	1.31E-04	5.89E-05
4IH1	268	2.18E-04	2.30E-04	9.82E-05	5.02E-05	2.80E-05
pdb1uek	268	2.18E-04	2.28E-04	9.82E-05	5.02E-05	2.80E-05
4MQM	269	2.46E-04	2.44E-04	1.16E-04	6.05E-05	2.78E-05
2XJ4	270	2.18E-04	2.34E-04	9.75E-05	5.02E-05	2.78E-05
3M66	270	2.29E-04	2.33E-04	1.02E-04	6.05E-05	2.78E-05
pdb1oi7	270	2.01E-04	2.30E-04	9.21E-05	5.02E-05	2.80E-05
pdb2h7o	270	2.20E-04	2.31E-04	9.73E-05	4.05E-05	2.78E-05
2EGU	271	1.96E-04	2.27E-04	8.77E-05	5.02E-05	2.16E-05
3PWZ	271	2.27E-04	2.34E-04	1.01E-04	5.02E-05	3.10E-05
2VFB	272	2.26E-04	2.43E-04	1.02E-04	5.02E-05	3.10E-05
3H04	272	2.35E-04	2.43E-04	1.08E-04	5.02E-05	3.12E-05
pdb2qol	273	2.29E-04	2.41E-04	1.03E-04	6.05E-05	2.80E-05
3SIH	274	2.27E-04	2.40E-04	1.02E-04	5.98E-05	2.80E-05
3W9U	274	2.42E-04	2.52E-04	1.08E-04	5.02E-05	3.41E-05
pdb1gqz	274	2.16E-04	2.38E-04	9.73E-05	5.98E-05	2.78E-05
pdb2ci3	274	2.22E-04	2.38E-04	1.01E-04	6.05E-05	2.78E-05
pdb2plc	274	2.33E-04	2.43E-04	1.03E-04	3.02E-05	2.78E-05
2UYO	275	2.14E-04	2.38E-04	9.56E-05	5.02E-05	2.18E-05
3ZXY	275	2.07E-04	2.34E-04	9.47E-05	3.02E-05	2.80E-05
3BZG	278	2.39E-04	2.51E-04	1.07E-04	3.02E-05	2.80E-05
pdb1r3f	278	2.27E-04	2.46E-04	1.01E-04	6.05E-05	3.10E-05
3F7M	279	2.14E-04	2.45E-04	9.90E-05	5.02E-05	3.12E-05
3TEF	279	2.29E-04	2.54E-04	1.03E-04	3.02E-05	2.16E-05

pdb3eg4	279	2.39E-04	2.55E-04	1.08E-04	6.05E-05	2.18E-05
pdb1jks	280	2.48E-04	2.62E-04	1.09E-04	3.02E-05	3.10E-05
4HJP	281	2.42E-04	2.59E-04	1.09E-04	3.02E-05	3.12E-05
pdb2boe	281	2.27E-04	2.53E-04	1.03E-04	3.02E-05	2.80E-05
1SR8	282	2.50E-04	2.64E-04	1.09E-04	3.02E-05	2.80E-05
3RGI	282	2.50E-04	2.68E-04	1.13E-04	6.05E-05	3.12E-05
pdb1f00	282	2.11E-04	2.49E-04	9.55E-05	6.05E-05	3.10E-05
2HE7	283	2.61E-04	2.73E-04	1.15E-04	4.05E-05	3.10E-05
2RIK	283	2.11E-04	2.49E-04	9.49E-05	5.02E-05	3.10E-05
pdb1cnv	283	2.48E-04	2.65E-04	1.11E-04	5.08E-05	2.80E-05
2BV9	284	2.39E-04	2.69E-04	1.07E-04	3.02E-05	3.10E-05
2CWC	284	2.42E-04	2.72E-04	1.09E-04	5.02E-05	3.10E-05
pdb1ltu	284	2.37E-04	2.62E-04	1.08E-04	5.98E-05	2.78E-05
pdb2zco	284	2.63E-04	2.70E-04	1.15E-04	6.05E-05	3.10E-05
2OPW	286	2.27E-04	2.87E-04	1.02E-04	6.05E-05	2.80E-05
pdb1u6d	288	2.29E-04	2.62E-04	1.03E-04	5.02E-05	3.10E-05
4M9P	289	2.33E-04	2.68E-04	1.05E-04	6.05E-05	3.10E-05
pdb1nar	289	2.50E-04	2.72E-04	1.11E-04	5.98E-05	3.10E-05
pdb1mtz	290	2.39E-04	2.72E-04	1.07E-04	6.05E-05	3.10E-05
pdb1bn6	291	2.37E-04	2.78E-04	1.08E-04	6.05E-05	3.10E-05
pdb1h6t	291	2.26E-04	2.84E-04	1.02E-04	5.98E-05	3.10E-05
pdb1ks9	291	2.29E-04	2.72E-04	1.03E-04	5.02E-05	3.10E-05
pdb1yrw	291	2.63E-04	2.81E-04	1.18E-04	5.98E-05	2.80E-05
1U2K	292	2.61E-04	3.00E-04	1.16E-04	5.98E-05	3.41E-05
pdb1gcu	292	2.48E-04	2.74E-04	1.09E-04	6.05E-05	2.16E-05
pdb1lkf	292	2.42E-04	2.75E-04	1.08E-04	6.05E-05	2.78E-05
1SUU	293	2.61E-04	2.82E-04	1.12E-04	5.98E-05	3.12E-05
3G9T	294	2.52E-04	2.82E-04	1.14E-04	3.99E-05	2.18E-05
3SV0	294	2.50E-04	2.82E-04	1.12E-04	6.05E-05	3.10E-05
4D8L	294	2.50E-04	2.82E-04	1.14E-04	6.05E-05	3.10E-05
4EVF	294	2.65E-04	2.89E-04	1.16E-04	6.05E-05	3.10E-05
pdb3c5v	294	2.46E-04	2.80E-04	1.10E-04	7.01E-05	3.10E-05
1LCY	296	2.29E-04	2.80E-04	1.03E-04	5.08E-05	3.41E-05
3K8W	296	2.33E-04	2.73E-04	1.05E-04	5.98E-05	2.78E-05
pdb2ptd	296	2.70E-04	2.94E-04	1.19E-04	5.98E-05	3.10E-05
3JSN	297	2.52E-04	2.91E-04	1.12E-04	5.98E-05	3.12E-05
pdb1rwr	297	2.55E-04	2.91E-04	1.15E-04	5.02E-05	3.10E-05
2X4L	298	2.31E-04	2.84E-04	1.03E-04	2.96E-05	2.78E-05
2Z1E	298	2.59E-04	2.92E-04	1.14E-04	3.99E-05	5.58E-05
pdb1fhu	298	2.35E-04	2.82E-04	1.06E-04	5.98E-05	3.73E-05
2IM9	299	2.74E-04	3.01E-04	1.18E-04	5.98E-05	2.48E-05

4ETX	300	2.50E-04	2.92E-04	1.11E-04	3.02E-05	3.10E-05
pdb1a3h	300	2.50E-04	2.93E-04	1.11E-04	6.05E-05	2.48E-05
pdb2f68	300	2.59E-04	2.95E-04	1.16E-04	6.05E-05	3.10E-05
pdb2ixm	300	2.59E-04	2.97E-04	1.15E-04	3.02E-05	3.10E-05
pdb2qy9	300	2.44E-04	2.89E-04	1.12E-04	6.05E-05	3.41E-05
4G3N	302	2.50E-04	2.96E-04	1.12E-04	6.05E-05	3.41E-05
pdb1bqc	302	3.15E-04	3.24E-04	1.37E-04	5.98E-05	3.12E-05
pdb2h14	303	2.50E-04	2.97E-04	1.11E-04	3.02E-05	3.17E-05
1NTY	305	2.55E-04	3.04E-04	1.10E-04	8.04E-05	3.41E-05
pdb2h2z	306	2.46E-04	3.01E-04	1.09E-04	5.59E-05	3.10E-05
pdb1arl	307	2.57E-04	3.16E-04	1.15E-04	7.01E-05	3.10E-05
2X0C	308	2.67E-04	3.12E-04	1.06E-04	6.05E-05	3.10E-05
2XBG	308	2.74E-04	3.22E-04	1.22E-04	5.98E-05	3.10E-05
pdb1ak1	308	2.55E-04	3.11E-04	1.14E-04	6.05E-05	3.10E-05
pdb2iy9	309	2.74E-04	3.19E-04	1.22E-04	6.05E-05	3.41E-05
pdb2p4h	310	2.44E-04	3.30E-04	1.10E-04	6.05E-05	3.10E-05
2YLH	311	2.46E-04	3.16E-04	1.10E-04	7.01E-05	3.12E-05
pdb1pgs	311	2.55E-04	3.31E-04	1.15E-04	6.05E-05	4.35E-05
3K6U	312	2.65E-04	3.22E-04	1.21E-04	7.01E-05	3.10E-05
3UAH	312	2.87E-04	3.31E-04	1.26E-04	6.05E-05	3.10E-05
pdb1qwk	312	2.72E-04	3.22E-04	1.20E-04	6.05E-05	3.10E-05
pdb2cyg	312	2.46E-04	3.14E-04	1.12E-04	6.05E-05	3.41E-05
4IQM	313	2.85E-04	3.32E-04	1.25E-04	7.07E-05	3.41E-05
1TM2	314	2.55E-04	3.23E-04	1.12E-04	3.02E-05	2.16E-05
3BB7	314	2.55E-04	3.25E-04	1.14E-04	6.37E-05	3.10E-05
3LPZ	314	2.55E-04	3.38E-04	1.15E-04	6.05E-05	3.39E-05
3QH4	314	2.48E-04	3.21E-04	1.12E-04	5.98E-05	4.35E-05
4E9L	314	2.44E-04	3.18E-04	1.10E-04	3.99E-05	3.10E-05
pdb1fcq	314	2.85E-04	3.33E-04	1.26E-04	6.05E-05	3.49E-05
pdb1y9u	314	2.65E-04	3.22E-04	1.17E-04	6.05E-05	3.41E-05
3ETV	315	2.74E-04	3.28E-04	1.20E-04	7.07E-05	3.10E-05
pdb2oy7	315	2.42E-04	3.12E-04	1.06E-04	3.99E-05	3.12E-05
3EVN	316	2.70E-04	3.44E-04	1.21E-04	7.01E-05	2.16E-05
4J87	316	2.93E-04	3.42E-04	1.30E-04	8.04E-05	2.48E-05
pdb3civ	316	2.87E-04	3.34E-04	1.25E-04	6.05E-05	3.10E-05
1NIJ	317	2.78E-04	3.42E-04	1.22E-04	7.07E-05	3.41E-05
3CML	317	2.98E-04	3.45E-04	1.29E-04	7.01E-05	3.41E-05
3PST	317	3.00E-04	3.45E-04	1.34E-04	6.05E-05	3.41E-05
4AFV	317	2.55E-04	3.27E-04	1.16E-04	6.05E-05	3.41E-05
4K5Q	317	2.72E-04	3.36E-04	1.22E-04	3.99E-05	3.41E-05
pdb1lzl	317	2.78E-04	3.41E-04	1.23E-04	6.05E-05	3.41E-05

4LSW	318	2.57E-04	3.30E-04	1.15E-04	6.05E-05	3.12E-05
pdb1w7b	319	2.65E-04	3.41E-04	1.17E-04	6.05E-05	3.41E-05
3PT5	320	3.15E-04	3.60E-04	1.41E-04	6.05E-05	3.12E-05
pdb2rjd	321	2.76E-04	3.42E-04	1.22E-04	6.05E-05	3.41E-05
1WLY	322	2.57E-04	3.47E-04	1.14E-04	4.05E-05	3.73E-05
3R2G	323	2.89E-04	3.57E-04	1.26E-04	7.07E-05	4.03E-05
4FH3	323	2.80E-04	3.49E-04	1.25E-04	6.05E-05	3.41E-05
pdb1gxn	323	2.67E-04	3.46E-04	1.20E-04	5.98E-05	3.41E-05
pdb3app	323	2.55E-04	3.40E-04	1.14E-04	4.05E-05	2.80E-05
pdb1wer	324	2.80E-04	3.58E-04	1.25E-04	7.07E-05	3.10E-05
pdb1xfk	324	2.93E-04	3.58E-04	1.31E-04	3.99E-05	3.41E-05
3IM1	325	2.65E-04	3.47E-04	1.22E-04	7.07E-05	3.10E-05
3N11	325	2.85E-04	3.58E-04	1.26E-04	5.98E-05	3.39E-05
3N2T	327	2.85E-04	3.80E-04	1.26E-04	7.07E-05	3.12E-05
4J0W	327	2.87E-04	3.61E-04	1.28E-04	6.05E-05	3.10E-05
3GMS	331	3.04E-04	3.77E-04	1.33E-04	6.05E-05	3.41E-05
pdb1fo9	331	2.83E-04	3.71E-04	1.27E-04	5.98E-05	3.41E-05
pdb2z0m	331	2.87E-04	3.67E-04	1.27E-04	5.02E-05	4.03E-05
pdb1ceo	332	3.30E-04	3.94E-04	1.45E-04	5.98E-05	2.48E-05
pdb1ri6	333	2.98E-04	3.84E-04	1.31E-04	7.01E-05	3.12E-05
pdb2cy7	333	3.26E-04	3.91E-04	1.44E-04	6.05E-05	3.10E-05
3VJ8	335	3.00E-04	3.84E-04	1.32E-04	7.07E-05	3.41E-05
pdb1xix	335	2.83E-04	3.81E-04	1.25E-04	7.01E-05	3.41E-05
pdb1i9y	336	2.98E-04	3.89E-04	1.33E-04	7.01E-05	3.41E-05
pdb2bjq	340	2.89E-04	3.93E-04	1.30E-04	4.05E-05	3.41E-05
2OKT	342	2.87E-04	3.95E-04	1.27E-04	7.07E-05	3.41E-05
3HR8	342	2.80E-04	3.93E-04	1.25E-04	5.98E-05	3.41E-05
pdb1nj4	343	2.93E-04	4.01E-04	1.30E-04	7.01E-05	3.39E-05
1Z15	344	2.72E-04	3.97E-04	1.21E-04	7.01E-05	3.41E-05
3I2N	345	3.30E-04	4.22E-04	1.47E-04	8.04E-05	3.39E-05
3UGU	345	3.39E-04	4.26E-04	1.50E-04	7.01E-05	3.39E-05
4AM1	345	3.17E-04	4.15E-04	1.39E-04	6.05E-05	3.41E-05
3PTE	347	2.98E-04	4.12E-04	1.31E-04	7.01E-05	2.80E-05
4AD1	349	3.62E-04	4.42E-04	1.56E-04	7.07E-05	3.41E-05
3Q1C	351	3.04E-04	4.26E-04	1.33E-04	7.14E-05	4.03E-05
1SNT	352	3.26E-04	4.31E-04	1.38E-04	7.07E-05	2.48E-05
3AAP	353	3.21E-04	4.47E-04	1.42E-04	7.01E-05	3.73E-05
3IU0	354	3.34E-04	4.45E-04	1.48E-04	7.01E-05	3.41E-05
4HDJ	355	3.34E-04	4.52E-04	1.47E-04	7.07E-05	3.41E-05
pdb2pge	356	3.17E-04	4.43E-04	1.38E-04	6.05E-05	3.41E-05
3PZ9	357	3.19E-04	4.54E-04	1.41E-04	7.01E-05	3.71E-05

3IVF	358	3.08E-04	4.46E-04	1.36E-04	7.01E-05	4.35E-05
pdb1qcx	359	3.15E-04	4.54E-04	1.39E-04	6.05E-05	3.73E-05
pdb1eur	361	3.02E-04	4.52E-04	1.39E-04	5.98E-05	3.41E-05
pdb1wos	361	3.30E-04	4.61E-04	1.44E-04	7.01E-05	3.73E-05
1GCE	362	3.00E-04	4.58E-04	1.37E-04	9.07E-05	4.03E-05
3GD0	362	3.13E-04	4.76E-04	1.40E-04	7.01E-05	2.78E-05
pdb1cem	363	3.32E-04	4.66E-04	1.44E-04	7.01E-05	3.41E-05
pdb1k30	363	3.26E-04	4.65E-04	1.46E-04	7.01E-05	3.41E-05
pdb1nc5	363	3.19E-04	4.62E-04	1.40E-04	7.07E-05	3.73E-05
2ZQ5	365	3.11E-04	4.67E-04	1.37E-04	7.01E-05	3.41E-05
2PEF	366	3.21E-04	4.81E-04	1.40E-04	7.07E-05	3.73E-05
3KCI	366	3.60E-04	4.92E-04	1.58E-04	5.02E-05	2.78E-05
3V55	368	3.32E-04	4.83E-04	1.46E-04	7.01E-05	3.71E-05
3PZF	369	3.21E-04	4.80E-04	1.42E-04	7.01E-05	2.80E-05
3KJT	370	3.11E-04	4.81E-04	1.38E-04	7.01E-05	3.41E-05
3NE4	370	3.34E-04	4.89E-04	1.47E-04	7.01E-05	2.80E-05
pdb2zhv	370	3.36E-04	4.87E-04	1.49E-04	7.01E-05	3.41E-05
4IC4	372	3.28E-04	4.91E-04	1.44E-04	7.07E-05	4.03E-05
pdb1c3p	372	3.84E-04	5.16E-04	1.68E-04	7.07E-05	3.41E-05
2FEZ	373	3.41E-04	4.97E-04	1.49E-04	7.01E-05	4.03E-05
2HY7	373	3.34E-04	5.04E-04	1.48E-04	7.01E-05	2.78E-05
pdb1vf8	373	3.41E-04	5.01E-04	1.49E-04	7.07E-05	3.73E-05
2Q43	375	3.34E-04	5.12E-04	1.46E-04	8.04E-05	3.71E-05
3ANJ	376	3.47E-04	5.23E-04	1.52E-04	5.02E-05	3.71E-05
3M7D	376	3.32E-04	5.08E-04	1.47E-04	5.02E-05	3.41E-05
4IZO	376	3.24E-04	5.02E-04	1.43E-04	7.07E-05	3.71E-05
pdb1bhe	376	3.13E-04	5.02E-04	1.37E-04	7.01E-05	3.71E-05
pdb2b78	376	3.30E-04	5.00E-04	1.44E-04	5.02E-05	4.05E-05
pdb1e4f	378	3.43E-04	5.13E-04	1.49E-04	7.01E-05	3.73E-05
1EDG	380	3.37E-04	5.21E-04	1.49E-04	1.01E-04	4.35E-05
pdb2sil	381	3.34E-04	5.22E-04	1.46E-04	7.01E-05	2.78E-05
2G5D	382	3.35E-04	5.22E-04	1.43E-04	8.04E-05	3.73E-05
pdb1wyc	384	3.28E-04	5.26E-04	1.44E-04	7.01E-05	3.71E-05
pdb1fc9	386	3.28E-04	5.36E-04	1.43E-04	5.02E-05	3.73E-05
3H2G	387	3.54E-04	5.50E-04	1.59E-04	7.01E-05	3.73E-05
3G6L	388	3.49E-04	5.49E-04	1.55E-04	8.04E-05	3.73E-05
pdb2ie8	390	3.26E-04	5.42E-04	1.45E-04	7.01E-05	3.71E-05
2WN4	391	3.86E-04	5.71E-04	1.68E-04	8.04E-05	3.73E-05
pdb2pbo	394	3.62E-04	5.71E-04	1.58E-04	5.02E-05	4.03E-05
pdb1io1	395	3.11E-04	5.54E-04	1.36E-04	8.04E-05	3.71E-05
3GRH	397	3.56E-04	5.68E-04	1.57E-04	7.07E-05	3.73E-05

2I49	398	3.52E-04	5.93E-04	1.55E-04	7.01E-05	2.92E-05
2GGO	401	3.45E-04	6.19E-04	1.53E-04	7.07E-05	3.71E-05
3OGG	404	3.77E-04	6.14E-04	1.65E-04	8.04E-05	3.71E-05
pdb1h13	404	4.04E-04	6.23E-04	1.75E-04	8.04E-05	3.73E-05
3GRE	408	3.54E-04	6.17E-04	1.58E-04	7.07E-05	4.03E-05
3DMS	413	3.77E-04	6.46E-04	1.64E-04	8.04E-05	4.05E-05
pdb3bok	416	3.99E-04	6.61E-04	1.73E-04	8.04E-05	4.05E-05
3ACP	417	3.76E-04	6.70E-04	1.64E-04	5.08E-05	4.33E-05
pdb1gso	419	3.80E-04	6.60E-04	1.67E-04	8.04E-05	4.03E-05
pdb3cj1	419	3.67E-04	6.69E-04	1.62E-04	8.04E-05	4.03E-05
pdb1sqg	424	3.99E-04	6.79E-04	1.73E-04	9.07E-05	4.03E-05
pdb1yks	431	3.77E-04	7.13E-04	1.66E-04	8.04E-05	4.03E-05
pdb1l2l	432	3.95E-04	7.20E-04	1.71E-04	9.00E-05	4.03E-05
pdb1tuo	437	3.84E-04	7.46E-04	1.71E-04	9.00E-05	4.33E-05
3OF7	438	4.53E-04	7.69E-04	1.97E-04	9.00E-05	4.03E-05
3CB6	441	4.70E-04	7.97E-04	2.02E-04	9.07E-05	4.03E-05
3P1W	442	4.36E-04	7.81E-04	1.89E-04	8.04E-05	4.96E-05
4IDH	451	4.16E-04	8.09E-04	1.82E-04	8.04E-05	4.35E-05
pdb2ece	455	4.34E-04	8.31E-04	1.89E-04	9.07E-05	4.35E-05
pdb2r60	456	4.70E-04	8.51E-04	2.02E-04	9.00E-05	4.35E-05
pdb3csg	458	4.25E-04	8.43E-04	1.85E-04	9.07E-05	4.33E-05
1ZCJ	459	4.55E-04	8.69E-04	1.95E-04	1.00E-04	4.35E-05
pdb1vjs	469	4.83E-04	9.15E-04	2.07E-04	9.00E-05	3.73E-05
4J0U	471	5.03E-04	9.34E-04	2.16E-04	9.07E-05	4.33E-05
1U09	476	4.51E-04	9.34E-04	1.89E-04	1.11E-04	4.35E-05
3VSR	493	4.64E-04	1.04E-03	2.04E-04	1.11E-04	4.96E-05
pdb1cwy	500	4.87E-04	1.07E-03	2.12E-04	1.00E-04	4.64E-05

References

1. Kamide, K. and Dobashi, T. (2000), *Physical Chemistry of Polymer Solutions. Theoretical Background*. Elsevier Science.
2. Ruiz-Blanco, Y.B., García, Y., Sotomayor-Torres, C.M. and Marrero-Ponce, Y. (2010) New Set of 2D/3D Thermodynamic Indices for Proteins. A Formalism Based on “Molten Globule” Theory. *Physics Procedia*, **8**, 63-72.
3. Ruiz-Blanco, Y.B., Marrero-Ponce, Y., Paz, W., García, Y. and Salgado, J. (2013) Global Stability of Protein Folding from an Empirical Free Energy Function. *Journal of Theoretical Biology,* **321**, 44-53.
4. Jiang, L., Kuhlman, B., Kortemme, T. and Baker, D. (2005) A “solvated rotamer” approach to modeling water-mediated hydrogen bonds at protein–protein interfaces. *PROTEINS: Structure, Function, and Bioinformatics*, **58**, 893–904.
5. Kyte, J. and Doolitle, R.F. (1982) A Simple Method for Displaying the Hydropathic Character of a Protein. . *J. Mol. Biol.*, **157**, 105-132.
6. Collantes, E.R. and Dunn-III, W.J. (1995) Amino acid side chain descriptors for quantitative structure-activity relationship studies of peptide analogues. *J. Med. Chem.*, **38**, 2705-2713.
7. Plaxco, K.W., Simons, K.T. and Baker, D. (1998) Contact Order, Transition State Placement and the Refolding Rates of Single Domain Proteins. *J. Mol. Biol.*, **277**, 985-994.
8. Burrage, J.S.a.K. (2006) Predicting residue-wise contact orders in proteins by support vector regression. *BMC Bioinformatics*, **425**.
9. Nolting, B., Schalike, W., Hampel, P., Grundig, F., Gantert, S., Sips, N., Bandlow, W. and Qi, P.X. (2003) Structural determinants of the rate of protein folding. *J. Theor. Biol.* , **223**, 299–307.
10. Micheletti, C. (2003) Prediction of Folding Rates and Transition-State Placement From Native-State Geometry. *PROTEINS: Structure, Function, and Genetics*, **51**, 74–84.
11. Zhou, H. and Zhou, Y. (2002) Folding Rate Prediction Using Total Contact Distance. *Biophysical Journal*, **82**, 458–463.
12. Lehninger. (2005), *Biochemistry,* pp. 76-115.
13. Zamyatin, A.A. (1972) Protein Volume in Solution. *Prog. Biophys. Mol. Biol.* , **24**, 107-123.
14. Hellberg S., S., M., Skagerberg B., Wold, S. (1987) Peptide Quantitative Structure-Activity Relationship, a Multivariate Approach. *J. Med. Chem,* **30**, 1126-1135. .
15. Levitt, M. (1978) Conformational Preferences of Amino Acids in Globular Proteins. *Biochemistry*, **17**.
