# Supplementary material for: ProtDCal: A program to compute general-purpose-numerical descriptors for sequences and 3D-structures of proteins
Source: BMC Bioinformatics. 2015 May 16;16:162. doi: 10.1186/s12859-015-0586-0 (PMC4432771; doi:10.1186/s12859-015-0586-0)
Supplement: Additional file 9: — Tutorial guide of how to use ProtDCal program as well as a practical example of how to train a model using ProtDCal's features and Weka. [file 12859_2015_586_MOESM9_ESM.pdf]

ProtDCal

A Program for **Prot**ein **D**escriptors **Cal**culation

USER MANUAL

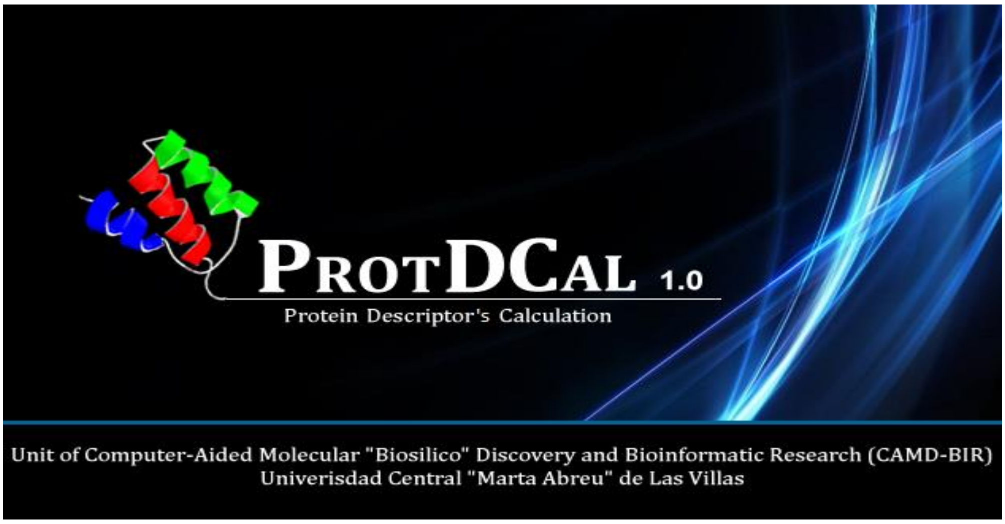

CONTENT TABLE

ABOUT US **3**
GETTING STARTED **3**
WORKSPACE **5**
BASIC ENVIRONMENT **6**
Indices Panel 7
Groups Panel 10
Aggregation Operators Panel 10
DESCRIPTION OF MENUS **12**
OUTPUT FILES **17**
PARAMETERS CONFIGURATION **18**
Organizing the output file 19
PROJECTS **19**
Project Structure 19
Loading a project 25
USER-SPECIFIED INDICES **25**
USER-SPECIFIED GROUPS **26**
EXECUTING CALCULATIONS **27**
BASIC MODELING WORKFLOW USING PROTDCAL AND WEKA **28**
Exemplification in the prediction of N-glycosylation 28

ABOUT US

ProtDCal is a protein-modeling platform developed and maintained in the Unit of Computer-Aided Molecular Discovery and Bioinformatics Research (CAMD-BIR) of the Universidad Central "Marta Abreu" de Las Villas (UCLV) and the Department of Systems & Computer Engineering of Carleton University (CU).

Project members:
Yasser B. Ruiz-Blanco (yasserrb@uclv.edu.cu) (UCLV)
Waldo Paz Rodriguez (waldopaz@uclv.cu) (UCLV)
Yovani Marrero-ponce, Ph.D. (ymarrero77@yahoo.es) (UCLV)
James Green, Ph.D. (jrgreen@sce.carleton.ca) (CU)

Citations:
Ruiz-Blanco, Y. B, et al., *ProtDCal: A Program to Compute General-Purpose-Numerical Descriptors for Sequences and 3D-Structures of Proteins*. BMC Bioinformatics, 2015. Submitted.
Ruiz-Blanco, Y.B., et al., *A Hooke's law-based approach to protein folding rate.* Journal of Theoretical Biology, 2015. **364**: p. 407-417.
Ruiz-Blanco, Y.B., et al., *A physics-based scoring function for protein structural decoys: Dynamic testing on targets of CASP-ROLL.* Chemical Physics Letters, 2014. **610–611**: p. 135-140.
Ruiz-Blanco, Y.B., et al., *Global Stability of Protein Folding from an Empirical Free Energy Function.* Journal of Theoretical Biology, 2013. **321**: p. 44-53.

GETTING STARTED

ProtDCal is a user-friendly software package that was developed to generate a variety of numeric descriptors for protein structures and sequences. This manual is intended to provide an overview of the main interfaces and functionalities of the program. As part of the current distribution of ProtDCal, one can find a similar tutorial and a theory section describing the formalism and parameters of the indices implemented in the program.

ProtDCal's feature generation strategy comprises four hierarchical levels:

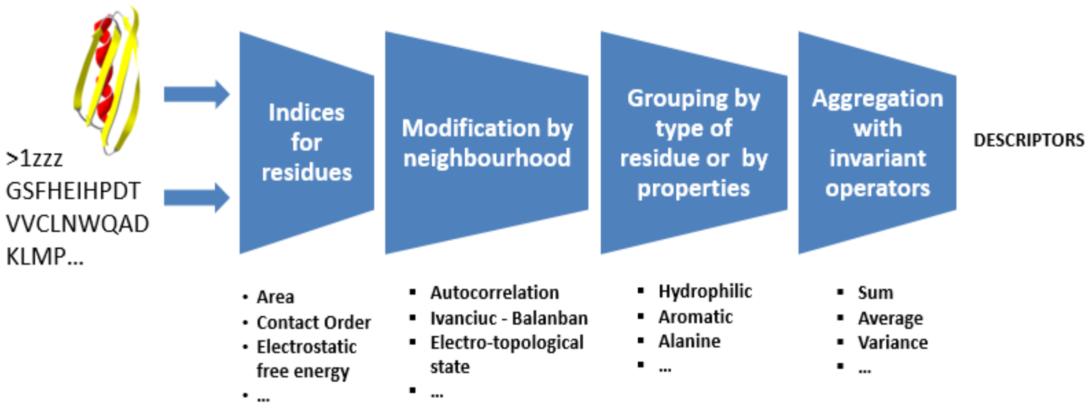

i) An initial layer intended to select the type of indices to encode for each residue. These indices are grouped in three main classes:

Thermodynamics, which are almost all novel indices designed in our laboratory based on an empirical model of the main factors involved in the stability of protein structures. These indices are, in turn, divided into two panels grouping, on one side, those that are defined for 3D folded structures and on the other side, those based on information relating to the protein sequence. These indices refer to the contribution of the folded and unfolded (reference) states of a protein chain.

Topographic, which include many of the contact-based descriptors with proven correlation with the protein folding rate constant, e. g. the relative contact order (CO), the total contact distance (TCD), the cliquishness (CLQ), etc. These indices were defined originally as global metrics, however, they were modified to obtain a value for each residue of a protein. Each contact of the protein is weighted by a determined residue property selected in this interface. The weighting procedure is conducted by multiplying the values of the selected property for both residues that are in contact.

Property-based indices, this final group encloses a number of chemical-physical and structural properties of each type of residue such as hydrophobicity, electronic charge index, molar weight, volume, isotropic surface area, etc.

ii) *Modification operators,* these approaches are intended to modify the value of a selected index for a given residue according to the residues within a vicinity defined

by the type of modification operator and its parameter value (e.g. for the autocorrelation operator with parameter k = 2, the neighbourhood of residue *i* comprises the residues in positions $i \pm 2$). . ProtDCal implements five modification operators that can be selected in the Menu: '*Options/Weighting operators*'.

iii) A third layer named 'Groups' is intended to select one or more groups of residues according their ID or type. When a group of residues is selected, an array of index values is obtained corresponding to the residues in the group. In addition to the implemented grouping approaches, an option is included by which users can define their own groups of residues (see the option *Groups* in menu *Option*).

iv) A fourth layer comprises several aggregation operators that are used to combine an array of values (from a group of residues) into a single value (descriptor) reflecting the distribution of the index within that group. Some examples of these aggregation operators are the sum, average, variance, kurtosis, geometric mean, information content, etc.

The output of the calculation shows the full combination of indices, groups and aggregation operators selected in each panel. The input file formats of the software can be either PDB or FASTA; for PDB files, all indices can be computed, whereas for FASTA files, only the indices of the second (Thermodynamics indices for sequences) and fourth (Properties-based indices) panels can be evaluated. Multiple proteins may be input simultaneously. The output files of ProtDCal calculations are two tab-delimited text documents named <name>_AA.txt and <name>_Prot.txt which store all the descriptors for each residue of each protein and the descriptors for the combinations of indices, groups, and aggregation operators for each protein respectively.

WORKSPACE

The ProtDCal workspace consists of the program folders:

- Datasets: Containing all the input data files in PDB or FASTA format.
- Outputs: Containing the output files of the program (<name>_AA, <name>_Prot, etc.).

- Projects: Containing all project files (<name>.proj).
- Help: Containing all the documentation files about the program and descriptors.

BASIC ENVIRONMENT

When the application is executed, the following launch screen is displayed:

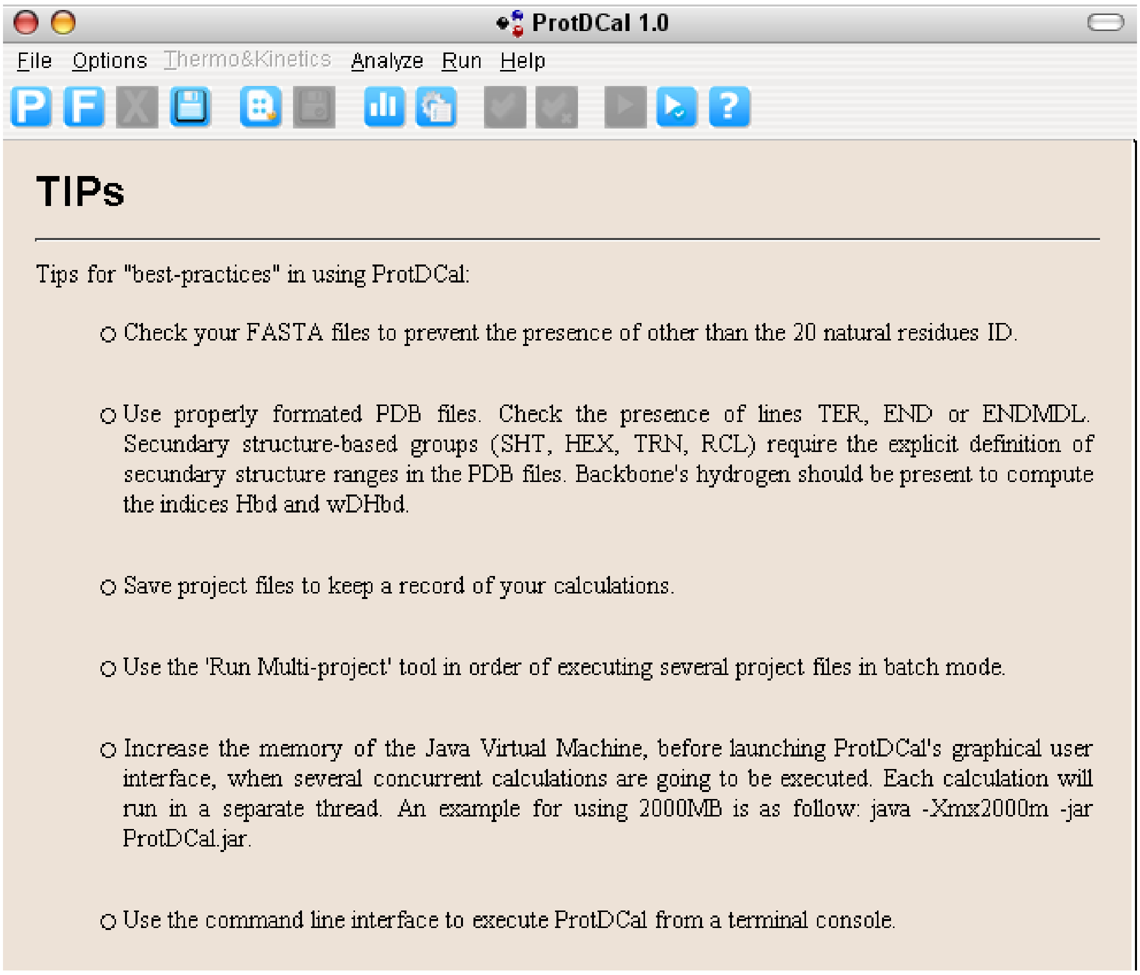

Once a dataset is uploaded, the interface provides access to the available indices depending on the input file type (PDB or FASTA format). The next figure depicts the interface with access to all type of indices, as is obtained when PDB files are used:

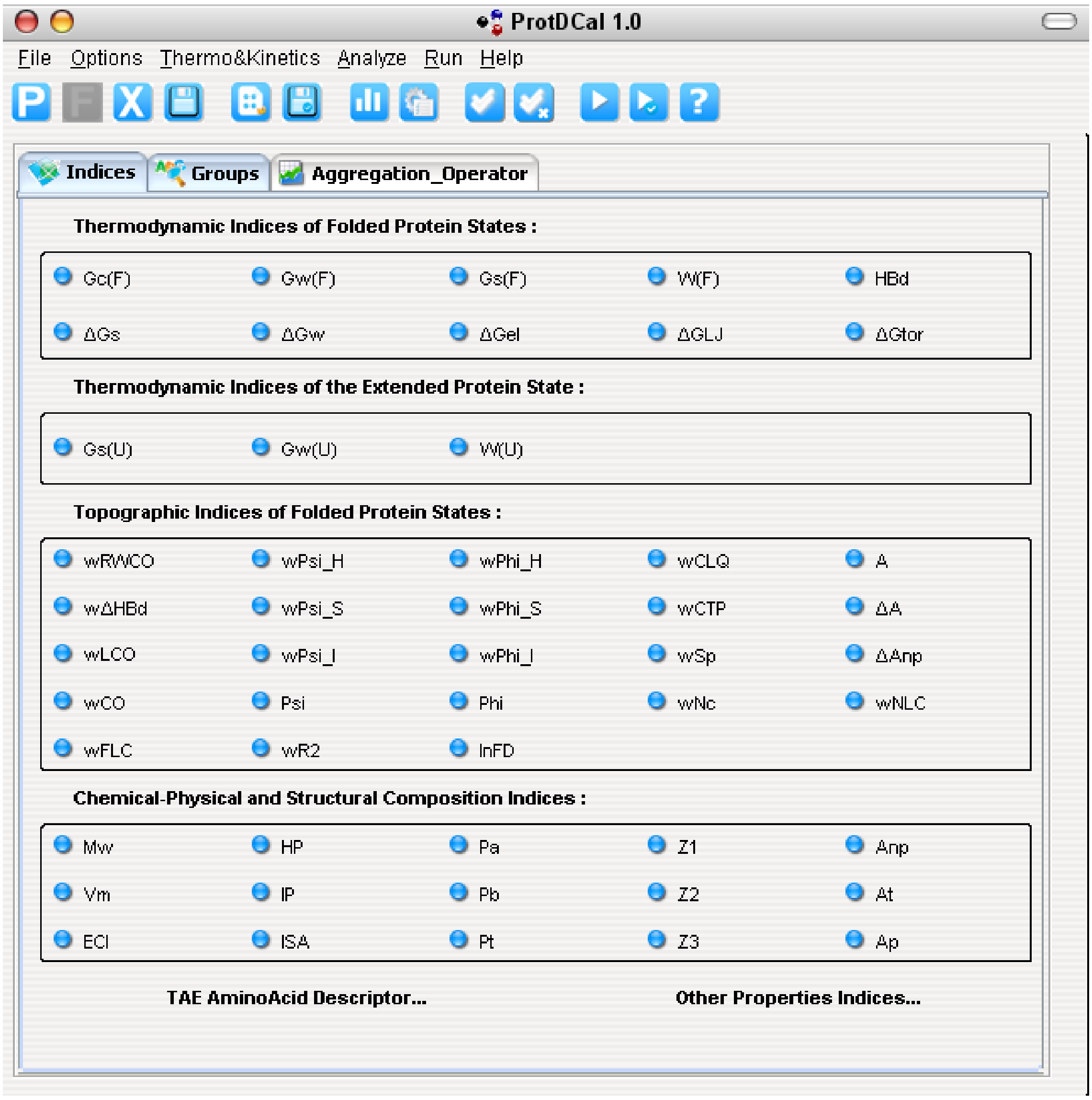

The panels below the toolbar represent the three of the hierarchical levels described above (*Modification Operators* are accessed via the *Options* menu). When you mouse over each element, a brief text description pops up explaining its functionality. Panels (indices, groups and aggregation operators) are subdivided, according to their nature, into several subpanels.

Indices Panel

The panel of indices is divided into four subpanels (Thermodynamic Indices for Structures, Thermodynamic Indices for Sequences, Topographic Indices, and Properties-based Indices).

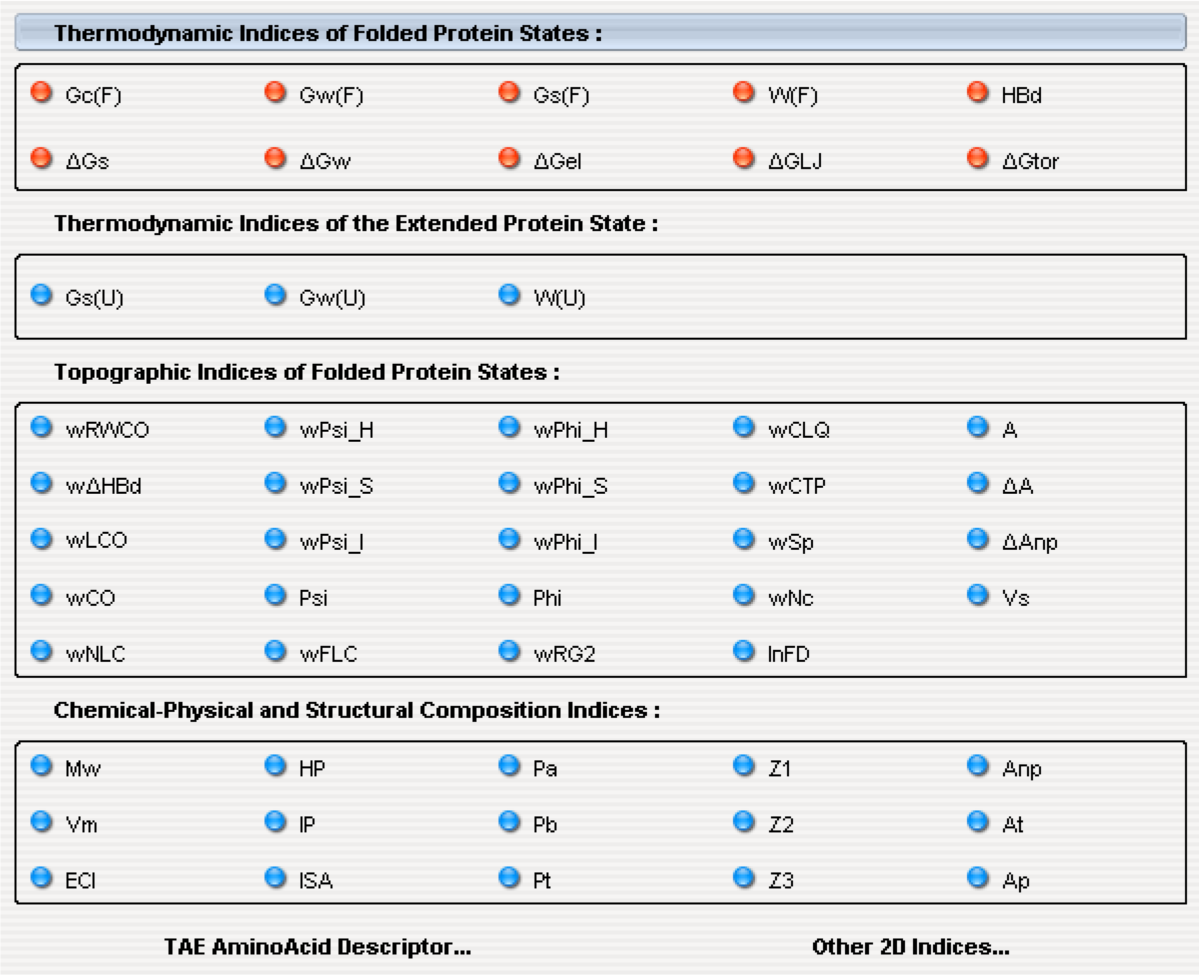

In the Topographic Indices subpanel, there are several weighted indices (starting with the letter "w"), that can be calculated using one or several weights for inter-residue contacts. The following figure shows the window intended for selecting the properties to be used as weights for contacts. This window appears every time one of these weighted indices is selected, in such a way that different properties can be selected for different indices. Alternatively, if many indices will use the same weighting properties, one could first select all the topographic indices at once by clicking the button 'Topographic Indices of Folded Protein States', which launches the properties window once, and then the user may deselect the non-desired indices. These indices will be identified in the outputs as follows: *"index_name(weight)"*.

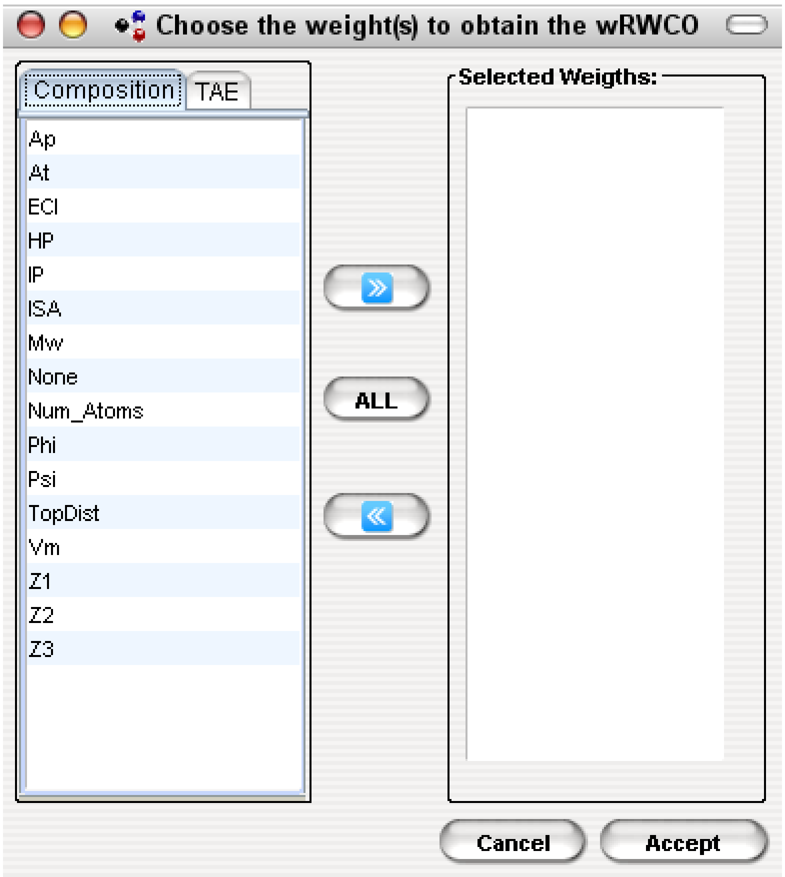

Other indices can be computed using the *"TAE Amino acid Descriptor..."* and "Other Indices..." buttons located at the end of the panel. The first option, calculates the Transferable Atom Equivalent (TAE) indices, which are available in: http://reccr.chem.rpi.edu/Software/Protein-Recon/TAE.doc.

The second option computes user-defined properties (see Creating new Properties) using the *"Define new indices"* option located in the menu *"Option/Manage Indices".* This option activates the following window:

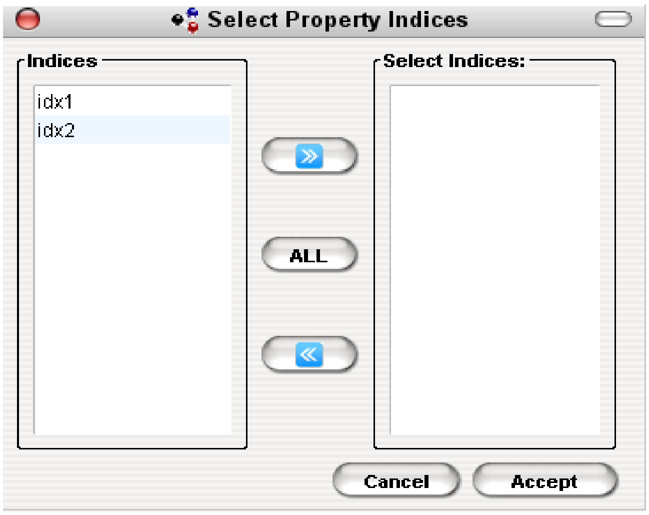

In this window the buttons: 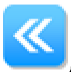, **ALL** and 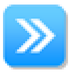 help to select previously defined indices.

Groups Panel

In the panel of groups there are three subpanels enclosing groups formed by residue ID, chemical-physical properties and topographic features. Also with the button: *"Others groups..."*, is possible to select previously defined groups (see Define new groups).

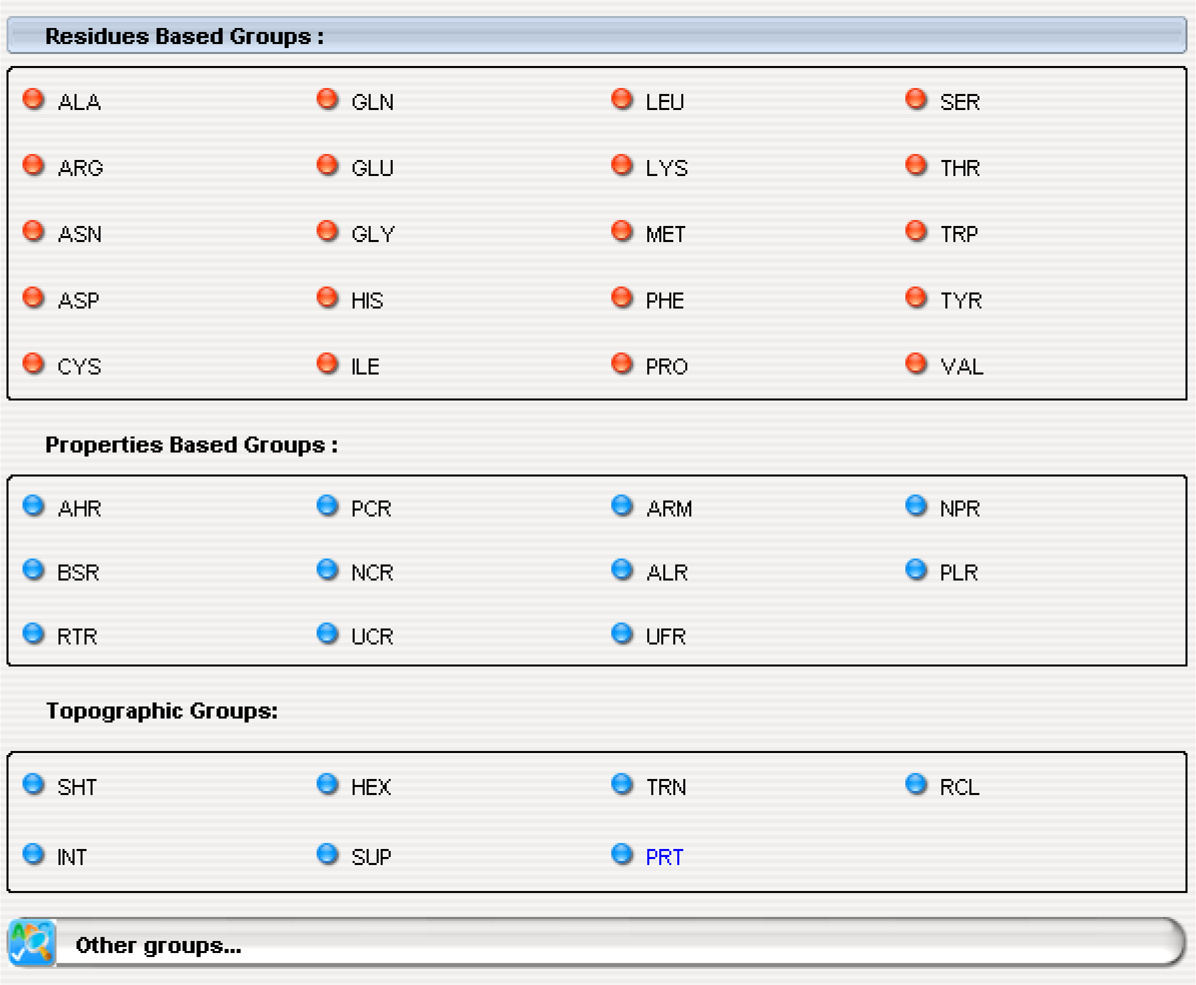

Aggregation Operators Panel

The panel of aggregation operators is divided into four categories: distances, central tendency, dispersion and information theoretic metrics.

In the central tendencies subpanel, specifically for harmonic and geometric means, there are three implemented variants to evaluate these metrics in order to avoid possible in definitions associated with ‘0’ values:

- IGNORE THE VALUES ‘0’

  With this option all zero values are excluded only from the operations, but not in the analysis (i.e. the zero elements are counted to obtain the value N, that refers to the size of the sample).

- PRINT -9999

  This option prints the value -9999 for missing values. In the case of the geometric mean, this occurs when the group size is zero, however, in the case of the harmonic mean, it occurrs when any of the elements are zero.

- DELETE THE VALUES ‘0’

  With this option all values zero are excluded and are not taken into account when dividing by N nor when evaluating the N*th*-root.

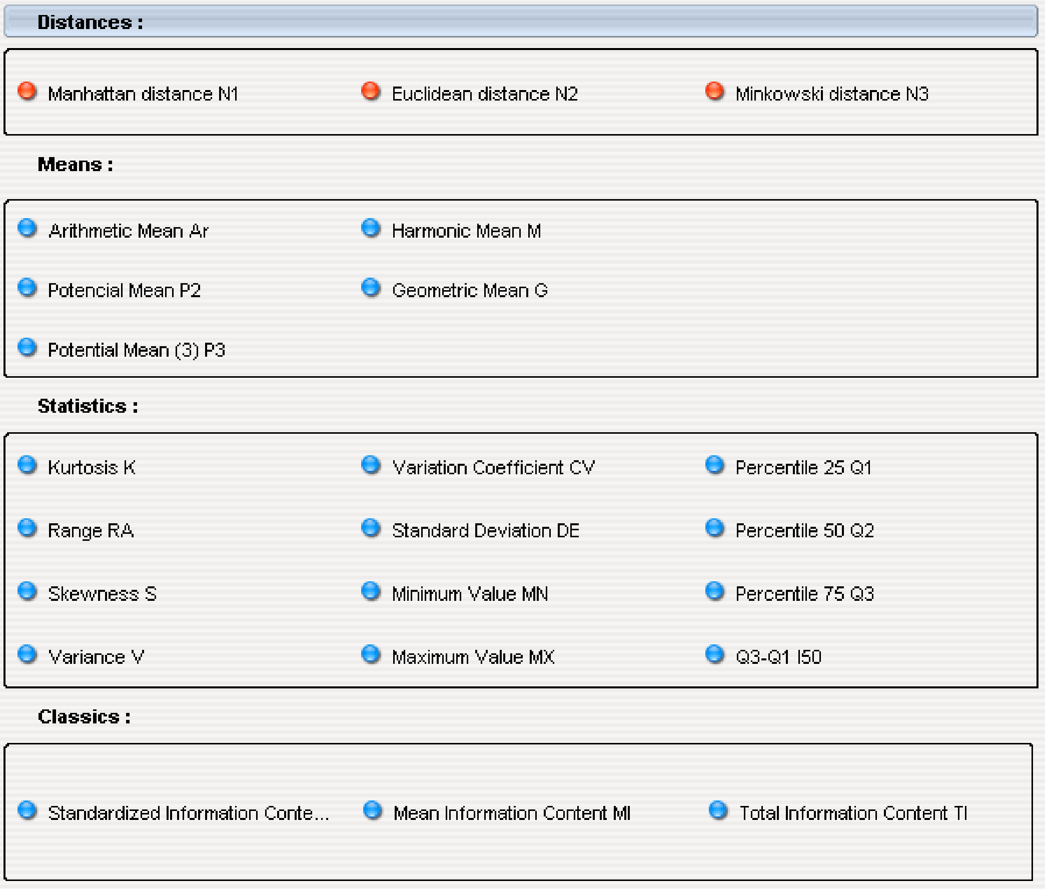

DESCRIPTION OF MENUS

File: This menu allows uploading and/or exporting the different files that are used by the program, e.g. projects and input or output files.

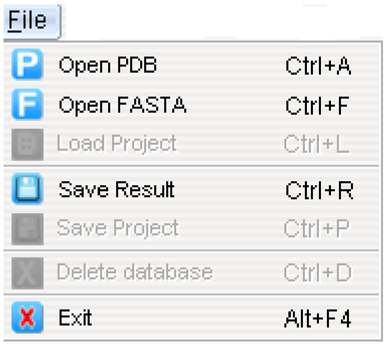

Loading either FASTA or PDB file can be performed by clicking on a buttons F or P respectively, which are located in the toolbar or the 'File' menu. These buttons launch an explorer to select the files to upload.

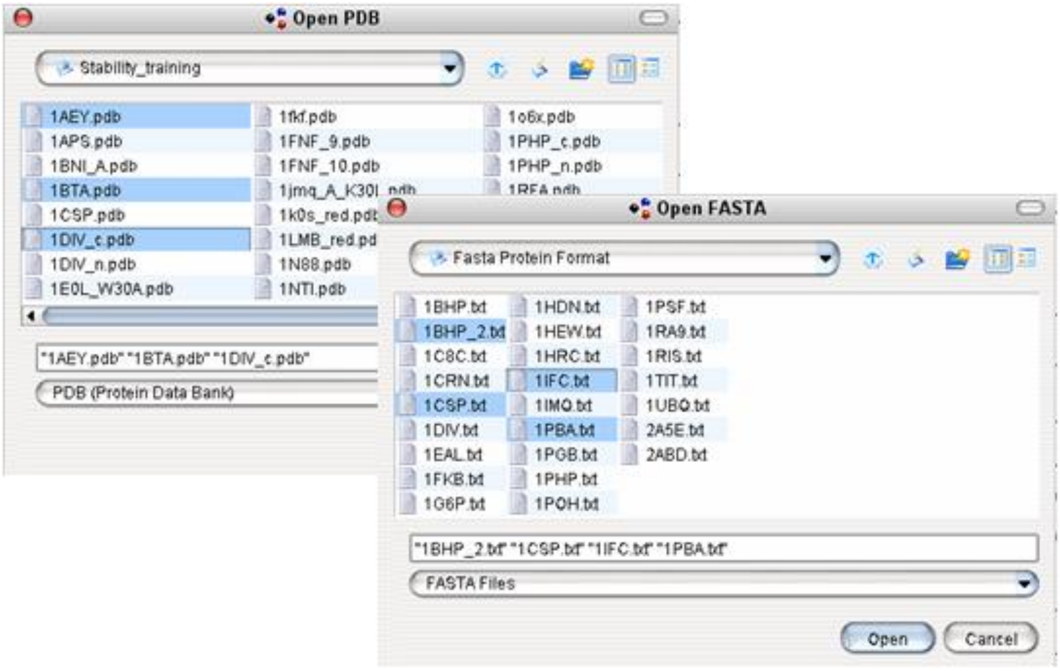

Options: This menu permits configuring the parameters used to evaluate the indices, fixing the amount of significant digits in the output files, <u>and particularly the selection of the modification operator (*Windex: weighted index*) to be applied to the computed residue indices</u>. After the application of this operator, the indices values are updated and the subsequent procedures (grouping and aggregation) make use of these new

indices values instead of the original unmodified indicies. Note that the selected operator will be applied to all selected indices in the same manner. To evaluate different operators, a separate execution needs to be configured (rerun the GUI or save & execute multiple projects using the different operators in batch mode . In addition, the Option menu permits defining new indices and grouping criteria.

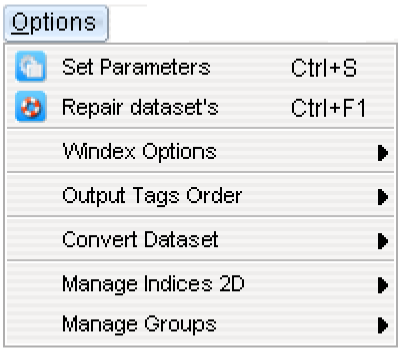

Functions: In addition to protein descriptors, ProtDCal implements the calculation of empirical thermodynamic and kinetic functions: folding free energy (ΔG_{fold}), configurational free energy (ΔG_{conf}), hydrophobic effect (ΔG_{wat}), H-bond deficit free energy (ΔG_{HBd}), close-packing interactions (ΔG_{cpack}), scoring function for structural decoys (ΔG_{scr}), as well as the logarithm of the folding rate constant lnk_f.[1-3]

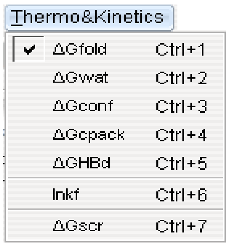

Analyze: This menus gives access to three options to compare a set of protein structures or sequences:

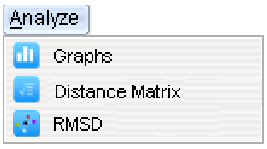

First, one can plot profiles of indices and bar graphs according the distribution of a given index along a sequence.

Profile Graph:

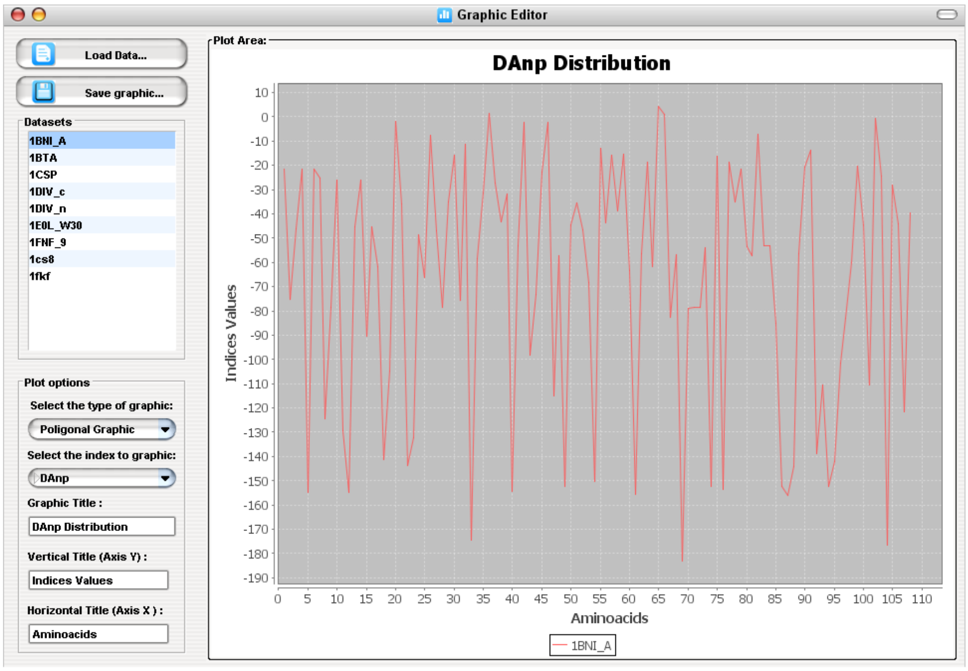

Bar graph (absolute frequency of the index values in different ranges):

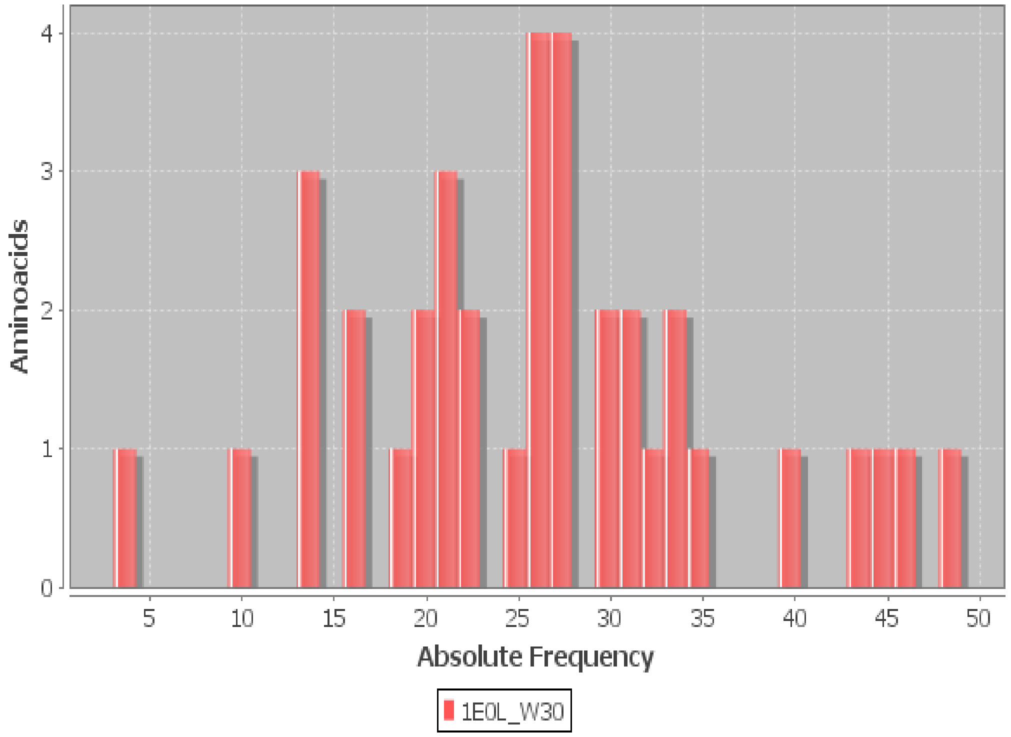

Distance Matrix: This option permits one to compute descriptor-based distance matrices among all proteins in an output file. This option compares different proteins by using previously computed descriptors. This process calculates a distance value

(using either Manhattan, Euclidean or Minkowski (p=3) distances) between all proteins using standardized values of the available descriptors.

This option is configured using the following interface:

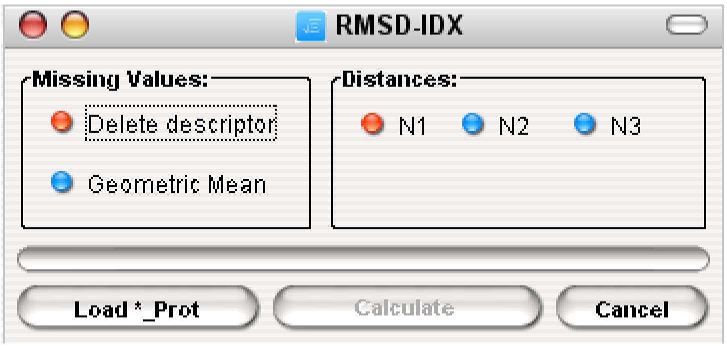

The distance matrix is computed from a file <name>_Prot which must contain only the features which are going to be used to evaluate the distance metrics.

The panel 'Missing Values' provides two options to deal with such data that ProtDCal labels as -9999:

- **Delete the descriptor:**

  Removes the descriptor that contains at least one missing value

- **Geometric Mean:**

  Replaces missing values (-9999) with the geometric mean of the other values of the descriptor.

The 'Analyze' menu also implements a Root Mean Squared Deviation RMSD calculator which uses the Kabasch algorithm, as implemented in the CDK (Chemistry Development Kit) library, to obtain the optimum structural alignment between protein conformations and the selected target. The RMSD can be evaluated among Cα, backbone or all the atoms of the proteins. This option is configured at:

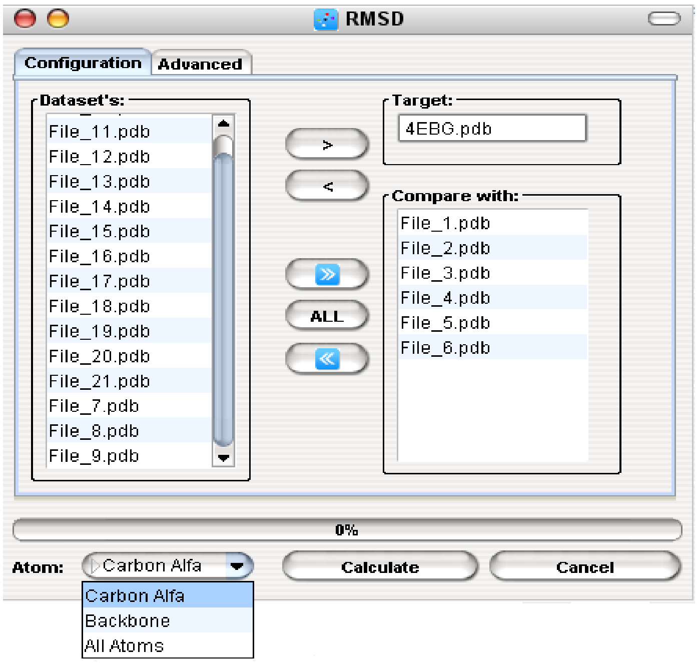

In the 'Advanced' panel, other options are available such as:

- **"Use the best N-residues":**

  This option performs two iterations, one to superimpose the structures considering all the residues, and the second only considers the N-best aligned pairs of residues to re-build the superposition and compute the RMSD.

- **"Use specific ranges":**

  This option superposes and computes the RMSD among structures using specific ranges of residues from the target, the decoys, or all the structures.

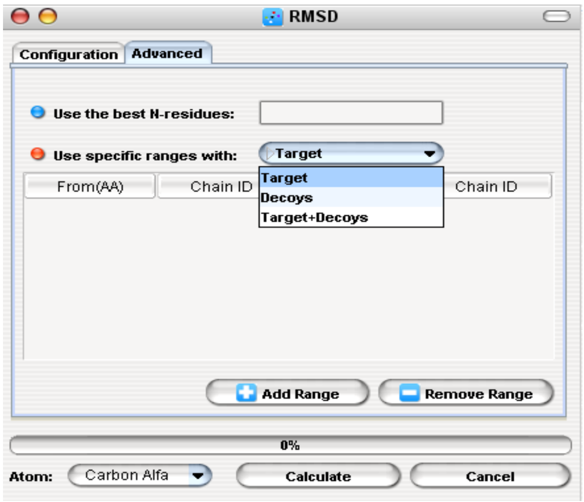

OUTPUT FILES

The button 'Set output for Results' (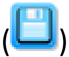) in the toolbar or in the 'File' menu allows one to set the file path to save the results of a calculation. This button launches an explorer to set the path and name of the output files.

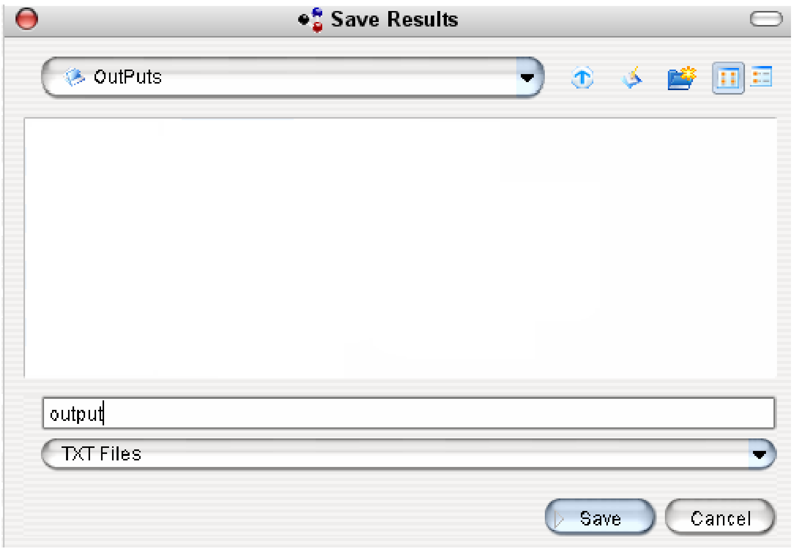

Two files result from ProtDCal calculations: <name>_AA.txt and <name>_Prot.txt. Given the input proteins, these files include the values of the descriptors for each residue, and for each selected group, respectively.

The structure of the file <name>_AA.txt contains, in the second line, the parameters used for calculations, while the third line has the labels of the requested indices. The first column (labeled 'AA') represents the identifier of each residue in the proteins. This column is a combination of protein name, chain identifier, residue name, and residue number from the PDB file. The figure below depicts an example of this type of file.

****************************PARAMETERS************************************								
	t_cont: 4.0	n: 3.0	a: 5.0	dHSG: 9.4	s_cont: 8.0		Windex: None	
AA	A	DA	DAnp	wSp(ECI)	InFD	wDHBd(ECI)	wNc(ECI)	wFLC(ECI)
1BNI_A.pdbAVAL3	193.908098	47.9091607	-21.7193762	0.07	1.47141616	0	0.04655	3.48E-04
1BNI_A.pdbAILE4	77.7314453	-95.0528708	-75.0992704	0.09	0.8783326	0.09	0.0702	2.22E-04
1BNI_A.pdbAASN5	15.3521779	-129.518355	-46.1175002	1.31	1.65278025	0.655	3.49115	0.00114354
1BNI_A.pdbATHR6	74.666298	-55.2963374	-21.5960033	0.65	1.36608813	0.325	0.47775	0.00352778
1BNI_A.pdbAPHE7	29.9173541	-170.276232	-154.915144	0.14	1.57064057	0.07	0.1449	7.70E-04
1BNI_A.pdbAASP8	87.7524386	-48.07673	-21.6235684	1.25	0.90887772	0.625	0.78125	0.00958327
1BNI_A.pdbAGLY9	22.6387094	-46.4846192	-25.4258188	0.02	0.75591333	0	0.0212	3.13E-04
1BNI_A.pdbAVAL10	0	-145.998937	-124.305008	0	1.16260555	0.035	0.07805	0.00106801
1BNI_A.pdbAALA11	0	-103.188938	-78.6799473	0	1.46365202	0.025	0.08525	6.55E-04
1BNI_A.pdbAASP12	76.5502064	-59.2789622	-26.0952002	1.25	0.74344111	0	1.1875	0.01570328
1BNI_A.pdbATYR13	49.2110854	-162.280424	-129.281317	0.72	0.66711186	0.36	0.72	0.00770609
1BNI_A.pdbALEU14	1.06912325	-177.856716	-154.957145	0	1.01745402	0.005	0.01205	1.25E-04

Similarly, the output file <name>_Prot.txt contains, in the first line, the labels of all computed descriptors, which are a combination of the indices, groups, and aggregation operators selected in the main interface. The figure below depicts an example of this type of file.

PDB_NAME	Gc(F)_ALA_N1	Gc(F)_ALA_N2	Gc(F)_ALA_N3	Gc(F)_ARG_N1
1BNI_A.pdb	-0.262927909	0.108934115	-0.085653346	-0.087155006
1BTA.pdb	-0.44563367	0.310293383	-0.300779168	-0.04167399
1CSP.pdb	-0.115023833	0.053122428	-0.041917634	-0.012742242
1DIV_c.pdb	-3.31932248	2.287804715	-2.142282257	-0.019688557
1DIV_n.pdb	-0.228598938	0.101913987	-0.083279311	-0.004512168
1E0L_W30.pdb	-1.010471731	0.818014395	-0.794305205	-0.023512936
1FNF_10.pdb	-0.234815222	0.129980819	-0.112824437	-0.239703689
1FNF_9.pdb	-0.192847766	0.122683904	-0.108209841	-0.2576567
1LMB_red.pdb	-0.689933543	0.435129853	-0.426377415	-0.349460384
1N88.pdb	-0.485642216	0.272855077	-0.258174742	-0.045833105
1NTI.pdb	-0.691944915	0.477533867	-0.467557588	-0.006024037

PARAMETERS CONFIGURATION

The interface to set the parameters to be used in a calculation can be launched using the toolbar button "Set Parameters" ( )or via the 'Options' menu.

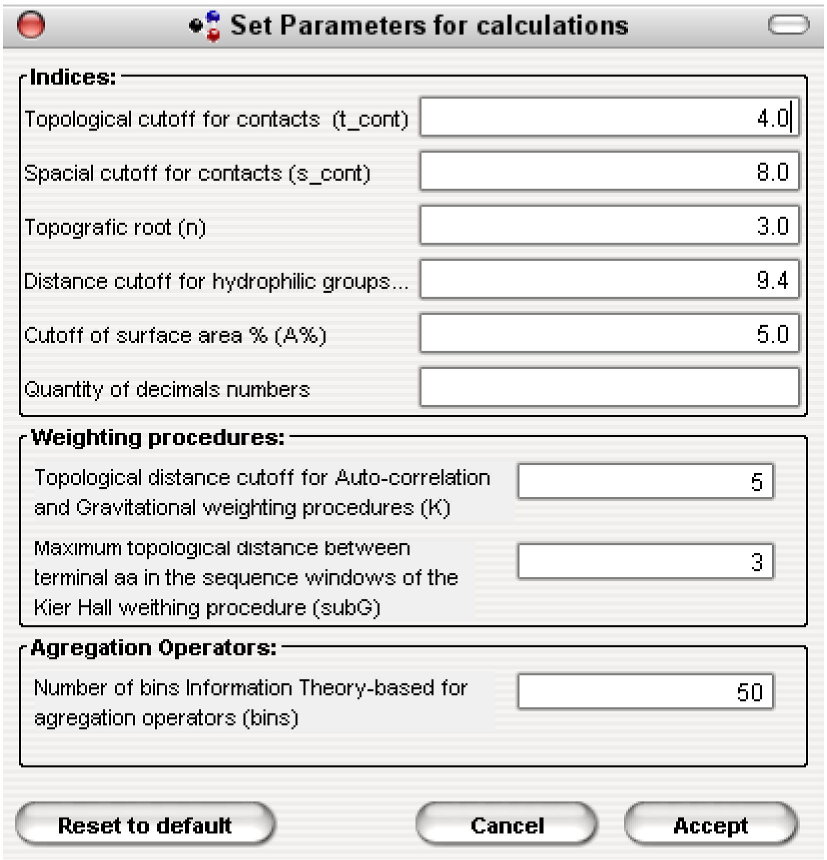

Organizing the output file

The feature or raw matrix obtained after calculation in the output file <name>_Prot.txt is a block matrix that, by default, organizes the descriptor in the hierarchic order "*index > group > aggregation operator*" (invariant). To change the order in this output file, the 'Output Tags Order' button located in the 'Option' menu, provides two options: <index>_<group>_<invariant> (default) and <group>_<index>_<invariant> (alternative).

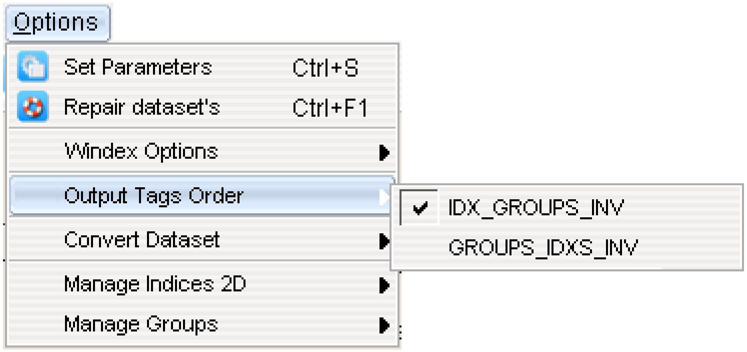

PROJECTS

Projects are text files in which all the options required to execute a calculation are included. To configure a project , one must set all the options of a calculation (i.e. loading data set, indices, modification operators, groups, aggregation operators, and parameters) then the project can be exported by using the button 'Save Project' (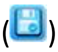) located in the toolbar. The path to the dataset will be kept as part of the project.

Project Structure

A ProtDCal project consists of several tags that identify each of the configuration parameters for a given calculation. The structure of a project is divided into seven sections:

A) Path of the directory containing the input file(s). This section comprises two lines as is illustrated below:

directory:

F:\WORK\RESEARCH\ProtDCal\Datasets\Fasta_Protein_Format\prediction

B) This section summarizes the tag of each selected indices separated by commas:

indices:

Gw(U),Gs(U),W(U),Mw,HP,ECI,Vm,Z1,Z2,Z3,ISA,Pa,Pb,Pt,

When using weighted topographic indices (wIdx), such as the weighed Contact Order (wCO), additional lines are needed to specify the selected weights (separated by comma) for each weighted index:

indices:

A,DA,DAnp,wSp,lnFD,wR2,wDHBd,wNc,wFLC,wNLC,wCO,wLCO,wRWCO

wCO:

ECI,HP,IP,ISA,Mw,None,Num_Atoms,

wSp:

ECI,HP,IP,ISA,Mw,None,Num_Atoms,

wR2:

ECI,HP,IP,ISA,Mw,None,

wDHBd:

IP,ISA,Mw,None,Num_Atoms,

wRWCO:

ECI,HP,IP,Num_Atoms,

wNc:

HP,IP,ISA,Mw,None,Num_Atoms,

wNLC:

ECI,HP,IP,ISA,Mw,

wLCO:

ECI,HP,IP,ISA,Mw,None,Num_Atoms,

wFLC:

ECI,HP,IP,ISA,Mw,None,Num_Atoms,

C) This third section uses two lines to specify the functions' tags separated by commas:

functions:

DGfold,DGconf,DGHBd,

Each function corresponds to one of the models enclosed in the menu 'Thermo&kinetics', which correspond to the empirical thermodynamic model defined in our laboratory to describe protein folding stability and kinetics.

D) The fourth section comprises two lines specifying the groups of residues selected for calculation. Each group's tag is listed separated by comma:

groups:

ALA,GLY,HIS, PHE,ARM,PLR,NCR,SHT,HEX,TRN, INT,SUP,PRT,

Additionally, if a user creates and selects a new group of residues, the defined label is added to the list of other groups:

groups:

ALA,GLY,HIS, PHE,ARM,PLR,NCR,SHT,HEX,TRN, INT,SUP,PRT,USER-1,USER-2

E) This section summarizes the invariant aggregation operators selected to be applied on each group of residues. Each operator's tag is listed separated by comma:

invariants:

N1,N3,Ar,P2,M,V,CV,Q3,K,Q1,DE,MI,

F) This section specifies the parameter values needed to evaluate the indices and invariant aggregation operators. The parameter values are listed as follow:

parameters(t_cont,s_cont,A%, HydGroup,n,bins,K,SubG):

4.0,8.0,5.0,9.4,3.0,50,5,3

These parameters adopt default values. We do not recommend changing the numbers unless the user has an advanced knowledge of its influence on the requested features. Please contact the authors for further direction regarding this subject. The following table provides a brief description of the parameters.

**t_cont**	Topological cutoff for inter-residue contacts. Minimum value of sequence separation between pairs of residues in contact.
**s_cont**	Spatial cutoff for inter-residue contacts. Maximum value of distance between the Cα of pairs of residues in contact.
**A%**	Cutoff of superficiality. Minimal percent of the total surface area of a given residue for being labeled as superficial.
**HydGroup**	Distance cutoff to identify hydrophilic groups of residues. This parameter is used by the thermodynamic indices: Gw(F), DGw, W(F). Its value must vary between: [7.6 - 10.6].
**n**	This parameter is used in the index: 'logarithm of the Folding Degree' (lnFD), as the order of the power to which the spatial distance, between the Cα of a pair of residues, is raised to compute their 'compaction' (quotient between the sequence separation and a power of the spatial distance)
**bins**	Number of bins to compute Shannon-entropy-based information theoretic aggregation operators. The user should fix this value such that that the number of residues per selected group is larger than the number of bins.
**K**	Parameter used by the Autocorrelation and Gravitational modification operators. This value corresponds to the sequence offset to identify the residues used to modify the initial value of the index. For example, when computing the autocorrelation modification for residue position *i*, each index will be affected by the residue at position *i* ± 5.
**SubG**	Parameter used by the modification operator: Kier-Hall. This value corresponds to the maximum length of the sub-graphs (of path type) used to modify the value of a given residue. For example, for a value of 3, all the sub-graphs of no more than 3 residues and containing the residue *i* are used to modify its value.

G) This last section summarizes the value of other general options of project:

options(decimals,armonicMeanType,geometricMeanType,windexID,datasetType,outputOrder):

-1,0,0,-1,true,true

Where:

**decimals**	Amount of decimals numbers to use in the output file (-1: no approximation is done).
**harmonicMeanType**	Specify the options to deal with the zeros when computing the Harmonic Mean.
**geometricMeanType**	Specify the options to deal with the zeros when computing the Geometric Mean.
**windexID**	Specify the modification operator to be use in the calculation. Where: 0 = Autocorrelation, 1 = Gravitational, 2 = Kier-Hall, 3 = Ivanciuc-Balaban, 4 = Electrotopological State, -1 = none
**datasetType**	Type of input files. True: PDB files. False: FASTA files
**outputOrder**	Order of the block matrix of features in the output file: True: IDX_GROUP_INVARIANT. False: GROUP_IDX_ INVARIANT

NOTE: A project must not contain any empty lines or incorrect tags. It is strongly recommended to use the graphical user interface to configure the project initially. What follows is a list of valid tags for each section:

==ProtDCal Indices tags==

===>Thermodynamic Indices of Folded Protein States

Gc(F),Gw(F),Gs(F),W(F),DGs,HBd,DGel,DGw,DGLJ,DGtor

===>Thermodynamic Indices of the Extended Protein State

Gw(U) Gs(U) W(U)

===>Topographic Indices

A,DA,DAnp,wSp,lnfd,wR2,wDHBd,wNc,wFLC,wNLC,wCO,wLCO,wRWCO,wCTP,wCLQ, wPsiH,wPsiS,wPsiI,wPhiH,wPhiS,wPhiI,Phi,Psi

===>Property Based Indices

Mw,HP,IP,ECI,Vm,Anp,Z1,Z2,Z3,ISA,At,Ap,Pa,Pb,Pt

==ProtDCal Functions tags==

DGfold,DGwat,DGconf,DGpack,ln(kf),DGscr,DGHBd

==ProtDCal Groups tags==

===>Residue Basic Group

ALA,ARG,ASN,ASP,CYS,GLU,GLN,GLY,HIS,ILE,LEU,LYS,MET,PHE,PRO,SER,THR,TRP,TYR,VAL

===>Properties Based Group

RTR,BSR,AHR,ALR,NPR,ARM,PLR,PCR,NCR,UCR,UFR

===>Topographic Group

SHT,HEX,TRN,RCL,INT,SUP,PRT

==ProtDCal Procedural Aggregation_operators ID==

===>Distances

N1,N2,N3

===>Means

Ar,P2,P3,M,G,V

===>Statistics

CV,Q3,S,RA,MN,K,Q1,MX,DE,Q2,I50

===>Information Theoretic Operators

SI,MI,TI

Below is a screenshot showing the structure of an actual project:

project_example.proj

```
directory:
F:\WORK\RESEARCH\proteins\ProtDCal\Datasets\Pdb Protein Format
indices:
DGw,Gw(U),Gs(U),W(U),A,lnFD,wCTP,HP,wPsiS,Phi,Psi,Pa,Pb,
functions:
DGfold,DGconf,DGHBd,
wPsiS:
Z1,Z3,HP,IP,At,
wCTP:
Z1,Z3,HP,IP,At,
groups:
ALA,GLU,GLY,HIS,MET,PHE,ARM,PLR,NCR,SHT,HEX,TRN,RCL,INT,SUP,PRT,
invariants:
N1,N3,Ar,P2,M,V,CV,Q3,K,Q1,DE,MI,
parameters(t_cont,s_cont,area,dHSG,n,Int,K,Subgraph):
4.0,8.0,5.0,9.4,3.0,50,5,3
options(decimals,armonicMeanType,geometricMeanType,windexID,datasetType,outputOrder):
-1,0,0,-1,true,true
```

Loading a project

To load a project use the button 'Load Project' ( ) located in the toolbar. This button will launch an explorer to select the desired project.

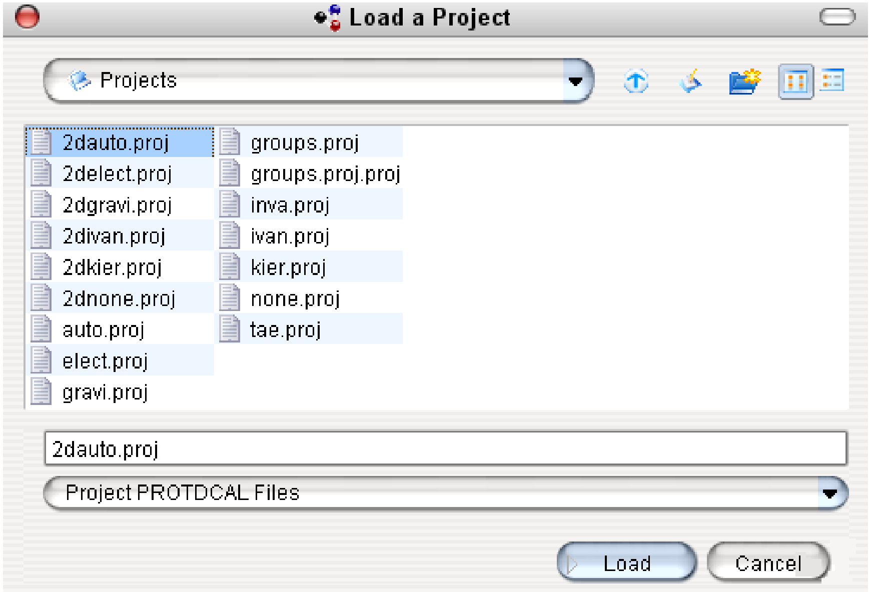

USER-SPECIFIED INDICES

The button 'Define new Property' located in 'Options' menu permits the definition of specific property-based indices. This option will launch the following window:

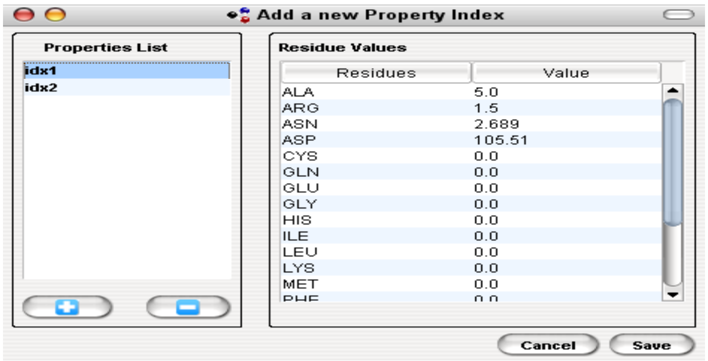

The panel 'Property List' provides the list of available indices. The 'Residue Values' panel permits editing the assigned values to each residue.

When defining new indices, the option "Select new Property", in the 'Option' menu, permits selecting these indices for calculation.

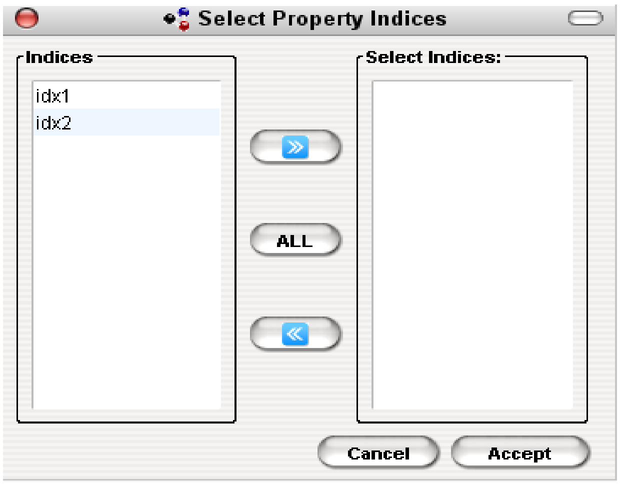

USER-SPECIFIED GROUPS

To create new groups select the *"Define new group"* (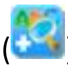) located in the menu "Options/Managing Groups" which will launch the following window:

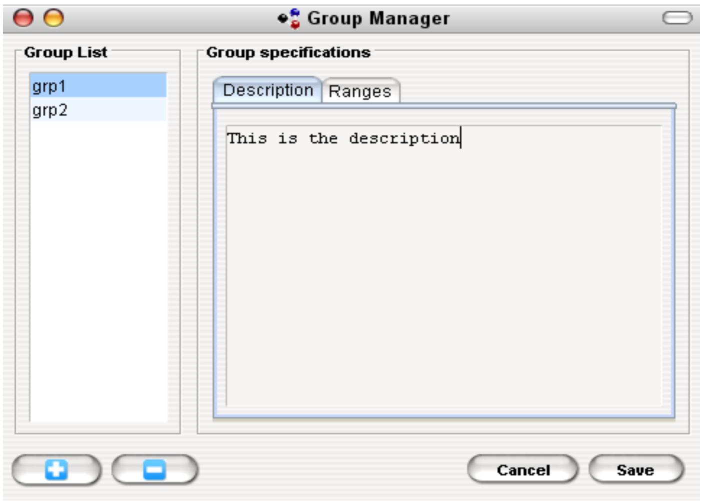

This option allows the definition of new groups of residues. These groups are created by extracting specific ranges of residues that can be fixed using panel ‘Ranges’.

The ranges can be configured by settling the position of the initial and last residues as well as the identifier of the chain of each residue.

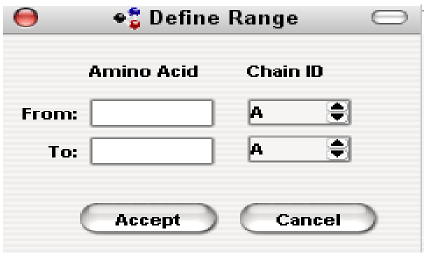

The option *'Select groups'* ( ) permits selecting these new groups, for subsequent calculations, through the following interface:

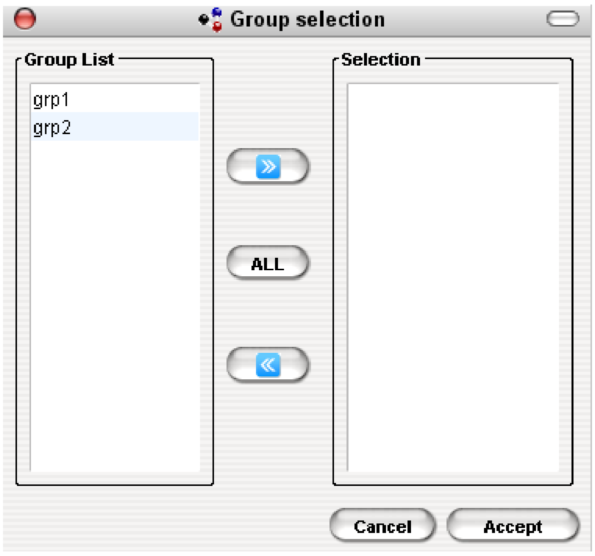

EXECUTING CALCULATIONS

ProtDCal permits carrying out a single calculation or running multiple projects in batch mode. The first option can be accessed directly by configuring a set of indices, groups, and aggregation operators. Additionally, it can be executed by uploading a single predefined project.

To execute several projects in batch mode, the button 'Run Projects' ( ) located in the toolbar, permits one to select a set of predefined projects through the following interface:

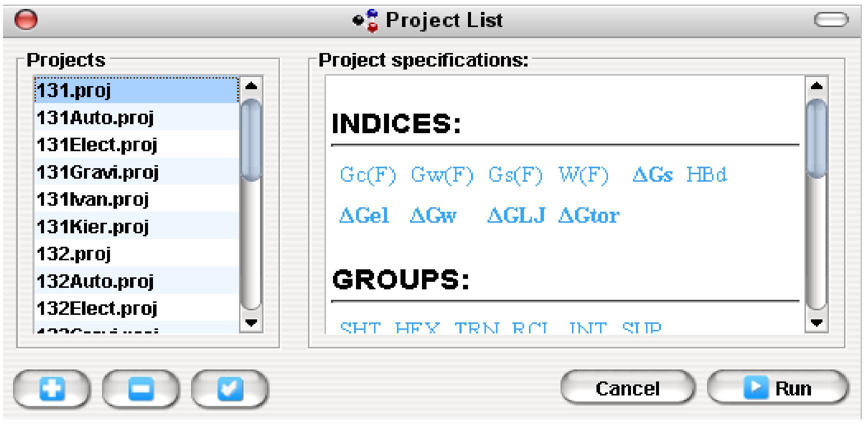

Alternatively, if a number of Projects are configured the user can execute ProtDCal in console mode as:

java –Xmx1000m –jar ProtDCal.jar –p <Path to projects' directory> -o <path to outputs' directory>

If no option is specified this line will simply execute the graphical user interface.

ProtDCal's command-line options:

-p: Defines the path to the directory enclosing the projects to execute. All projects in this directory will be computed.

-o: Defines the location in which to create the output files. Each file will take the same name as the corresponding project.

-v: Defines whether to include the name of the project within the label of final descriptors. 0: no (default), 1: yes. This option is valuable when the same descriptors are computed, but different parameters are evaluated each time (likely of interest only to advanced users).

BASIC MODELING WORKFLOW USING PROTDCAL AND WEKA

ProtDCal is intended to generate a wide variety of features describing a protein sequence and/or structure. By applying feature selection, an appropriate feature subset may be identified and used to create effective classifiers. Below, we detail the creation of a predictor of N-linked glycosylation based on protein sequence.

Prediction of N-linked glycosylation from protein sequence

Gathering the data set of instances:

3508 sequence-unique windows of 15 aa, each centered on an Asn residue, were extracted from the 242 protein sequence targets of O-GLYCBASE. This data set can be found, in FASTA format, within the 'Datasets' directory in the ProtDCal distribution with the name 'glyco-3508.fasta'.

Generation of an initial set of features:

It is known from the literature related to N-glycosylation that this process is highly sensitive to the presence of specific amino acids at specific positions near the target Asn residue. The most commonly used sequence motif associated with N-linked glycosylation is defined by the "sequon": Asn-Xxx-Thr/Ser, which indicates the strong influence of a Thr or Ser residue at position Asn + 2. Therefore, it was decided to generate position-specific features for all the analysed sequence windows.

Please see the section 'User-specified groups' of this manual in order to learn how to define such groups. User-specified groups are saved in a text file named 'groups.gdm' that appears in the main directory of ProtDcal distribution. Each newly defined group is saved in this file using the following format:

```
RangeGroup <name>
<Comment line>
```

n 0 *n* 0
END Group

These four lines are summarized as follows: i) the name given to the group, ii) an optional description, iii) the starting and final position of an inclusive range of residues gathered in the group (where *n* 0 *n* 0 means: the nth residue of the first chain to the nth residue of the first chain), and iv) a marker ending the section of this group. This file can be edited directly by the user without the need of using the graphical interface. Fifteen new groups were defined, each corresponding to exactly one residue position within the 15 aa windows. These were named '1' through '15'.

A number of residue indices were then selected to be computed for each of the 15 groups. These indices comprised distinct properties and thermodynamic indices, using the Kier-Hall modification operator (with a sub-graph parameter of '1') and the Minkowsky norm 'N1' as the aggregation operator. These options can be specified using the graphical user interface or by manually creating of a project file with the following information (the comment text in *green-italics* is added here to explain each line, but should not appear in the actual project file):

//path to input sequence window files
directory:
<Path to input sequence windows files or multi-FASTA file gathering all the sequence windows>
//which indices to compute for each group
indices:
Gw(U),Gs(U),W(U),HP,IP,ECI,Z1,At,Pb,
//which groups to use – defined in 'groups.gdm'
groups:
1,2,3,4,5,6,7,8,9,10,11,12,13,14,15,
//specify aggregation operator to use
invariants:
N1,
//default parameter values
parameters(t_cont,s_cont,A%,HydGroup,n,bins,K,SubG):
4.0,8.0,5.0,9.4,3.0,50,5,1
//default options used
options(decimals,harmonicMeanType,geometricMeanType,windexID,datasetTyp
e,outputOrder):
-1,0,0,2,false,true

Finally, by placing this project file in a directory named 'ExampleGly' within the 'Projects' directory, the features can be computed by executing this command line:
Java –jar ProtDCal.jar –p Projects/ExampleGly –o Outputs
This calculation generates two tab-delimited output files named <project name>_AA.txt and <project name>_Prot.txt, which summarize feature matrices in the format [AA vs. residue indices] and [sequence windows vs. features] respectively. We will use the file called <project name>_Prot.txt, which shall summarize the computed features for each sequence window.

Preparing the data file to be read by Weka

Weka can read csv files directly which are easily obtained from the tab-delimited files generated by ProtDCal. Additionally, one must append the class column at the end of each line of the file. This can be accomplished easily, for example, using a spreadsheet program such as MS Excel by pasting the column with the class information after the last column of features. Lastly, the column with the name of the instances should be removed to prevent Weka from interpreting this column as another attribute. Finally, the document must be saved in csv format.

Running filters and attribute selection approaches with Weka:

In order to eliminate some trivial features that could be generated, is recommended to first run the unsupervised Weka attribute filter called 'RemoveUseless':

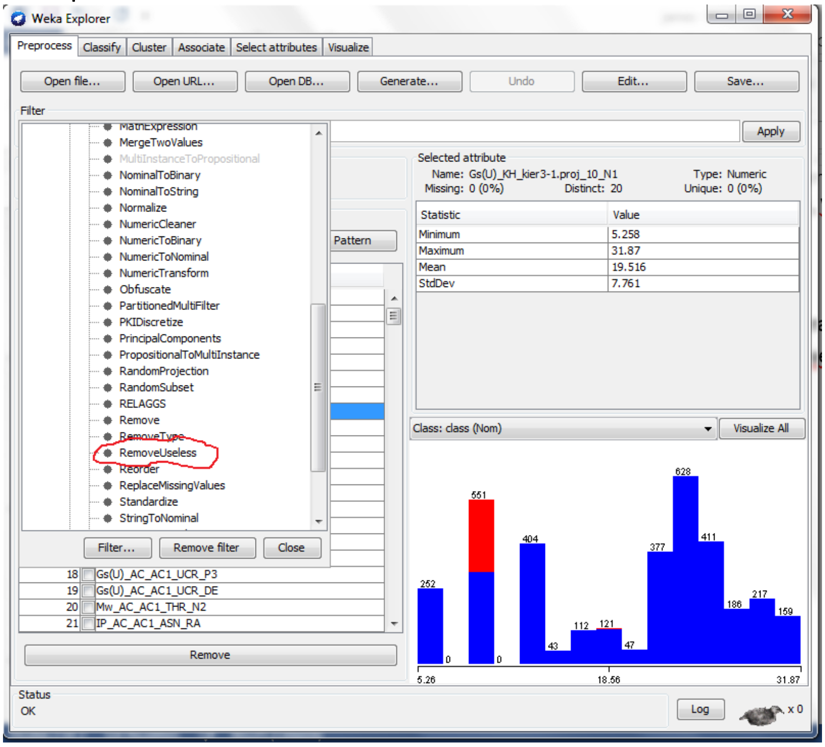

This filter will eliminate all constant attributes that may be generated by ProtDCal following the project file.

Depending on particular interests and the desired number of attributes, other filters can be applied at this stage. It is recommended to perform a supervised attribute selection approach that analyses the relevancy and redundancy of the features. This can be carried out with a wide range of methods implemented within Weka. Here, we use the attribute selection method called 'CfsSubsetEval' coupled with the 'Bestfirst' search method. The reduced data set can be obtained by right-clicking on the report name at the left panel of the windows and selecting 'Save reduced data':

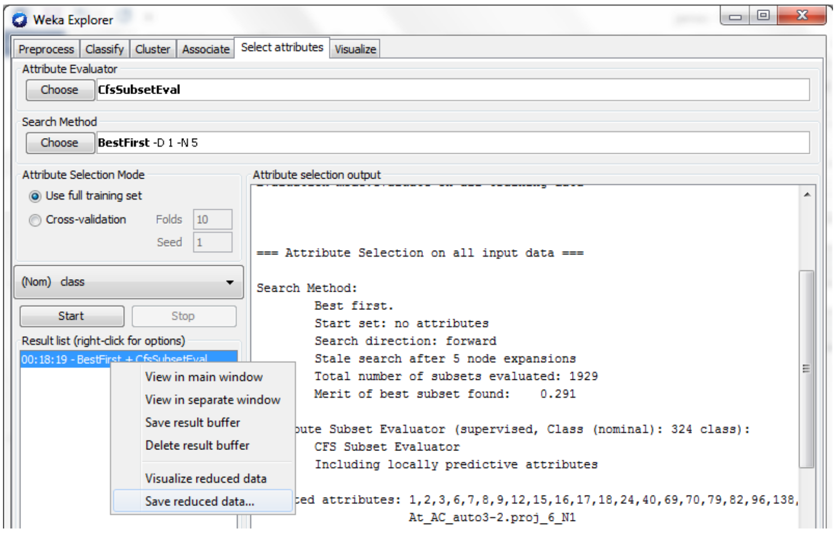

After uploading this reduced subset of features, it is advisable to end by running the 'WrapperSubsetEval' attribute selection approach. Depending on the number of features remaining in your data file, a genetic search may be used within the wrapper. However, if the number of attributes is too high (>100), a 'Bestfirst' search would be preferable for a first reduction. The Wrapper should be executed with the same type of classifier that you intend to use to later use to evaluate your final model over the test data. For the study of N-glycosylation presented in the ProtDCal paper, a genetic search with 50 chromosomes per population and 500 generations was conducted. As for the evaluator, a 'FilteredClassifier' was used, which applies a 'Resample' filter to the training data such that a class-balanced subset is sampled for each cross-validation fold. This subset is used to train a classifier (both NaiveBayes and RandomForest were considered) and evaluate it in the hold-out set during the x-fold iteration of the Wrapper.

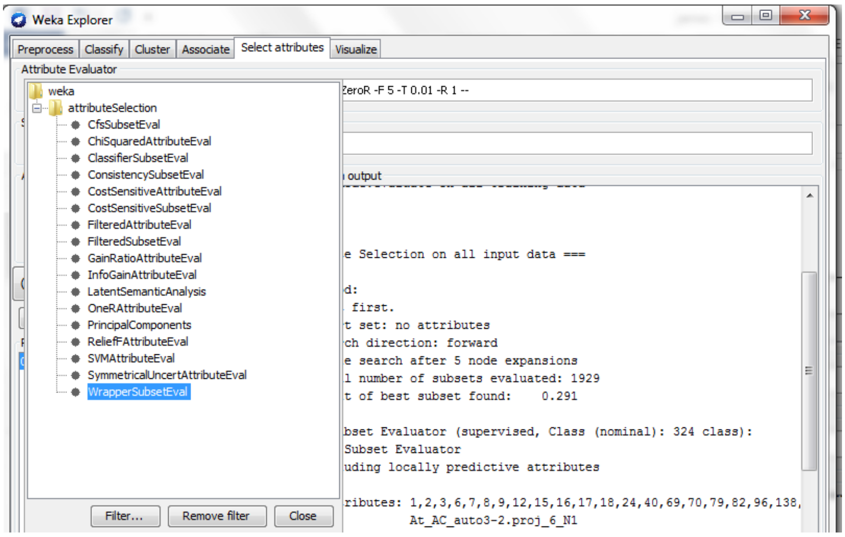

Once the extraction is finished, the reduced subset is saved and used to build the corresponding classifier over all training data using a similar configuration as it was used during the Wrapper. In the 'Classify' panel of Weka there are options to automatically perform x-fold cross-validation, hold-out prediction test by splitting the input data, and external prediction by providing a second set of test instances with the corresponding features and class attribute. This latter option was used to evaluate our

final naïve Bayes and random forest classifiers using the blind test data. The classification accuracy is reported in the “Classifier output” section of the Weka environment.

Finally, the resulting classification model can be saved from the report in the left panel as shown below.

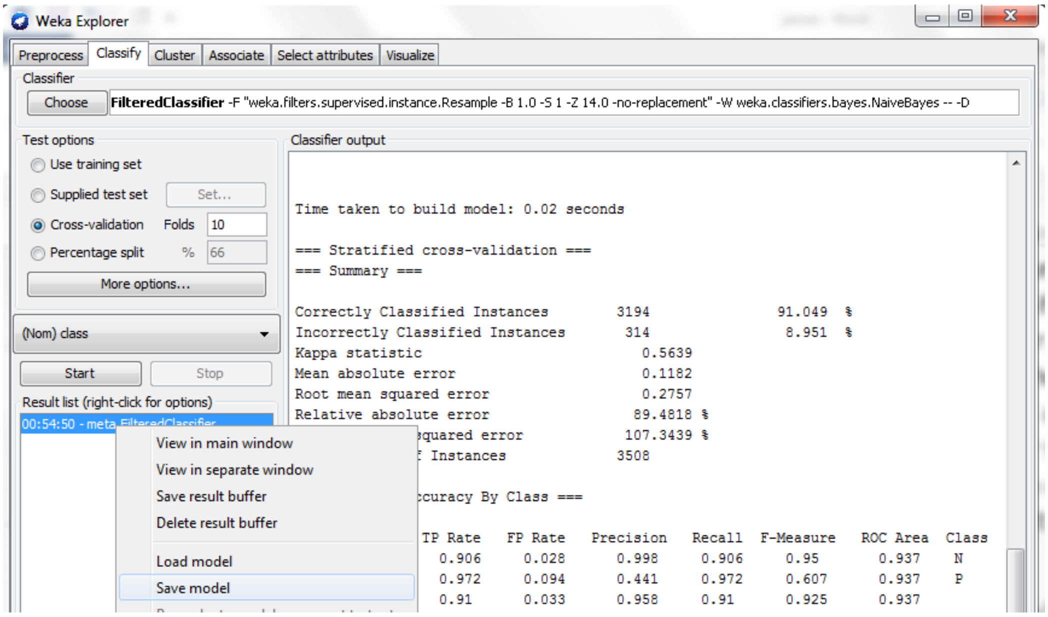

The saved model file can then be used to predict the glycosylation states for any other data set, for which the final features, used in the model, must be previously calculated using ProtDCal. The current (2015) distribution of ProtDCal contains the specific project files to compute each of the features entered in the models described in our report (Y.B. Ruiz-Blanco et al. BMC Bioinformatics, 2015) for N-linked glycosylation.

(1) Ruiz-Blanco, Y. B.; Marrero-Ponce, Y.; Prieto, P. J.; Salgado, J.; García, Y.; Sotomayor-Torres, C. M. *Journal of Theoretical Biology* **2015**, *364*, 407.

(2) Ruiz-Blanco, Y. B.; Marrero-Ponce, Y.; García, Y.; Puris, A.; Bello, R.; Green, J.; Sotomayor-Torres, C. M. *Chemical Physics Letters* **2014**, *610–611*, 135.

(3) Ruiz-Blanco, Y. B.; Marrero-Ponce, Y.; Paz, W.; García, Y.; Salgado, J. *Journal of Theoretical Biology* **2013**, *321*, 44.
